# A Nutritional Evaluation of Plant-Based Meat and Sausage Analogues

**DOI:** 10.3390/foods14213674

**Published:** 2025-10-28

**Authors:** Leah Brodersen, Gerald Rimbach, Ulrike Seidel, Pia Rinne, Mario Hasler, Anja Bosy-Westphal, Katharina Jans

**Affiliations:** 1 Department of Food Science, Institute of Human Nutrition and Food Science, Christian Albrecht University of Kiel, 24118 Kiel, Germanyrimbach@foodsci.uni-kiel.de (G.R.);; 2 Lehrfach Variationsstatistik, Christian Albrecht University of Kiel, 24118 Kiel, Germany; 3 Department of Human Nutrition, Institute of Human Nutrition and Food Science, Christian Albrecht University of Kiel, 24105 Kiel, Germany

**Keywords:** plant-based meat analogues, meat alternatives, meat substitutes, nutritional quality, Nutri-Score, high-protein foods

## Abstract

Plant-based meat and sausage analogues (PBMAs) are gaining popularity due to growing concerns about the health, environmental, and ethical impacts of animal-based foods. In the present study, we compared the nutritional quality of 298 PBMAs to 294 animal-based reference products available on the German retail market in 2024 across nine subcategories. PBMAs generally contained less total fat, saturated fat, and protein, but more fibre, carbohydrates, and sugar compared to meat products. Notably, most meat analogues exhibited higher salt contents. Nutri-Scores of PBMAs were significantly favourable in six subcategories. Among PBMAs, approximately 60% demonstrated good protein quality, while only 12% were fortified with key micronutrients, including vitamin B12 or iron. Most PBMAs contained one or more additives and flavourings. These findings suggest that while PBMAs often offer favourable nutritional profiles, reformulation and clearer nutritional guidance could help to further support the emergence of healthier plant-based options.

## 1. Introduction

With the global population projected to reach ten billion by 2050, global food systems face mounting pressures to provide sustainable and health-promoting diets [1,2]. The current trajectory of animal product consumption contributes significantly to environmental degradation, including greenhouse gas emissions, land and water use, and biodiversity loss [3,4]. Simultaneously, excessive consumption of meat and animal protein is associated with serious health risks, including zoonotic diseases, increasing antibiotic resistance, cardiovascular disease, and cancer [3]. Consequently, the promotion of plant-based diets has emerged as a key strategy for improving public and planetary health [2,5]. Among various strategies, PBMAs offer a familiar and convenient option for reducing meat consumption without compromising on taste and may support consumers in aligning their diets with national guidelines such as the German Nutrition Society’s (DGE) and World Cancer Research Fund (WCRF) recommendations to limit meat and sausage consumption to 300–500 g per week [6,7].

Germany represents one of the largest and fastest-growing markets for plant-based meat and sausage analogues (PBMAs) in Europe, with a significant increase in product diversity and consumer interest [8]. In 2023, over 120.000 tons of meat alternatives were produced in Germany—a 17% increase compared to the previous year [9]. The rapid rise in PBMA consumption has drawn increasing attention and criticism, particularly concerning their nutritional quality compared to conventional meat. Although scientific evidence on their actual nutritional value is still limited, consumers frequently perceive plant-based alternatives as healthier [10]. Research suggests that replacing animal protein, especially from processed red meat, with plant protein is associated with reduced all-cause mortality [11]. However, these products are frequently classified as ultra-processed foods (UPFs) according to the NOVA classification system [12], raising concerns regarding their nutritional adequacy and potential health implications [13]. Additional criticism relates to potentially high salt content and the absence or low levels of essential micronutrients such as vitamin B12, iron, and zinc. Moreover, the protein quality of PBMAs remains uncertain, as plant-based proteins typically contain lower amounts of certain essential amino acids and differ from meat in terms of digestibility and bioavailability [14,15,16].

Despite global growth of the PBMA market [17], knowledge gaps persist regarding how the nutritional profile of PBMAs compares to meat, and whether they can adequately support human health [18]. Most existing studies have been conducted outside of Germany, included only a limited range of PBMAs, or focused exclusively on sensory acceptance, the environmental impact or individual nutrients rather than overall nutritional quality. In many cases, products containing egg or dairy ingredients were also included, and there was often no clear distinction between minimally processed products made from whole legumes or vegetables and modern, highly processed meat analogues.

This study addresses these gaps by providing a comprehensive evaluation of the nutritional quality of modern PBMAs available on the German market. Specifically, it investigates: (1) declared nutritional values of PBMAs and meat products; (2) Nutri-Score classification of plant-based and animal-based products; (3) protein sources and quality; (4) micronutrient fortification; and (5) the use of food additives in PBMAs. By comparing nearly 300 PBMA with animal reference products across nine subcategories, we assessed their potential at the public health level, providing guidance for industrial optimization and reformulation.

## 2. Methods

### 2.1. Data Collection and Classification of PBMAs

From July to August 2024, a total of 298 PBMAs were surveyed in ten major German supermarket and discount chains, representing over 70% of the national grocery market [19,20]. Most data were collected in physical stores by photographing product labels, while additional information was retrieved from publicly accessible online assortments. Products were surveyed in different regions across Germany to ensure geographic diversity.

PBMAs were included if they were (1) explicitly designed to imitate meat or sausage, (2) based primarily on isolated or concentrated plant proteins (e.g., soy protein isolate, wheat gluten), and (3) available in retail form (chilled, frozen, or shelf-stable). Products based on whole legumes, vegetables, or traditional vegetarian foods (e.g., tofu, tempeh, falafel) were excluded, as were seafood analogues, products with animal-based ingredients, raw materials, dry mixes requiring preparation, and ready meals.

Each product was categorized into main groups (meat or sausage analogue) and further divided into subcategories resembling the corresponding meat products (see Table 1). Categorization was based on appearance, labelling, and assumed culinary use. Product names, brands, ingredient lists, declared energy (kJ/kcal) and nutrients per 100 g (per EU Regulation No. 1169/2011), fibre content, and added micronutrients were systematically documented. For products with label values such as “<0.5 g”, the maximum value was recorded. Missing fibre values were estimated based on energy differences. A complete list on the data collection of all products can be found in Tables S5 and S6.

### 2.2. Reference Products

A total of 294 animal-based reference products were selected for comparison. The primary data source was the German Nutrient Database (BLS, version 3.02, https://blsdb.de (accessed on 6 May 2024)), which offers a comprehensive dataset of commonly consumed meat products. To account for deviations from typical market values, particularly in salt content, additional data were obtained from the online assortment of a major supermarket (REWE Markt GmbH) to ensure a realistic assessment of nutrient composition. To maintain comparability, fat- or energy-reduced products were excluded, and the distribution of plant-based products within each subcategory was mirrored in the meat group (e.g., the ratio of spreadable sausage types, cooked vs. raw variants). Red meat products were limited to pork and beef, reflecting the composition of plant-based imitations. Kebab-style products were included among the plant-based analogues; however, no suitable animal-based reference could be incorporated, as neither the BLS nor the online sources provided corresponding entries.

### 2.3. Nutritional Data Analysis

The macronutrient composition per 100 g (energy, fat, saturated fat, carbohydrates, sugar, fibre, protein, salt) of the plant-based and animal-based products was gathered. In addition, the energy percentage derived from protein was calculated. To explore nutrient-based clustering, Principal Component Analysis (PCA) was conducted separately for meat and sausage products, each including subcategories of plant-based analogues and corresponding animal-based products. Fibre content was excluded from the PCA due to its low variability among PBMAs and its near-complete absence in animal-based products, which would have contributed little to the differentiation of subcategories.

### 2.4. Nutri Score Calculation

The Nutri-Score is one of the most widely used front-of-package labels in Europe [21]. While designed for comparing products within the same category, consumers may also perceive it as a tool for comparing products with related alternatives or substitutes [22,23]. While reflecting nutrient composition, the Nutri-Score does not account for ingredients or processing levels, disregards sustainability and level of processing [21]. Nevertheless, it allows for differentiation within ultra-processed food categories and, therefore, remains a valuable and easy-to-understand tool for assessing nutritional quality, especially for the costumer [24]. Nutri-Score ratings were determined using the official algorithm provided by Santé publique France, which assigns a score from A (dark green, most favourable) to E (dark orange, least favourable) based on the FSAm-NPS (Food Standards Agency modified nutrient profiling system) score [25]. Points were assigned for unfavourable nutrients (energy, saturated fat, sugar, salt) and subtracted for favourable components (protein, fibre, and the percentage of fruits, vegetables, and legumes), yielding a range from −17 (most favourable) to +55 (least favourable). The percentage of fruits, vegetables, and legumes was set to zero for all products, as it is only scored from 40% or more, and the PBMAs examined in this study were based on protein isolates or concentrates rather than minimally processed ingredients. According to the new algorithm, for red meat and products derived from it, protein points were capped at a maximum of 2. For meat products likely to be salted during preparation (e.g., minced meat), a standard salt content of 0.5 g per 100 g was added.

### 2.5. Protein Source and Quality Assessment

For each plant-based product, the main protein source was identified based on the first protein-containing ingredient listed. Additional protein sources were recorded to assess potential complementary combinations. Although relative proportions were not evaluated, combinations were analysed with respect to limiting amino acids. Protein quality was estimated using the Digestible Indispensable Amino Acid Score (DIAAS) from scientific literature. Data was searched for on Google Scholar by entering the terms ‘DIAAS’ or ‘protein quality’ combined with ‘AND’ and the respective protein source (e.g., ‘soy’). Based on these values and the presence of complementary sources, products were classified according to their potential to provide a balanced amino acid profile.

### 2.6. Micronutrient Fortification and Additives

Product labels of PBMAs were analysed for micronutrient enrichment (e.g., vitamin B12, iron) and for the presence of additives and flavourings. Paprika extract was only classified as an additive if its colouring function was explicitly stated (e.g., “colouring paprika extract”). Additives were grouped by functional class, e.g., stabilizers, thickeners, colorants and preservatives. No analysis of the animal-based products was conducted, as most of them did not include an ingredient list (e.g., those taken from the BLS, but also due to non-EU conform declarations of some products taken from the market) and many were not ready-to-eat products.

### 2.7. Statistical Analysis

Normality of nutrient data was tested using the Shapiro–Wilk test and visually inspected via histograms and Q–Q plots. As normality was rejected for all variables, non-parametric methods were applied. Differences between plant-based and animal-based subcategories were analysed using the Mann–Whitney U test (*p* < 0.05), with Bonferroni correction for multiple comparisons. Analyses were conducted using RStudio (v2024.04.2) and Microsoft^®^ Excel^®^ (v2409). PCA was based on scaled data and performed using the R package factoextra v1.0.7 [26]. Multivariate normality was assessed via Henze–Zirkler test using the *MVN* package [27] and rejected. Consequently, compositional differences between each plant-based subcategory and its animal-based counterpart were evaluated by permutational multivariate analysis of variance (PERMANOVA). Bonferroni correction was applied to account for multiple pairwise PERMANOVA comparisons, based on the number of product subcategories tested. Euclidean distance matrices were analysed with 9.999 permutations using the vegan package [28]. Bonferroni correction was applied to adjust *p*-values.

## 3. Results

In total, 298 PBMAs from 38 different brands were included in the analysis, comprising 182 meat analogues and 116 sausage analogues. As reference products, 101 meat products and 193 sausage products were collected.

### 3.1. Energy and Nutrient Content

Table 2 presents the means and standard deviations of energy and nutrient contents per 100 g for each product subcategory (graphical presentations of the respective comparisons can be found in Figures S1–S8).

- **Energy:** The highest mean energy density among PBMAs was found in salami analogues (1142 kJ/100 g or 273 kcal/100 g), while cooked sausage analogues had the lowest (628 kJ/100 g or 150 kcal/100 g). Compared to their animal-based counterparts, five out of nine PBMA categories had significantly lower (*p* < 0.005) energy content. The largest difference was found in the spreadable sausage category, where plant-based products contained on average 519 kJ or 124 kcal less per 100 g (224.8 ± 90.0 vs. 348.8 ± 74.8; *p* < 0.005).
- **Total Fat:** Fat content was significantly lower (*p* < 0.001) in the same five PBMA categories that also had reduced energy content. On average, animal-based sausage products contained 90% more fat than their plant-based alternatives.
- **Saturated Fat:** Saturated fat was lower (*p* < 0.05) in eight out of nine PBMA categories. Overall, animal-based products contained on average more than three times the amount of saturated fat compared to PBMAs. In cooked sausage, the average value for animal-based products was more than eight times higher (7.98 ± 2.63 g vs. 0.94 ± 0.36 g; *p* < 0.001).
- **Carbohydrates:** PBMAs contained more (*p* < 0.005) carbohydrates than meat products in all categories except for breaded products.
- **Sugar:** Sugar content was higher (*p* < 0.05) in seven out of nine PBMA categories. On average, plant-based products contained 66% more sugar than their animal-based counterparts.
- **Fibre:** Fibre content was higher (*p* ≤ 0.001) in all PBMA categories. While most meat products contained no fibre, PBMAs provided between 3.8 and 5.0 g per 100 g.
- **Protein:** Protein content varied widely across PBMAs. Salami and ham/bacon analogues had the highest average levels (20.9 g and 19.6 g/100 g, respectively) and did not differ significantly from their meat-based equivalents, although variability was considerable. In all other categories, PBMAs contained significantly less protein (*p* < 0.05), with the largest difference observed in cooked sausage (4.64 ± 2.34 g vs. 13.19 ± 1.63 g; *p* < 0.001).
- When considering the percentage of energy derived from protein, animal-based products generally scored higher. However, values were similar between plant-based and animal-based products in the minced meat and bratwurst categories, and salami analogues even exceeded their counterparts (31% vs. 26%).
- **Salt:** Salt content was significantly higher (*p* < 0.005) in three of four meat analogue categories, with the greatest difference in red meat products (1.8 g vs. 0.6 g/100 g; *p* < 0.001). No significant differences were found in four of five sausage subcategories, except for salami, where PBMAs contained about 40% less salt on average (*p* < 0.001). Animal-based salami products had the highest average salt content overall (3.8 g/100 g).
- Substantial variation was observed both within and between categories across all nutrients but fibre.

**Table 2:** Mean values and standard deviations of the energy and nutritional values per 100 g of products recorded for each sub-category (own calculations).

		Product Count		Energy (kJ)			Fat (g)			Saturated Fat (g)			Carbohydrates (g)			Sugar (g)			Dietary Fibre (g)			Protein (g)			Salt (g)		
	Category	PB	AB	PB	AB	*p*	PB	AB	*p*	PB	AB	*p*	PB	AB	*p*	PB	AB	*p*	PB	AB	*p*	PB	AB	*p*	PB	AB	*p*
**Meat**	Minced meat	52	30	205.83 ± 45.01	245.73 ± 39.59	0.004	12.41 ± 4.64	18.02 ± 4.46	<0.001	3.51 ± 3.35	7.53 ± 2.05	<0.001	6.4 ± 3.18	3.18 ± 3.84	0.004	1.5 ± 0.87	0.56 ± 0.60	<0.001	4.93 ± 2.10	0.16 ± 0.28	<0.001	14.64 ± 3.97	17.86 ± 3.47	<0.001	1.61 ± 0.47	1.18 ± 0.45	0.003
	Chicken	43	27	177.37 ± 63.56	148.67 ± 42.36	0.721	9.04 ± 6.66	6.94 ± 5.10	1	0.91 ± 0.66	2.06 ± 1.76	0.059	4.12 ± 2.21	1.00 ± 1.44	<0.001	0.94 ± 0.87	0.51 ± 0.62	0.134	4.89 ± 1.52	0.05 ± 0.12	<0.001	17.42 ± 5.46	20.56 ± 2.82	0.016	1.54 ± 0.45	1.24 ± 0.76	0.108
	Breaded meat	55	29	249.58 ± 40.53	229.66 ± 37.24	0.354	13.01 ± 3.73	10.8 ± 3.72	0.132	1.37 ± 0.66	2.83 ± 2.04	0.003	18.33 ± 3.81	16 ± 4.29	0.053	1.06 ± 0.64	0.9 ± 0.34	1	4.52 ± 1.50	0.77 ± 0.54	<0.001	12.49 ± 2.37	16.66 ± 2.99	<0.001	1.35 ± 0.31	1.07 ± 0.49	0.002
	Red meat	32	15	170.63 ± 60.82	155.27 ± 27.12	1	8.32 ± 6.21	7.37 ± 3.18	1	1.25 ± 1.65	2.81 ± 1.24	<0.001	5.73 ± 3.93	0.82 ± 0.81	<0.001	2.45 ± 2.41	0.48 ± 0.43	<0.001	4.9 ± 1.73	0.12 ± 0.16	<0.001	15.44 ± 5.41	21.28 ± 4.01	0.007	1.77 ± 0.47	0.86 ± 0.35	<0.001
**Sausage**	Bratwurst	22	26	196.05 ± 37.62	284.23 ± 42.79	<0.001	13.49 ± 3.64	24.66 ± 5.20	<0.001	2.3 ± 1.89	10.14 ± 2.33	<0.001	5.69 ± 2.51	0.59 ± 0.38	<0.001	1.38 ± 1.19	0.44 ± 0.30	0.032	3.75 ± 2.20	0.07 ± 0.19	<0.001	11.24 ± 6.37	15.27 ± 2.93	0.003	1.95 ± 0.49	1.97 ± 0.39	1
	Cooked sausage	47	48	150.43 ± 43.72	249.58 ± 46.07	<0.001	12.02 ± 4.33	21.55 ± 5.68	<0.001	0.94 ± 0.36	7.98 ± 2.63	<0.001	3.33 ± 1.47	0.85 ± 0.73	<0.001	1.27 ± 0.88	0.61 ± 0.61	0.001	5.06 ± 2.11	0.13 ± 0.20	<0.001	4.64 ± 2.34	13.19 ± 1.63	<0.001	2.13 ± 0.36	2.1 ± 0.27	1
	Salami	24	67	272.50 ± 81.01	378.27 ± 76.45	<0.001	15.98 ± 7.06	31.07 ± 7.48	<0.001	4.26 ± 4.48	12.64 ± 3.06	<0.001	9.33 ± 3.65	0.86 ± 0.82	<0.001	3.7 ± 1.79	0.72 ± 0.67	<0.001	4.15 ± 2.53	0.09 ± 0.21	<0.001	20.86 ± 11.24	23.99 ± 4.28	1	2.72 ± 0.54	3.77 ± 0.68	<0.001
	Ham/bacon	11	19	167.82 ± 84.19	202.63 ± 89.03	1	5.87 ± 5.70	12.88 ± 11.12	0.493	0.85 ± 0.58	5.15 ± 4.52	0.016	7.22 ± 3.83	0.65 ± 0.55	<0.001	3.21 ± 1.93	0.62 ± 0.54	0.001	3.88 ± 1.76	0.05 ± 0.16	0.001	19.64 ± 12.72	21.03 ± 5.19	1	2.6 ± 0.37	3.07 ± 1.50	1
	Spreadable sausage	12	33	224.83 ± 89.99	348.79 ± 74.79	0.002	18.38 ± 9.38	32.19 ± 9.22	<0.001	3.87 ± 4.67	12.91 ± 3.71	<0.001	7.26 ± 2.08	1.15 ± 0.80	<0.001	1.18 ± 0.79	0.81 ± 0.71	0.037	3.84 ± 2.14	0.28 ± 0.97	<0.001	5.67 ± 1.59	13.93 ± 2.41	<0.001	1.93 ± 0.38	2.08 ± 0.57	1

Considered significantly different, if *p* < 0.05. Analogues showed particularly high variability in these nutrients compared to the reference products, as indicated by the elongated, slightly right-leaning ellipse. Notably, PBMAs showed greater variability in nutrient profiles, as indicated by larger ellipse sizes in the PCA. PERMANOVA comparisons between each plant-based analogue subcategory and its animal-based reference counterpart revealed highly significant differences across all categories (*p* ≤ 0.0005).

### 3.2. Nutrient Profiling by Principal Component Analysis (PCA)

**Figure 1:**
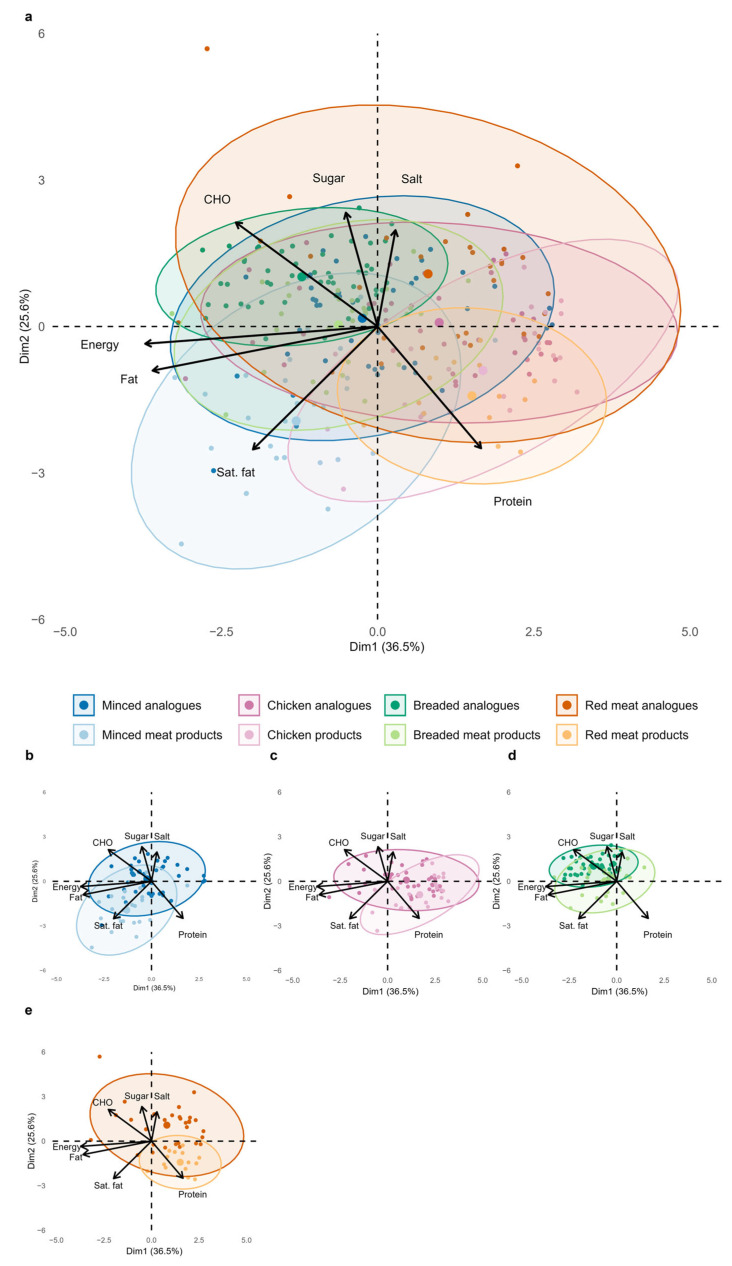
Principal component analysis (PCA) biplot of scaled nutrient profiles for plant-based meat products and their corresponding animal-based products (**a**), subdivided into subcategories (**b**–**e**). Each point represents a single product, coloured by type, with 95% confidence ellipses. CHO = carbohydrate; Sat. Fat = saturated fat. Small figures part (**b**–**e**) illustrate the categories extracted from the overall PCA: (**b**) Minced Meat (R^2^ = 0.2135, F = 21.72, *p* = 0.0004); (**c**) Chicken (R^2^ = 0.1258, F = 9.79, *p* = 0.0004); (**d**) Breaded Meat (R^2^ = 0.1331, F = 12.59, *p* = 0.0004); (**e**) Red Meat (R^2^ = 0.2091, F = 11.90, *p* = 0.0004). Group separation was assessed using PERMANOVA.

**Figure 2:**
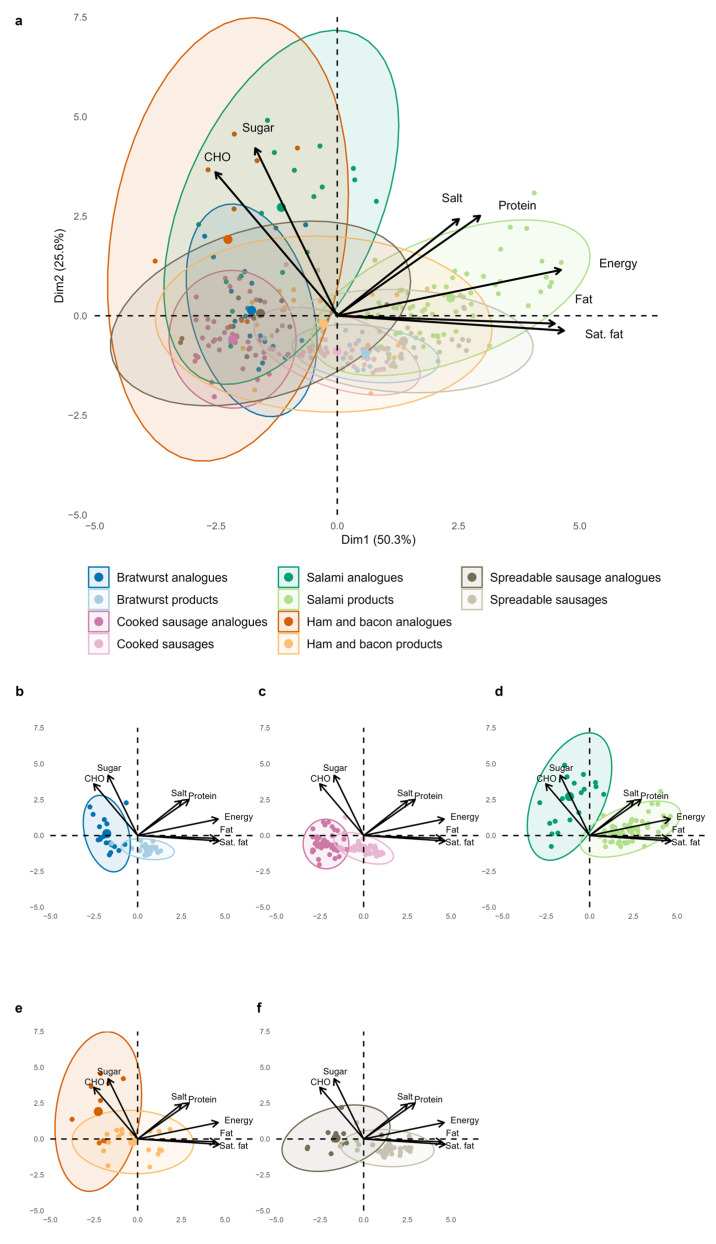
Principal component analysis (PCA) biplots of scaled nutrient profiles for plant-based sausage products and their corresponding animal-based products (**a**), subdivided into subcategories (**b**–**f**). Each point represents a single product, coloured by type, with 95% confidence ellipses. CHO = carbohydrate; Sat. Fat = saturated fat. Small figures part (**b**–**e**) illustrate the categories extracted from the overall PCA: (**b**) Bratwurst (R2 = 0. 43, F = 35.18, *p* = 0.0005); (**c**) Cooked sausages (R2 = 0.48, F = 84.78, *p* = 0.0005); (**d**) Ham and Bacon (R2 = 0.23, F = 8.59, *p* = 0.0005); (**e**) Salami (R2 = 0.43, F = 67.79, *p* = 0.0005); (**f**) Spreadable sausages (R2 = 0.40, F = 28.55, *p* = 0.0005). Group separation was assessed using PERMANOVA.

The PCA results for meat and sausage analogues compared to their reference products are presented in Figure 1a–d and Figure 2a–d. In the meat categories, the first two principal components explained 62.1% of the total variance in the product composition in the eight different categories. Energy and total fat contributed most to the first dimension (Dim1; 36.5%), while sugar and salt primarily shaped the second (Dim2; 25.6%). Considerable overlap was observed between plant-based and animal-based products, indicating a generally similar energy and nutrient composition. However, PBMAs tended to score higher in carbohydrates, sugar, and salt, whereas the clusters of meat products were located towards the negative range of the *x*-axis, reflecting their higher contents of saturated fat and protein. Ellipse sizes in the PCA biplots indicated a similar variability in nutrient composition between plant-based and animal-based products for minced meat, breaded, and chicken categories, whereas a notably greater variability was observed among plant-based red meat analogues.

In the sausage categories, the first two principal components explained 75.9% of the total variance (Dim1: 50.3%, Dim2: 25.6%). Fat, saturated fat, and energy contributed most to Dim1, while sugar and carbohydrates mainly influenced Dim2. Compared to the meat categories, the clusters of plant-based and animal-based sausage products overlapped less, indicating more distinct nutrient compositions, particularly in the bratwurst and salami categories. Plant-based sausage analogues were positioned more to the left and higher along the *y*-axis, reflecting higher carbohydrate and sugar contents, while meat products were characterized by higher fat, saturated fat, and energy contents. Also, Protein and salt contributed to a smaller extent to the separation. Plant-based salami analogues showed particularly high variability in these nutrients compared to the reference products, as indicated by the elongated, slightly right-leaning ellipse. Notably, PBMAs showed greater variability in nutrient profiles, as indicated by larger ellipse sizes in the PCA. PERMANOVA comparisons between each plant-based analogue subcategory and its animal-based reference counterpart revealed highly significant differences across all categories (*p* ≤ 0.0005).

### 3.3. Nutritional Quality Assessed by the Nutri-Score

**Figure 3:**
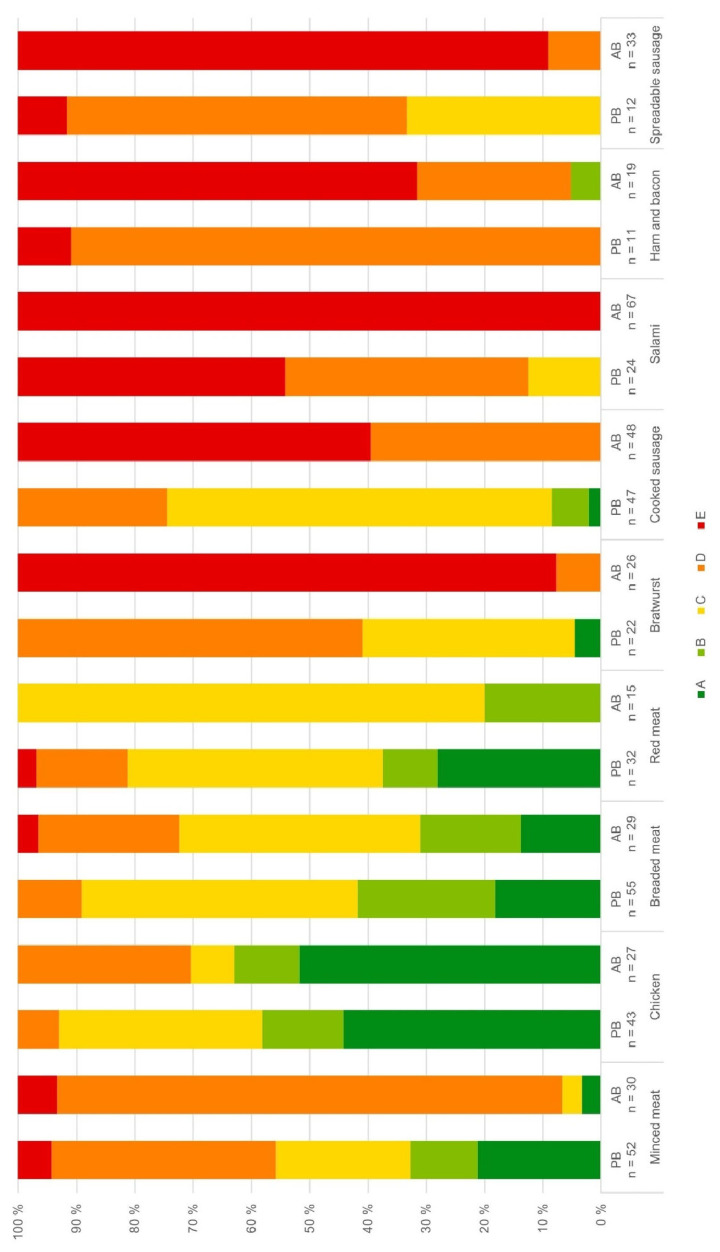
Comparison of Nutri-Score distributions for plant-based (PB) and animal-based (AB) products by subcategory. Nutri-Score A reflects highest, E lowest nutritional quality.

The Nutri-Score distributions of PBMAs and their animal-based reference products varied considerably across subcategories (Figure 3). Among plant-based meat analogues, 27% were rated A, 15% B, 37% C, 19% D, and 2% E. Higher proportions of favourable ratings (A or B) were observed in minced, breaded, and red meat analogues compared to their meat-based counterparts, although red meat analogues also showed greater variability. Overall, the chicken category exhibited the best Nutri-Score performance for both plant-based and animal-based products. In contrast, plant-based sausage analogues performed considerably worse: only 4% achieved an A or B rating, with the majority classified as C or D. Among animal-based sausages, however 84% were rated E. Across both groups, salami products showed the poorest Nutri-Score results, with 46% of plant-based and 100% of animal-based products rated E.

**Figure 4:**
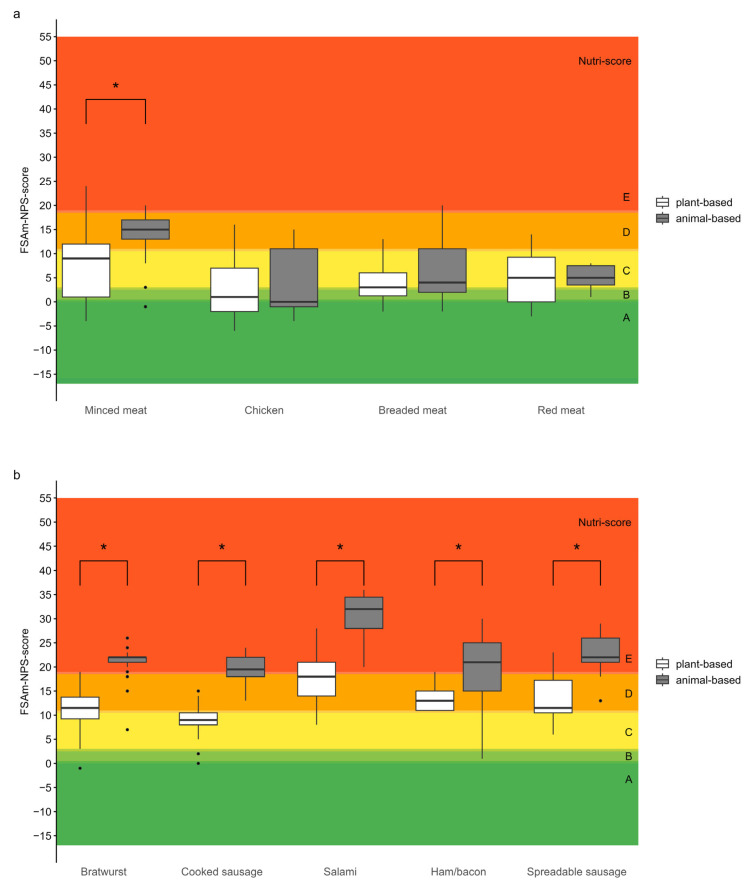
Boxplots depicting Nutri-Score distributions for plant-based and animal-based products by subcategory, derived from FSAm-NPS scores. Coloured background bands indicate Nutri-Score categories (dark green = A; light green = B; yellow = C; light orange = D; dark orange = E). Horizontal lines denote Nutri-Score thresholds, with letters A–E annotated to the right of each band. (**a**) Meat categories; (**b**) Sausage categories. Asterisks (*) indicate statistically significant differences (*p* < 0.05).

Figure 4a,b show the FSAm-NPS score distributions. PBMAs achieved significantly better scores than their animal-based counterparts in six out of nine categories (*p* < 0.01). While FSAm-NPS scores among meat categories were relatively consistent, significant differences were only observed in the minced meat category. In contrast, sausage analogues showed greater variability, with PBMAs consistently achieving lower (better) scores across all subcategories (*p* < 0.01).

### 3.4. Protein Source Distribution and Quality

The main protein sources used in the PBMAs derive from legumes, such as soy (36%) and pea (33%), but also wheat protein (26%), with minor contributions from sunflower (5%) and faba bean protein (1%) (see Figure S9). Protein combinations were common, particularly combinations of soy and wheat or wheat and pea proteins (see Table S1). A total of 39% of the PBMAs contained complementary protein combinations addressing different limiting amino acids or proteins without limiting amino acids, such as potato protein. Additionally, 22% of the products included soy protein, which is considered a high-quality protein source with a DIAAS of 91 [29]. Overall, 61% of the examined PBMAs were classified as having good protein quality based on either a high-quality protein source or a favourable combination of proteins (see Figure S10). Meat analogues showed a markedly higher proportion of products with good protein quality (72%) compared to sausage analogues (45%). The breaded and chicken analogue categories had the highest proportions of products with a good amino acid profile, at 85% and 84%, respectively.

### 3.5. Micronutrient Fortification

Only 37 PBMAs (12%) were fortified with one or more micronutrients: 37 with vitamin B12, 36 with iron, one with vitamin B2, and three with zinc. Declared vitamin B12 contents ranged from 0.38 to 1.3 µg per 100 g, covering approximately 10–33% of the daily requirement based on the German nutrient reference value (NRV) of 4.0 µg [30] (Table S2). In eleven products, iron was present but not listed as an added ingredient, suggesting naturally occurring levels; one additional product listed iron without specifying the amount. Declared iron contents ranged from 2.1 to 5.3 mg per 100 g, with salami analogues showing the highest values, covering nearly 40% of the daily requirement [30]. Other categories contributed around 20–30%. Three products were fortified with zinc, although only one declared the amount (1.5 mg per 100 g). Fortification was more common among meat analogues than sausage analogues. No product contained vitamin D or iodine.

### 3.6. Additives and Processing Characteristics

A total of 46 different additives were identified across the PBMAs, with methylcellulose being the most common, found in 175 products. Thickeners were the most common functional class, present in more than 60% of products, followed by acid regulators and colorants. Sausage analogues contained more additives than meat analogues, averaging 1.8–4.6 versus 1.4–2.7 additives per product depending on the category, respectively. Chicken and red meat analogues had the highest share of additive-free products, while no breaded analogue was free of additives. Flavourings were present in 92% of products, although the specific type was mostly not disclosed. A list of all additives and their distribution across subcategories can be found in Tables S3 and S4. Frequencies of additive functional classes can be found in Figure S11.

## 4. Discussion

To assess the nutritional quality of a product, it is useful to apply a nutrient profiling system that considers multiple nutrients rather than evaluating them individually. The Nutri-Score algorithm was applied in this study revealing that meat analogues generally exhibit more favourable nutrient profiles than sausage analogues. PBMAs achieved significantly better FSAm-NPS scores in six of nine subcategories. In all sausage categories and in the minced meat category, plant-based products scored significantly better than their meat-based counterparts, mainly due to lower saturated fat and higher fibre content. These findings support the common perception that plant-based alternatives are healthier in this regard [31].

Consistently, Pointke & Pawelzik (2022) reported significantly lower FSAm-NPS scores for plant-based alternatives in nine of twelve groups and Cutroneo et al., 2022 found more PBMAs in the favourable Nutri-Score categories A–C. Curtain & Grafenauer (2019), using the Australian Health Star Rating, also reported better scores for most PBMAs, except for minced meat. Alessandrini et al. (2021) found that most PBMAs were rated healthier than meat under the UK Nutrient Profiling Model [32,33,34,35]. Another UK study showed red meat analogues were rated significantly better than conventional red meat, mainly due to lower fat and higher fibre [36]. Only Katidi et al., 2023 found no significant differences in Nutri-Score of PBMAs and meat products [37]. However, they used simplified subcategorization (*n* = 4) for comparison and found no difference in energy content from their animal-based counterparts, while sausage imitations were higher in protein.

Studies on PBMAs in other countries revealed similar nutritional patterns as those identified in the present analysis [32,33,34,35,37]. Across studies, PBMAs generally show lower levels of total fat, saturated fat and energy, yet higher levels of fibre, carbohydrates and sugar compared to their animal-based counterparts.

The lower energy density of PBMAs, particularly evident in sausage analogues, is largely attributable to lower fat and higher fibre contents. Replacing processed meat products with plant-based alternatives may therefore contribute to a lower caloric intake and support weight management. This assumption is supported by a randomised crossover study by Crimarco et al. (2020), in which participants experienced significant weight loss during the phase in which they consumed plant-based meat alternatives instead of meat [38]. However, at this point it would also be interesting to systematically explore how PBMAs affect satiety and frequency of food intake and cravings compared to conventional products. For example, a recently published study showed no profound differences in plant-based meals compared to animal-based meals with respect to satiety, overall energy intake and mood [39].

It has been known for over 70 years that a high intake of saturated fats associates with an increased cardiovascular risk. Since then, moderate consumption of products with high levels of saturated fat has been recommended, most of which are dairy products, high-fat sausage and meat products and tropical oils [40]. In our study, the saturated fat content was consistently lower across all PBMA subcategories compared to their conventional counterparts, except for the chicken category. Unlike meat, PBMAs are also free from cholesterol, as are plant-derived foods. A systematic review and meta-analysis of controlled trials found that consumption of plant-based meat alternatives produces lower levels of total cholesterol, LDL cholesterol, and triglycerides compared to meat-based diets [41]. Some of these properties may stem from phytosterols which are present in soy for instance [42,43]. In line with that, the intake of soy-derived isoflavones was repeatedly linked with cholesterol-normalizing effects [44,45,46]. This suggests that replacing meat, particularly red and processed meats, with PBMAs could be an effective dietary strategy to reduce saturated fat intake and cardiovascular disease risk. We found that certain PBMAs still exhibited high saturated fat levels, most likely due to the frequent use of the tropical coconut oil. However, according to the ingredient lists in our analysis, rapeseed oil, which is rich in unsaturated oleic acid, linoleic acid and linolenic acid, but also vitamin E, flavonoids and carotenoids, is most frequently added. Based on these valuable ingredients, the consumption of rapeseed oil was associated with protective effects against diabetes, metabolic syndrome and a healthier lipid metabolism—health benefits which may possibly be supported by its use in PBMAs [47].

High intake of sugars and starch is associated with obesity, metabolic syndrome and even some types of cancer [48,49]. Replacing carbohydrate-free foods with foods containing sugars and starch might contribute to this unfavourable development. As for our investigative approach, carbohydrate and sugar contents were significantly higher in PBMAs compared to meat products, primarily due to the use of starch-based ingredients. Despite this, most PBMAs remained below the EU threshold for “low sugar” products (<5 g/100 g [50]). A modelling study from the UK found that replacing meat with plant-based alternatives led to a linear increase in carbohydrate intake, while sugar intake only rose slightly and became significant at full replacement levels [51]. Although sugar levels in PBMAs were generally moderate, Germany’s population already exceeds recommended daily limits of 50 g [52]. Given the high variability across products, reducing sugar content in PBMAs appears both feasible and advisable. Furthermore, we found here that PBMAs already contain significant amounts of fibre, with median values between 3.0 and 5.4 g/100 g. Fibre not only improves the technological properties of PBMA production but also certain health benefits, such as supporting gut microbiota, intestinal barrier function, and slowing the absorption of carbohydrates, saturated fats, and cholesterol, thereby contributing to weight control and prevention of non-communicable diseases [53,54,55]. Given that most Western diets are rather low in fibre and over 70% of German adults fail to meet the recommended daily intake of 30 g [30], PBMAs can contribute to improving population-level fibre intake. Depending on the product category, a 100 g portion may cover approximately 13 to 17% of the daily recommendation.

Although salt has important functions in food processing, including preservation, texture stabilization, and flavour enhancement [56], excessive intake is yet another risk factor for cardiovascular disease and increased mortality [57]. Meat analogues tended to have higher salt levels than their animal-based counterparts, whereas sausage analogues displayed similar levels and salami analogues even contained approximately 40% less salt than conventional salami. In Germany, average salt intake exceeds the recommended maximum of six grams per day, and current policy efforts focus primarily on conventional meat products [58]. In contrast, the UK has introduced specific salt reduction targets for plant-based meat products. However, only a minority meet these targets [35,59], similar to PBMAs in the present study. Therefore, with the currently high salt levels of meat analogues, these would be a poor alternative, especially for the elderly population with respect to an increased risk of cardiovascular disease. Then again, the observed salt range from 0.6 to 3.4 g per 100 g suggests that reformulation is possible and could support public health strategies. While several studies report that PBMAs often contain substantial amounts of salt and may exceed the levels in conventional meat products [32,35,60,61,62,63], lower salt contents, particularly in plant-based sausage alternatives, have also been reported [32,33,34,37,62,63,64,65]. Likewise, Gréa et al. (2023), who also analysed PBMAs in Germany as part of the National Reduction and Innovation Strategy, reported lower salt levels in salami analogues compared to conventional salami [66]. This may not reflect lower salt levels in the plant-based products, but rather comparatively high salt contents in the meat reference products used in their analysis. In the present study, salt levels of conventional meat products may be underestimated, as many values were obtained from the BLS rather than directly from products available in mainstream retail.

Protein is a key nutrient in meat, and PBMAs are often assumed to contain less, as we confirmed in the present analysis, with some exceptions which showed comparable levels. Noteworthy is that nearly half of the PBMAs were labelled with protein-related nutrition claims such as “high in protein”, according to EU regulations. However, in the EU, a “source of protein” claim can be made if at least 12% of the food’s energy value comes from protein, which can be perceived as relatively low standard in regards of meat alternatives [67]. Beyond that, protein contents varied considerably across individual products. Moreover, this is unlikely to be of concern from a public health perspective, as the general population in Germany consumes significantly more protein than recommended [68]. Nonetheless, findings in the literature are more heterogeneous. Several studies reported lower protein contents in PBMAs compared to conventional meat products [32,33,34,35,60,62,63,69,70], while others reported comparable levels [61,64,65,71,72] or even higher protein contents than in their meat-based counterparts [32,33,37,66]. Gréa et al. (2023) also reported three out of four PBMA categories to have higher protein contents than corresponding meat products, possibly attributed to differences in study design and product selection [66]. While the number of PBMAs available in mainstream retail has grown substantially by 2024, Gréa et al. included, such as niche products and products containing animal-derived ingredients like egg or dairy in their 2021 analysis.

We found the main protein sources in German PBMAs to be soy, wheat, and pea. Both, soy and wheat, are notably common allergens and, therefore, unsuitable for individuals with soy or gluten intolerance, wheat allergy, or celiac disease. Soy, with a DIAAS of 0.91, does not match the protein quality of meat but is still considered high quality. In contrast, pea and wheat proteins are of lower quality. For legumes, histidine and sulphur amino acids are limiting [73,74], whereas for cereals, lysine, threonine and tryptophane are specified as limiting [75]. Many products, however, contained combinations of protein sources that may compensate for limiting amino acids and thus improve overall quality. Based on DIAAS values and benefits of protein combinations, about 60% of the products were estimated to offer good protein quality, more often among meat analogues (72%) than sausage analogues (45%). Chicken and breaded categories showed particularly favourable results, with around 80% rated as having good quality. Zhang et al. (2024) confirmed that while meat contains more essential amino acids, soy-based analogues can reach similar protein quality [62]. De Marchi et al. (2021) found that most plant-based burgers represent high-protein quality, with the exception of being relatively low in methionine—a deficit which could be compensated through protein blending [76]. However, Zhang et al. (2024) also found that wheat-legume-based burger and pastrami analogues had significantly lower methionine and lysine levels than meat products [62]. Still, such combinations may provide adequate protein quality, as seen with soy, although this was not specifically confirmed in the study.

While average protein intake in many Western countries exceeds 150–200% of recommended levels [77], a moderate reduction in protein intake combined with increased fibre intake may contribute to a more balanced nutrient profile [62]. Still, for groups with higher needs—such as older adults, pregnant and breastfeeding women, or those with medical conditions—the substitution of meat with PBMAs might not be sufficient. In Canada, regulations already exist that define minimum requirements for protein content, protein quality, and micronutrients such as iron and vitamin B12 in PBMAs [78]. Similar regulations could be beneficial in Germany to ensure the nutritional adequacy of these products.

Although around 40% of products lacked high-protein quality, a varied plant-based diet over the course of the day can still provide all essential amino acids and support adequate protein utilization in healthy adults [79,80]. There is also evidence that plant protein processing, for example, by extrusion, can influence their function and nutritional value within the food product [81]. Nonetheless, further research on protein quality, absorption, and amino acid bioavailability in PBMAs is needed to minimize deficiency risks. In addition, studies on purine content and their potential effects on serum uric acid levels would be valuable to determine whether PBMAs represent a suitable alternative for individuals with hyperuricemia.

Meat is a key dietary source of important micronutrients such as zinc, iron, and vitamin B12. When replaced with plant-based alternatives, these nutrients must be considered -particularly vitamin B12, which is found exclusively in animal-derived foods [82].

As this study was based on label data, micronutrient levels were only assessed in fortified products. Only 12% of PBMAs were fortified with iron and vitamin B12; zinc was added to just three products, and vitamin B2 to one. The lack of fortification in most PBMAs is consistent with the findings of international studies [62,65,70,71]. Although data on micronutrients in PBMAs remain limited, several studies indicate that PBMAs may contain higher iron levels than meat [32,76]. Bryngelsson et al. (2022) reported that fortified Swedish PBMAs provide more iron and a comparable amount of vitamin B12 compared to meat references [65]. It must be taken into account that plant-derived ferric iron bioavailability is much lower compared to animal-derived heme iron [83]. Hence, fortification with more readily available ferrous iron could reinforce the role of PBMAs as a source of iron. In this context, novel products containing yeast-derived leghaemoglobin have been developed and may soon enter the German market, following EFSA’s 2024 classification of leghaemoglobin as safe for human consumption [84].

Unlike iron, zinc levels in PBMAs are generally lower than in meat products [32,70,76]. In addition, fibres may trap certain elements [85] and plant-derived anti-nutrients as, for instance, fibres and phytic acid may further impair the absorption of both, zinc and iron [86]. Processing methods such as extrusion could help break down phytic acid and improve mineral absorption [86].

Overall, modelling studies confirm that replacing meat with PBMAs may reduce intake of iron, zinc, and B12 unless products are fortified [51,87]. As PBMAs become more common in consumer diets, their micronutrient profile will play an increasingly important role. However, even with fortification, little is known about the actual bioavailability of these added nutrients in processed plant-based matrices. Ongoing research and regulatory efforts could help ensure nutritional adequacy and equivalence to conventional meat products at the national or EU level.

PBMAs are classified as UPFs according to the NOVA system [12]. Lately, frequent UPF consumption has been linked to unfavourable health effects, including weight gain and multiple chronical diseases and it is therefore recommended to limit their consumption to a minimum [12,13,88]. However, a randomized crossover study showed that PBMAs can promote slight weight loss and improved parameters of cardiovascular risk compared to meat [38], which may be related to a reduction in saturated fat intake and a slight though not significantly higher fibre intake with PBMAs.

A cohort study with over 260,000 participants found higher UPF consumption was associated with increased risks of cancer and cardiovascular disease [89]. This association was strongest for animal-based UPFs and soft drinks, whereas no such associations were found for PBMAs exhibiting lower energy density and more fibre. Toribio-Mateas et al. (2021) tested the hypothesis that industrial processing of plant ingredients does not automatically make a product a UPF [90]. They demonstrated that replacing five portions of meat per week with plant-based meat alternatives over four weeks elevates the beta diversity of the intestinal microbiome and improves butyrate metabolism. While health effects of PBMAs as UPFs remain to be elucidated [91], Messina et al. (2022) found no evidence that core criticisms of UPFs, such as low satiety or high energy density, apply to soy-based meat alternatives, arguing that the NOVA system oversimplifies food classification [92]. Alternative models like the SIGA Index [93] integrate effects of processing and formulation and may be useful in future research.

Another frequent criticism of PBMAs is the use of additives. Although additives are to be incorporated at concentrations that should not exceed the daily tolerable intake, as more highly processed foods are consumed, the intake and possible synergistic effects of these additives may accumulate. In this study, meat analogues averaged two additives per product and sausage analogues 3.3, respectively, less than the five found on average in Australia and New Zealand [94]. Some products contained eight or more, while 50 had none at all, indicating wide variability. Claims that PBMAs are loaded with chemicals, as often suggested in the media [95,96,97,98,99], are therefore unfounded. Thickeners were the most common additive group, present in around 60% of products and methylcellulose, found in 175 products, was the most frequently used. While some consumers seek “clean labels” [100], studies show that methylcellulose may also offer cholesterol- and blood sugar-lowering effects, similar to other dietary fibres [101,102,103]. Based on the scientific opinion released by the EFSA in 2010, such health benefits of methylcellulose can be expected with at least 5 g per day (in two or more servings) or, 4 g per meal, respectively [104]. Realistically, equal quantities can be achieved with the consumption of PBMAs produced with the cellulose derivative [105]. Other common additives included gums like konjac, xanthan, and carrageenan, have long been used in food processing and remain under review for their safety, although no serious health risks have been established to date [106]. Carrageenan has been re-evaluated by EFSA and deemed safe up to 75 mg/kg body weight per day [107]. Yet, current data supports association between long-term carrageenan-intake and chronic low-grade inflammation [108]. Unlike conventional processed meats, PBMAs are free from nitrites and nitrates. These are considered potentially harmful with respect to methemoglobinemia and nitrosamine formation, some of which are carcinogenic [109]. Although PBMAs can be highly processed, they should be seen as transitional products that help consumers reduce meat intake rather than replace whole foods [110]. This is particularly relevant since most PBMA consumers are not vegetarians but meat-eaters [111].

This study benefits from a comprehensive and systematically compiled dataset, covering almost 300 PBMAs from a broad range of manufacturers and product types. The analysis focused specifically on modern, highly processed PBMAs intended for the general population. In contrast to earlier studies, a clear distinction was made between industrially manufactured meat analogues based on protein isolates and more traditional plant-based products made from whole legumes or vegetables, which are typically sold in organic food stores and primarily target vegetarian consumers. This differentiation enhances comparability with conventional meat products, reflecting the current mainstream market.

Nevertheless, several limitations must be acknowledged. As data collection was primarily conducted in northern and central Germany, it cannot be ruled out that certain products or manufacturers were over- or underrepresented due to regional differences in retail availability and consumer behaviour. Moreover, while a few brands distribute PBMAs across multiple EU countries, most of the products included in this study are only available on the German market, which may limit the generalizability of the findings to other regions. Since data on meat reference products was often lacking ingredient lists, comprehensive comparison of additive use with PBMAs is limited. The detailed categorization, while advantageous for in-depth analysis, resulted in smaller subgroup sizes, which may limit statistical robustness and reduce comparability with studies employing broader classification schemes.

Categorization itself also posed challenges. For instance, products resembling chicken analogues were classified as red meat analogues if they were labelled with terms like “kebab style,” as kebab meat is, by definition, considered a red meat product. Some subcategories included only a limited number of products due to restricted market availability, which may have affected the clarity and comparability of results.

Furthermore, the evaluation in this study was based entirely on declared label data. While this approach allows for the assessment of a broad sample, it limits the accuracy of nutrient analysis, particularly regarding protein quality and micronutrient content. Protein quality was only estimated from the source listed using the data available from scientific literature. Fatty acid composition was not assessed; although fat sources were listed, their quantities were unknown, preventing evaluation of the ratio between monounsaturated and polyunsaturated fatty acids and related health implications. More accurate assessments of nutrient quality and digestibility would require laboratory analyses, which are time- and cost-intensive.

Finally, there were uncertainties in calculating Nutri-Scores, as fibre content declaration is voluntary (if not claimed as “high fibre” or “source of fibre”) [67], it was not always available and, in some cases, had to be estimated. Although this likely had only a minor impact on the results, some calculated Nutri-Scores differed from those declared on product packaging. A possible explanation is the revised Nutri-Score algorithm introduced on January 1, 2024, which companies may implement during a 24-month transition period [112].

## 5. Conclusions

In summary, PBMAs on the German market offer both, benefits and challenges.

Overall, the nutrient profile of PBMAs was found to be favorable. Yet, they often contain lower protein levels and quality, and more salt, limiting their substitutional value compared to animal-based products. As UPFs, they should be consumed in moderation and as part of a balanced diet.

Generally, the nutritional content varies widely across products. Therefore, consumers are obliged to study labels and choose options with high protein and low salt and fat, soy or legume-grain products are preferable. Occasional animal-product replacement is safe, but regular or complete substitution may risk nutrient deficiencies, given the current lack of regulation.

Improving PBMAs through fortification as well as reducing salt and sugar contents can enhance their nutritional value and environmental impact. While PBMAs support dietary shifts away from meat, more research, particularly human intervention studies, is required to assess their long-term health effects.

## Figures and Tables

**Table 1:** Product categorization and description.

Main Category	Subcategory	Examples
**Meat analogues**	Minced analogues	minced meat, burger patties, meatballs, cevapcici
	Chicken analogues	chicken-style strips, filets, chunks, skewers, cold cuts
	Breaded analogues	nuggets, schnitzels, cordon bleu
	Red meat analogues	steak, fillet, medallion, roast beef, kebab (döner), gyros
**Sausage analogues**	Bratwurst analogues	bratwurst, grilled sausage, currywurst
	Cooked sausage analogues	bologna, mortadella, lyoner, hot dogs, Leberkäse
	Salami analogues	salami, chorizo
	Ham/bacon analogues	cooked ham, ham cubes, bacon, rolled fillet of ham
	Spreadable sausage analogues	liverwurst, teewurst, onion mett

## Data Availability

The authors declare that the data supporting the findings of this study are available within the paper and its Supplementary Information Files. The data collected from food declarations and BLS (Bundeslebensmittelschlüssel) are available for download from the following link: https://www.foodsci.uni-kiel.de/de/Publikationen (accessed on 6 May 2024).

## Supplementary Materials

The following supporting information can be downloaded at: https://www.mdpi.com/article/10.3390/foods14213674/s1, Tables S1–S6 and Figures S1–S11: Table S1: Protein sources and combinations in meat and sausage analogues and their limiting amino acids. Table S2: Declared micronutrient content per 100 g and contribution to nutrient reference values (NRVs) in subcategories of plant-based meat analogues1. Table S3: List of food additives used in plant-based meat and sausage analogue products. Table S4: Distribution and frequency of food additives across subcategories of meat and sausage analogues. Table S5: Nutritional composition and ingredient list of all plant-based meat and sausage analogue products included in the analyses, grouped by subcategory. Table S6: Nutritional composition of all animal-based meat and sausage products included in the analyses, grouped by subcategory. Figure S1: Boxplots of energy content (kcal/100 g) for plant-based (PB) and animal-based (AB) products by subcategory. Figure S2: Boxplots of fat content (g/100 g) for plant-based (PB) and animal-based (AB) products by subcategory. Figure S3: Boxplots of saturated fat content (g/100 g) for plant-based (PB) and animal-based (AB) products by subcategory. Figure S4: Boxplots of carbohydrates content (g/100 g) for plant-based (PB) and animal-based (AB) products by subcategory. Figure S5: Boxplots of sugar content (g/100 g) for plant-based (PB) and animal-based (AB) products by subcategory. Figure S6: Boxplots of fibre content (g/100 g) for plant-based (PB) and animal-based (AB) products by subcategory. Figure S7: Boxplots of protein content (g/100 g) for plant-based (PB) and animal-based (AB) products by subcategory. Figure S8: Boxplots of salt content (g/100 g) for plant-based (PB) and animal-based (AB) products by subcategory. Figure S9: Distribution of main protein sources in plant-based meat and sausage analogues. Figure S10: Protein quality across subcategories of plant-based meat and sausage analogues. Figure S11: Frequency of additive functional classes in plant-based meat and sausage analogues (n = 298).

## Abbreviations

BLS	German Nutrient Database (Bundeslebensmittelschlüssel)
DGE	German Nutrition Society (Deutsche Gesellschaft für Ernährung)
DIAAS	Digestible Indispensable Amino Acid Scores
FSAm-NPS	Food Standards Agency modified nutrient profiling system
NRV	Nutrient reference value
PBMAs	Plant-based meat and sausage analogues
PCA	Principal Component Analysis
UPF	Ultra-processed food
