# Supplementary material for: A Nutritional Evaluation of Plant-Based Meat and Sausage Analogues
Source: Foods. 2025 Oct 28;14(21):3674. doi: 10.3390/foods14213674 (PMC12610220; doi:10.3390/foods14213674)
Supplement: Supplementary file 1 [file foods-14-03674-s001.zip › foods-3932751-supplementary.pdf]

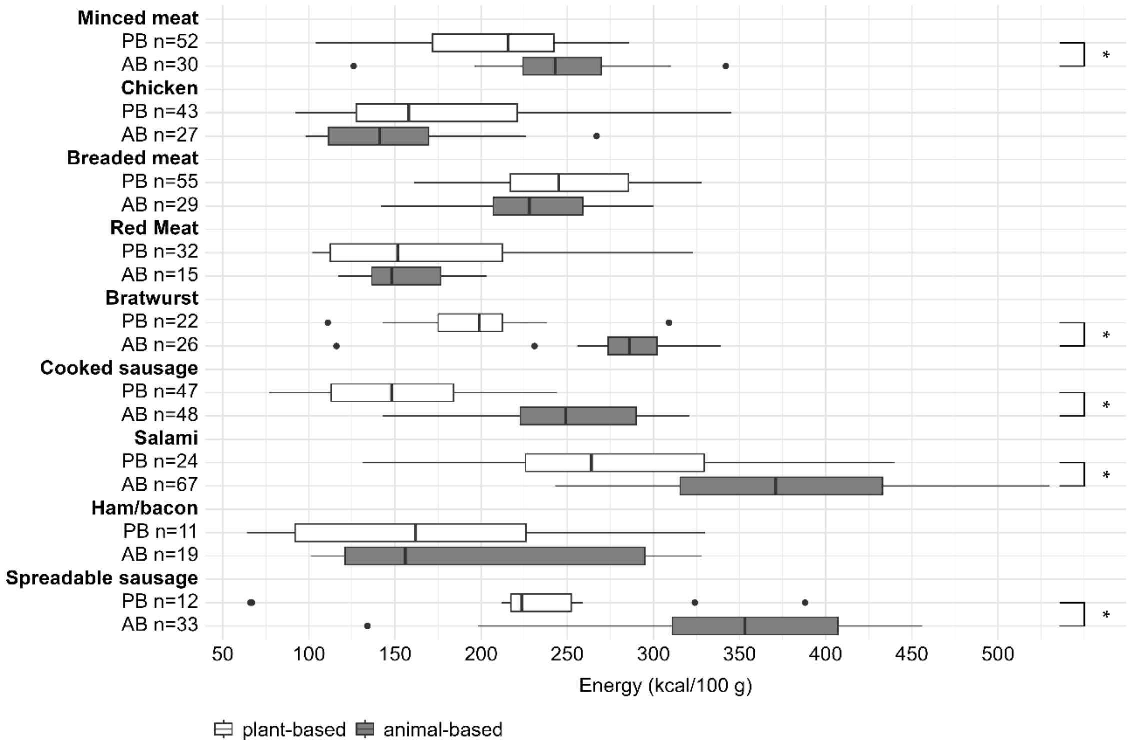

Figure S1: Boxplots of energy content (kcal/100 g) for plant-based (PB) and animal-based (AB) products by subcategory. Each boxplot represents the interquartile range (IQR; 25th–75th percentile), with the vertical line indicating the median. Whiskers extend to 1.5× IQR. Individual dots represent outliers. Asterisks (*) indicate statistically significant differences ($p < 0.05$).

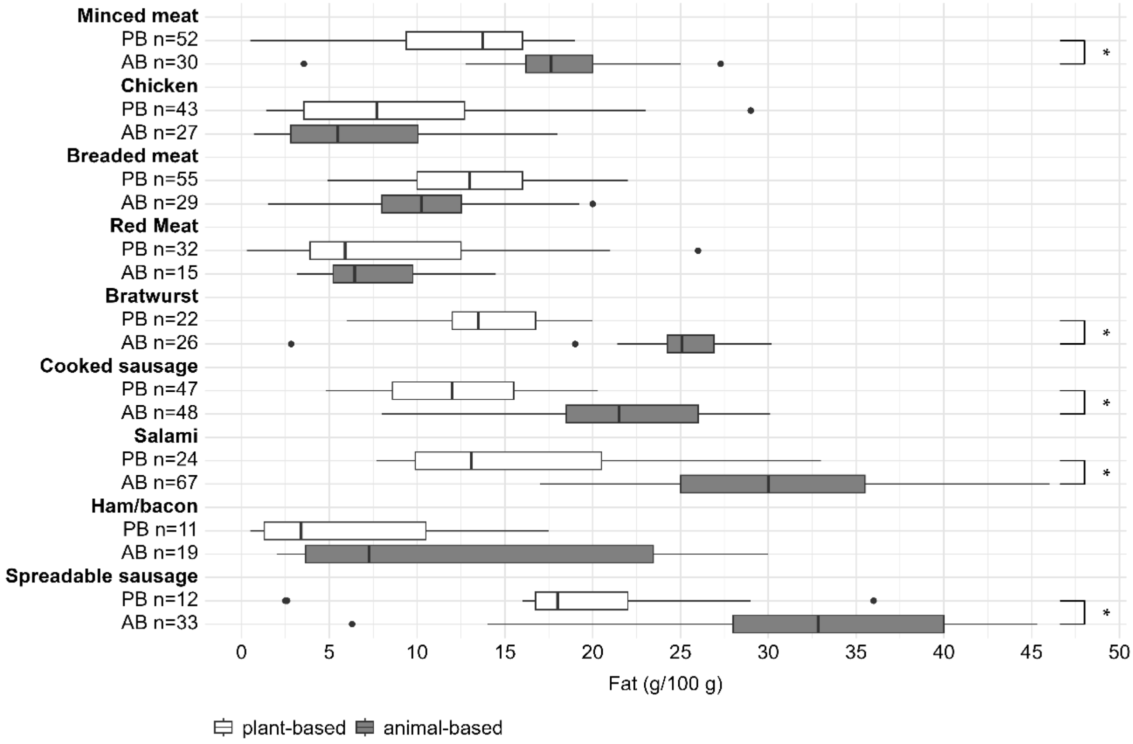

Figure S2: Boxplots of fat content (g/100 g) for plant-based (PB) and animal-based (AB) products by subcategory. Each boxplot represents the interquartile range (IQR; 25th–75th percentile), with the vertical line indicating the median. Whiskers extend to 1.5× IQR. Individual dots represent outliers. Asterisks (*) indicate statistically significant differences ($p < 0.05$).

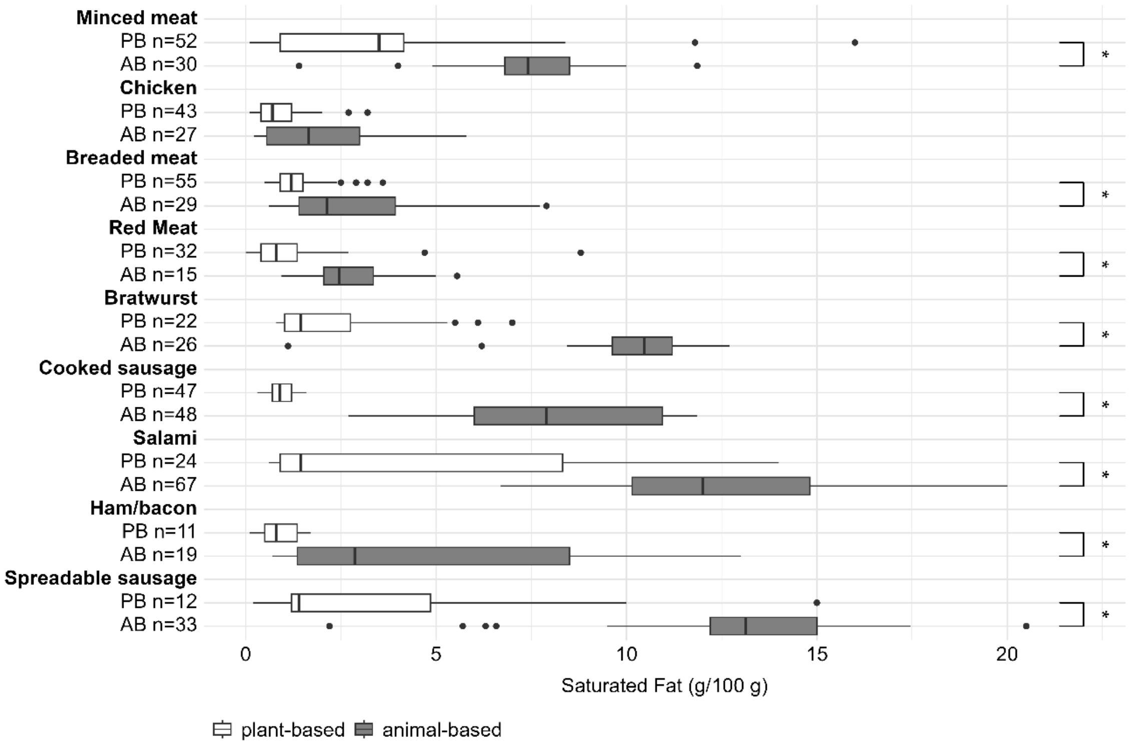

Figure S3: Boxplots of saturated fat content (g/100 g) for plant-based (PB) and animal-based (AB) products by subcategory. Each boxplot represents the interquartile range (IQR; 25th–75th percentile), with the vertical line indicating the median. Whiskers extend to 1.5× IQR. Individual dots represent outliers. Asterisks (*) indicate statistically significant differences ($p < 0.05$).

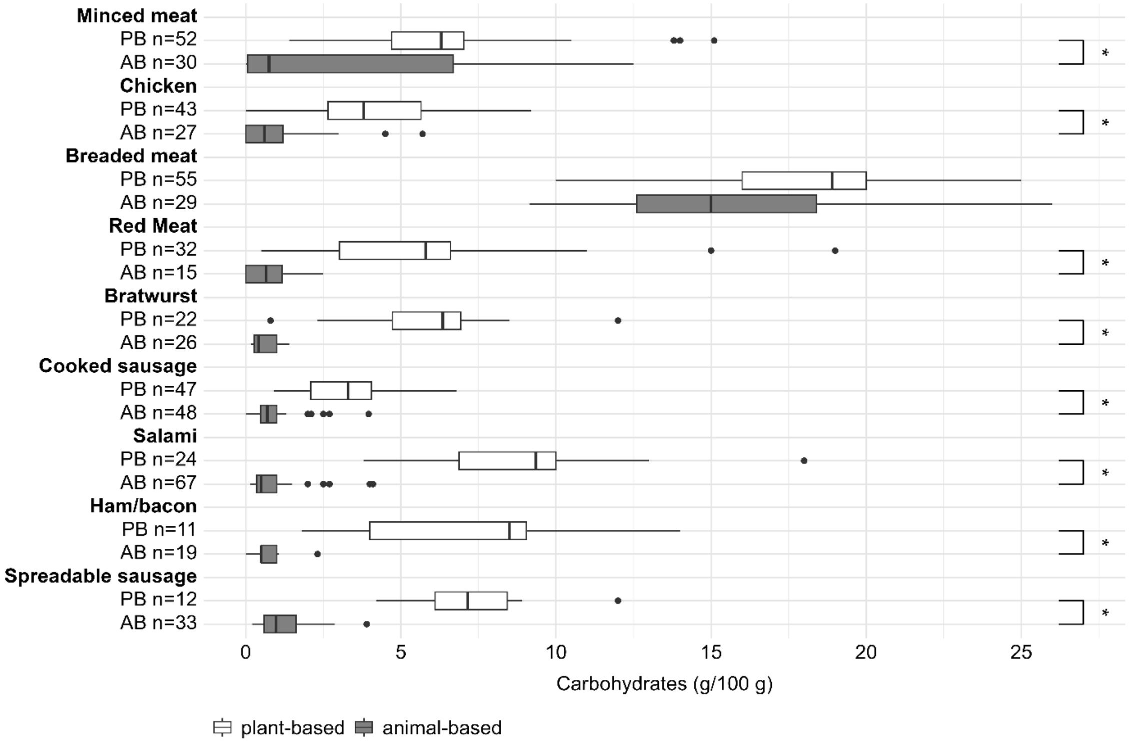

Figure S4: Boxplots of carbohydrates content (g/100 g) for plant-based (PB) and animal-based (AB) products by subcategory. Each boxplot represents the interquartile range (IQR; 25th–75th percentile), with the vertical line indicating the median. Whiskers extend to 1.5× IQR. Individual dots represent outliers. Asterisks (*) indicate statistically significant differences ($p < 0.05$).

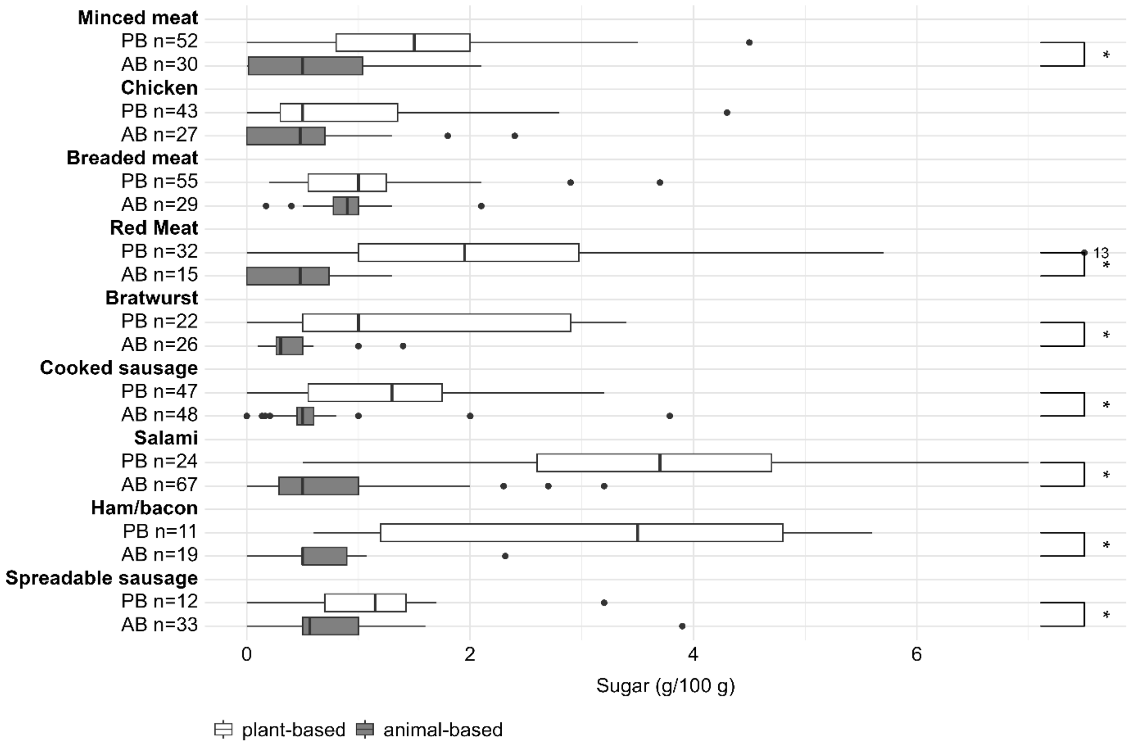

Figure S5: Boxplots of sugar content (g/100 g) for plant-based (PB) and animal-based (AB) products by subcategory. Each boxplot represents the interquartile range (IQR; 25th–75th percentile), with the vertical line indicating the median. Whiskers extend to 1.5× IQR. Individual dots represent outliers. Asterisks (*) indicate statistically significant differences ($p < 0.05$).

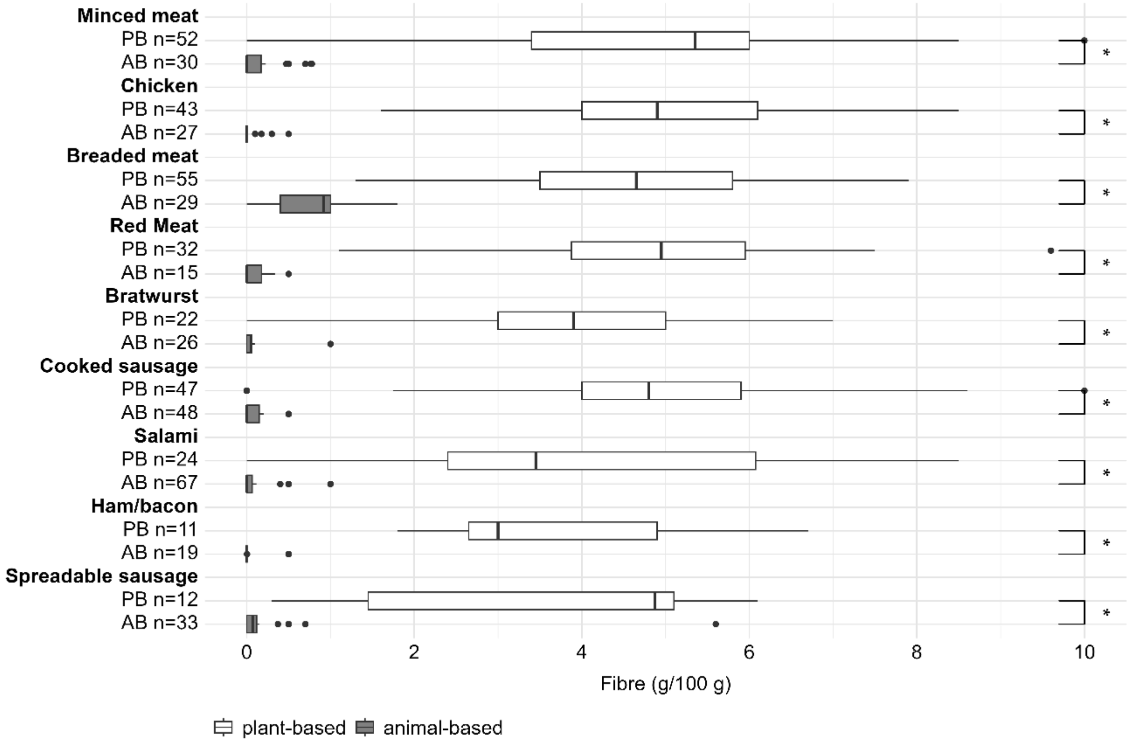

Figure S6: Boxplots of fibre content (g/100 g) for plant-based (PB) and animal-based (AB) products by subcategory. Each boxplot represents the interquartile range (IQR; 25th–75th percentile), with the vertical line indicating the median. Whiskers extend to 1.5× IQR. Individual dots represent outliers. Asterisks (*) indicate statistically significant differences ($p < 0.05$).

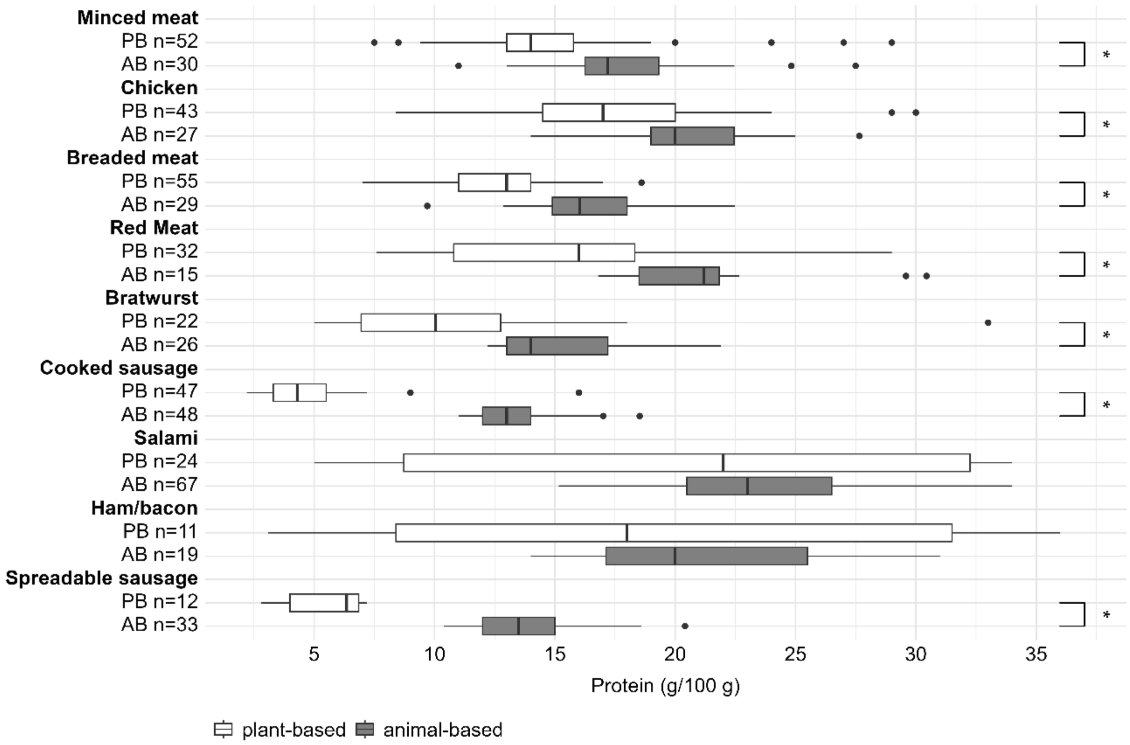

Figure S7: Boxplots of protein content (g/100 g) for plant-based (PB) and animal-based (AB) products by subcategory. Each boxplot represents the interquartile range (IQR; 25th–75th percentile), with the vertical line indicating the median. Whiskers extend to 1.5× IQR. Individual dots represent outliers. Asterisks (*) indicate statistically significant differences ($p < 0.05$).

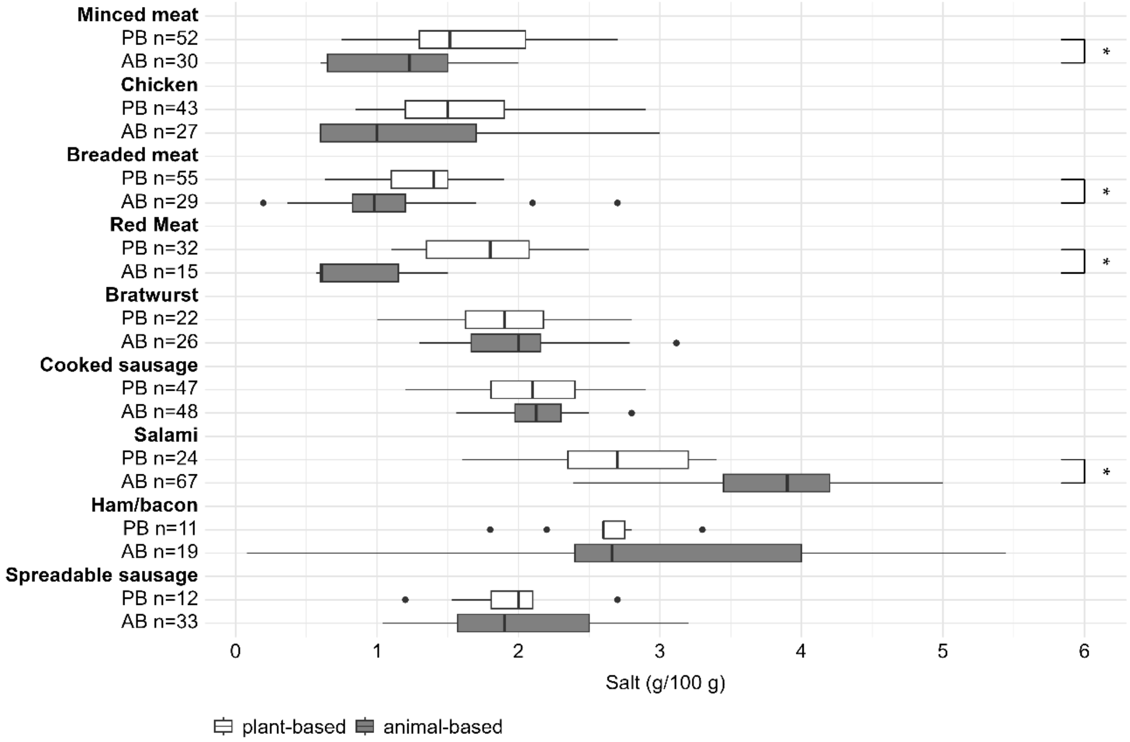

Figure S8: Boxplots of salt content (g/100 g) for plant-based (PB) and animal-based (AB) products by subcategory. Each boxplot represents the interquartile range (IQR; 25th–75th percentile), with the vertical line indicating the median. Whiskers extend to 1.5× IQR. Individual dots represent outliers. Asterisks (*) indicate statistically significant differences ($p < 0.05$).

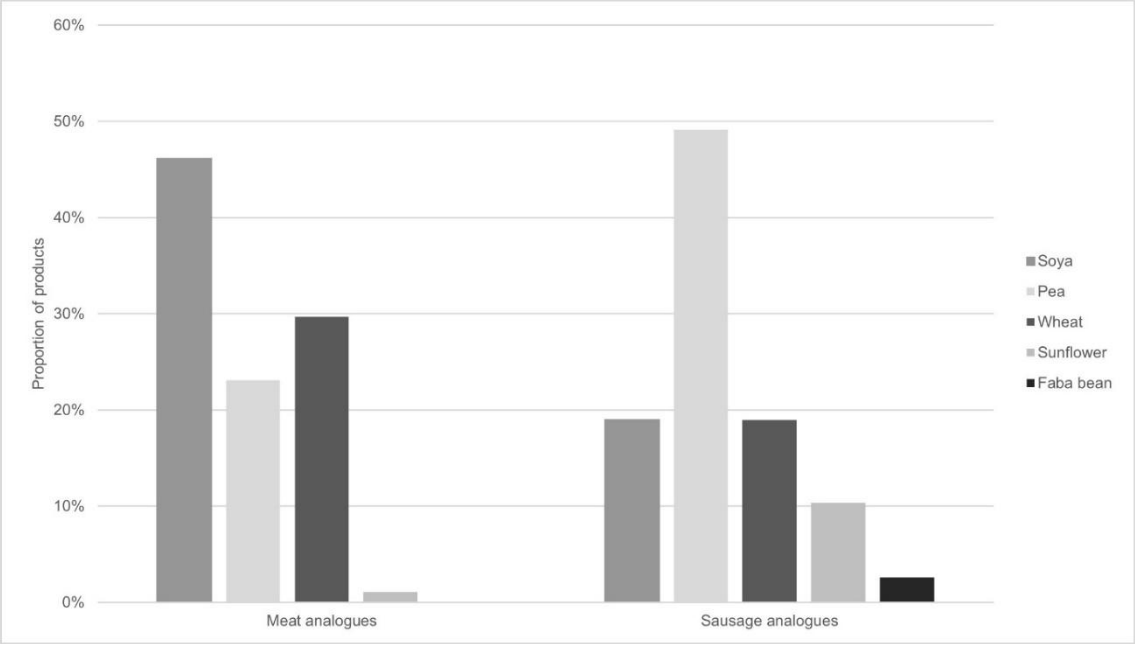

Figure S9: Distribution of main protein sources in plant-based meat and sausage analogues.

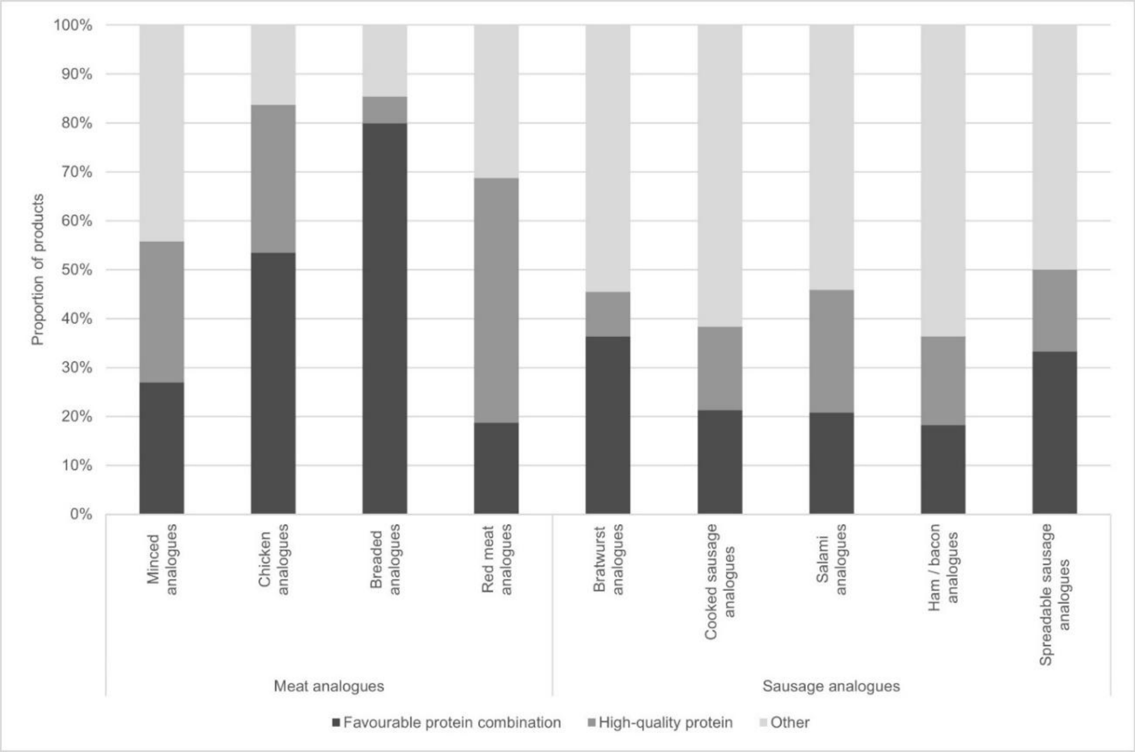

Figure S10: Protein quality across subcategories of plant-based meat and sausage analogues. Proportions of products classified by favourable protein combinations, high-quality proteins and products with a less balanced amino acid profile.

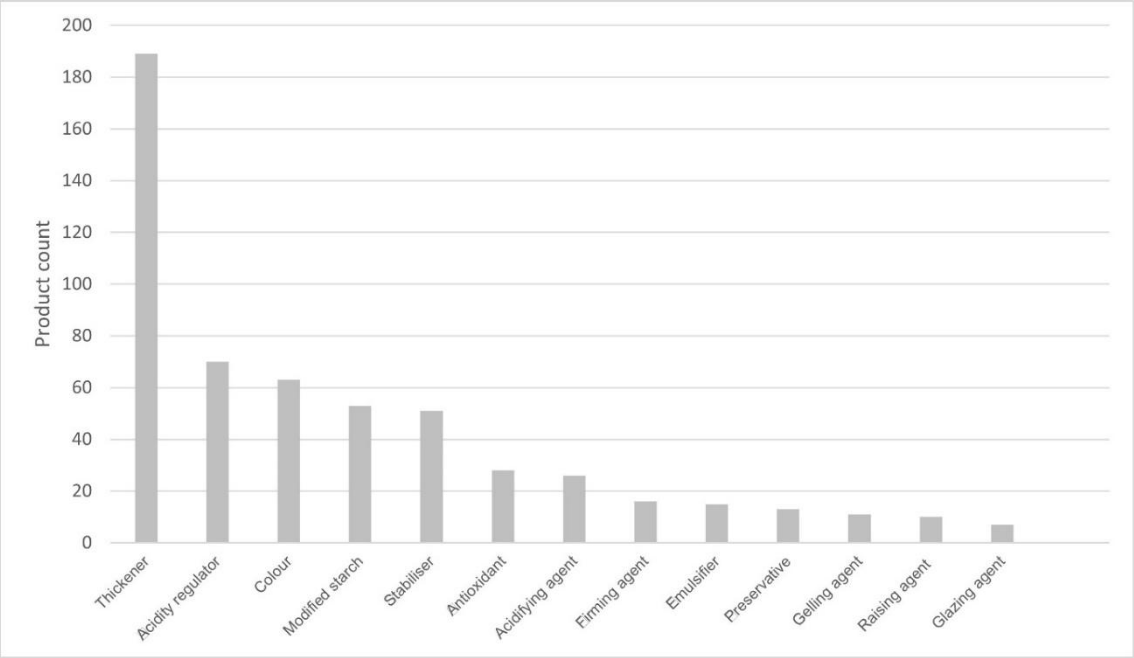

Figure S11: Frequency of additive functional classes in plant-based meat and sausage analogues (n = 298).

Table S1: Protein sources and combinations in meat and sausage analogues and their limiting amino acids.

Protein-quelle(n)	Fleisch-Imitate	Wurst-Imitate	Gesamt	Protein-quelle(n)	Fleisch-Imitate	Wurst-Imitate	Gesamt
Sy	44	18	62	Wh + Po	0	3	3
Pe	29	25	54	Pe + Sf	1	2	3
Sy + Wh	37	2	39	Pe + Ri	3	0	3
Pe + Fb	1	23	24	Pe + Sf + Fb	0	2	2
Wh	14	9	23	Wh + He	0	2	2
Wh + Pe	7	9	16	Wh + Fb	0	1	1
Wh + Fb + Pe	15	0	15	Wh + Po	0	1	1
Sy + Wh + Pe	9	0	9	Wh + Pe + Po	0	1	1
Sf	2	6	8	Wh + Le	1	0	1
Sf + Po	0	6	6	Pe + Ri + Fb	0	1	1
Sy + Pe	3	2	5	Wh + Sf	0	1	1
Pe + Po	0	4	4	SY + Sf + Po	1	0	1
Wh + Pe + Sf	4	0	4	Pe + Sf + Ot	1	0	1
Wh + Mu + Pe	4	0	4	Sy + Wh + Po	1	0	1
Fb	3	0	3				

Abbreviations: Sy = Soybean, Pe = Pea, Wh = Wheat, Fb = Faba bean, Sf = Sunflower, Po = Potato, Mu = Mushrooms, Ri = Rice, He = Hemp, Le = Lentil, Ot = Oats. Limiting amino acids are highlighted by colour: lysine in orange; methionine and cysteine in blue. Total number of products analysed: N = 298.

Table S2: Declared micronutrient content per 100 g and contribution to nutrient reference values (NRVs) in subcategories of plant-based meat analogues[1].

	Iron (mg)[2]	Vitamin B_{12} (µg)[3]	Zinc (mg)[4]	Vitamin B_2 (µg)[5]
**NRV[6]**	14 mg	4,0 µg	11 mg	1,2 mg
Minced meat analogues	2,75 (2,1-4,2)<br>20% (15%-30%)	1,1 (0,38-1,25)<br>28% (10%-33%)	1,5<br>14%	n.a.
Chicken analogues	2,5 (2,1-4,8)<br>18% (15%-34%)	1,00 (0,38-1,30)<br>25% (10%-33%)	n.a.	0,21<br>18%
Breaded analogues	3,4 (2,1-4,4)<br>24% (15%-31%)	0,54 (0,52-1,25)<br>14% (13%-31%)	n.a.	n.a.
Read meat analogues	2,7 (2,1-4,2)<br>19% (15%-30%)	1,12 (0,71-1,25)<br>28% (18%-31%)	n.a.	n.a.
Bratwurst analogues	3,2<br>23%	1,25<br>(31%)	n.a.	n.a.
Cooked sausage analogues	n.a.	n.a.	n.a.	n.a.
Salami analogues	5,3 (5,3-5,3)<br>38%	0,94 (0,94-0,94)<br>24%	n.a.	n.a.
Ham / bacon analogues	4,2<br>30%	0,71<br>18%	n.a.	n.a.
Spreadable sausage analogues	n.a.	n.a.	n.a.	n.a.

n.a.., not available.

[1] Data are reported as median (minimum–maximum) if the respective nutrient was declared in more than one product.
[2] Sample sizes for iron: minced meat (n = 4), chicken (n = 12), breaded (n = 8), red meat (n = 8), bratwurst (n = 1), raw sausage (n = 3), bacon/ham (n = 1)
[3] Sample sizes for vitamin B12 as above, except: minced meat (n = 3), bratwurst (n = 2).
[4] Sample size for zinc: n = 1.
[5] Sample size for vitamin B_2: n = 1.
[6] NRVs refer to recommended daily intakes for adult men and women (19–65 years) based on DGE (2024) values and moderate phytate intake.

Table S3: List of food additives used in plant-based meat and sausage analogue products. Number of products in each subcategory containing the respective food additive by E number and name.

E number	Additive name	Minced analogues (n = 52)	Chicken analogues (n = 43)	Breaded analogues (n = 55)	Red meat analogues (n = 32)	Bratwurst analogues (n = 33)	Cooked sausage analogues (n = 47)	Salami analogues (n = 24)	Ham / bacon analogues (n = 11)	Spreadable sausage analogues (n = 12)
E100	Curcumin			1						
E150d	Ammonium sulphite caramel							1		
E153	Vegetable carbon	1								
E160a	Carotenes			5		2	13	1	1	
E160b	Annatto bixin					1				
E160c	Paprika extract		1	3	2	1	5	3	1	1
E162	Betanin	3			1			3		
E163	Anthocyanins			2			6			
E170	Calcium carbonate		1	3						
E172	Iron oxides and hydroxides					3	7	5	1	3
E202	Potassium sorbate	2			1		1			
E260	Acetic acid						2			
E261	Potassium acetate	3	2	6	2				1	
E262	Sodium acetates	14	2	2	5	1	13	3	1	
E267	Buffered vinegar	3	4	2		1			1	
E270	Lactic acid		1	1	2	1		2	1	
E296	Malic acid		1		1					
E300	Ascorbic acid	1			2	1	14	2	1	2
E301	Sodium ascorbate							3		2
E322	Lecithin	1								1
E325	Sodium lactate						3			
E326	Potassium lactate						3			
E327	Calcium lactate					2	5	1		
E330	Citric acid	2	1	6	4	2	7			1
E333	Calcium citrate			1						
E334	Tartaric acid					1		1		
E392	Rosemary extract		1							
E401	Sodium alginate					6		8		
E404	Calcium alginate					2				
E406	Agar agar							1	1	
E407	Carrageenan	2	3	4	3	10	26	4	4	2
E407a	Processed Eucheuma seaweed	1	3	1		5	22	6		
E410	Locust bean gum			4	1		1	2	2	
E412	Guar gum		4	2	3	3		3		2
E415	Xanthan gum		4	4	7	2	11			
E417	Tara gum					1	6			
E425	Konjac		8	2	4	13	45	7	4	3
E450	Diphosphates			10		1		1		
E460	Cellulose	16	1	3	2			3		
E461	Methylcellulose	45	18	54	10	20	17	9	2	
E471	Mono- and diglycerides of fatty acids				1		1			1
E500	Sodium carbonates			7					2	
E508	Potassium chloride		2		1				2	
E509	Calcium chloride	2				4		8		
E516	Calcium sulphate	6	1							
E14XX	Modified starch		3	23		4	9	6	4	4

Table S4: Distribution and frequency of food additives across subcategories of meat and sausage analogues.

	Product count	Total additives[1]	Distinct additives[2]	Additives per product[3]
Minced meat analogues	52	102	15	2
Chicken analogues	43	61	19	1,4
Breaded analogues	55	146	22	2,7
Read meat analogues	32	52	18	1,6
Bratwurst analogues	22	87	23	4
Cooked sausage analogues	47	217	21	4,6
Salami analogues	24	83	23	3,5
Ham / bacon analogues	11	29	16	2,6
Spreadable sausage analogues	12	22	11	1,8

1 Total number of additives declared in all products within each subcategory (including multiple counts of the same additive).
2 Number of distinct additives declared in all products within each subcategory (no multiple counting), rounded.
3 Total additives / number of products in each subcategory.

Table S5: Nutritional composition and ingredient list of all plant-based meat and sausage analogue products included in the analyses, grouped by subcategory. CHO = carbohydrates; Sat. fat = saturated fat.

No.	Products	Brand	Source	Energy kcal/100 g) (kJ/100 g)	Fat (g/100 g)	Sat. fat (g/100 g)	CHO (g/100 g)	Sugar (g/100 g)	Fibre (g/100 g)	Protein (g/100 g)	Salt (g/100 g)	FSAm-NPS	Nutri Score	Ingredients
**Plant-based minced meat products**														
**1**	Beyond Burger	Beyond Meat	Rewe	250 (1,047)	19.0	5.6	3.5	0.0	1.3	17,0	0.8	11	D	Water, 15% pea protein isolate, rapeseed oil, flavouring, rice protein, coconut oil, dried yeast, preservative: potassium lactate; vinegar, stabiliser: methylcellulose, calcium chloride; potato starch, salt, apple extract, colour: beetroot red; pomegranate concentrate, potassium salt
**2**	Beyond Meatballs	Beyond Meat	Rewe	247 (1,035)	18.0	5.2	7.2	0.1	0.0	16,0	1.0	12	D	Water, pea protein (14%), canola oil, coconut oil, flavouring, rice protein, dried yeast, stabiliser (methylcellulose), potato starch, salt, potassium salt, spices, herbs in variable proportion 0.5% (parsley, rosemary, sage, basil & oregano), apple extract, garlic powder, maize vinegar, concentrated lemon juice, onion powder, pomegranate extract, emulsifier (sunflower lecithin), colour (beetroot), maltodextrin, carrot powder.
**3**	Beyond Hack	Beyond Meat	Rewe	196 (820)	12.0	2.9	5.6	0.6	1.2	16,0	0.8	1	B	Water, 15% pea protein, rapeseed oil, flavouring, rice protein, coconut oil, dried yeast, preservative: potassium lactate; vinegar, stabiliser: methylcellulose, calcium chloride; potato starch, salt, apple extract, colour: beetroot red; pomegranate concentrate, potassium salt.
**4**	Veganer Grill-Taler mit Kräuter-Marinade	Billie Green	Rewe	286 (1,198)	14.0	8.0	10.5	4.5	1.0	29,0	2.7	24	E	25% wheat gluten, drinking water, textured vegetable protein (wheat protein, pea protein), coconut fat, lemon juice from concentrated lemon juice, rapeseed oil, fully hydrogenated rapeseed oil, table salt, garlic, vegetables: onion, carrots; spices, flavouring, colouring foods: paprika concentrate, carrot concentrate; wheat starch, sugar, herbs, dextrose, yeast extract, dried tomatoes, jalapeño.
**5**	Vegan Burger	Block House	Kaufland	194 (810)	13.0	1.1	3.5	3.5	10.0	9,4	1.8	7	C	Vegetable stock (contains carrots, celery, leek, onions), sunflower protein (13%), mushrooms, onions, rapeseed oil, shiitake mushrooms, soy sauce (water, soybeans, wheat, table salt), dried tomatoes (dried tomatoes, table salt), defatted sunflower seeds (3%), citrus fibre, colouring food (beetroot), dried glucose syrup, herbs, spices, iodised table salt (table salt, potassium iodate), garlic, sugar, thickeners (methylcellulose, cellulose).
**6**	Veganes Hack	endori	Rewe	219 (917)	10.0	0.9	3.9	0.5	3.0	27,0	1.3	1	B	Water, 20% pea protein, 13% faba bean concentrate, rapeseed oil, maize fibre, spirit vinegar (AT: alcohol vinegar), malt extract (water, gluten-free roasted barley malt, hops, yeast), apple cider vinegar, tomato juice concentrate, table salt, spices, dextrose, potato fibre, psyllium husk, herbs, acid: citric acid; antioxidant: ascorbic acid; natural flavourings.
**7**	Veganer Burger	endori	Rewe	233 (975)	14.0	5.9	6.0	2.0	5.1	19,0	1.5	12	D	Water, rapeseed oil, diced onions, 6% pea protein, 6% textured wheat protein (wheat protein, wheat flour), 5% wheat protein, coconut fat, 4% faba bean concentrate, wheat straw fibre, spirit vinegar (AT: alcohol vinegar), thickener: methylcellulose; table salt, spices, rice flour, malt extract (water, roasted barley malt, hops, yeast), psyllium husk, caramelised sugar syrup, balsamic vinegar powder, smoked maltodextrin (maltodextrin, smoke), smoked table salt (table salt, smoke), caramel sugar syrup, natural flavourings.
**8**	Vegane Mini-Frikadellen	Food For Future	Penny	245 (1,026)	16.0	3.5	7.0	1.5	7.5	15,0	2.2	11	D	Water, onions, textured wheat protein 9% (wheat protein 95%, wheat starch, firming agent: calcium sulphate), rapeseed oil, fully hydrogenated coconut fat, textured pea protein 3% (pea protein 51%, pea extract), sunflower protein, pea flour 3%, thickeners (methylcellulose, cellulose), pea protein 2%, table salt, citrus fibre, maize grits, parsley, natural flavourings, potato starch, spices, herbs, spice extract, acidity regulators (potassium lactate, sodium acetates), maltodextrin.
**9**	Sensationa l Burger	Garden Gourmet	Rewe	195 (817)	13.5	3.9	1.9	1.0	5.9	14,0	0.9	1	B	Drinking water, 20.4% soy protein, vegetable oils (rapeseed, coconut), acidity regulator (buffered vinegar), flavourings, stabiliser (methylcellulose), maize

No.	Products	Brand	Source	Energy kcal/100 g) (kJ/100 g)	Fat (g/100 g)	Sat. fat (g/100 g)	CHO (g/100 g)	Sugar (g/100 g)	Fibre (g/100 g)	Protein (g/100 g)	Salt (g/100 g)	FSAm-NPS	Nutri Score	Ingredients
														starch, fruit and vegetable concentrates (apple, beetroot, carrot, hibiscus), table salt, barley malt extract, soy sauce powder (soybeans, wheat, salt).
**10**	Sensationa l Hack / Faschierte s	Garden Gourmet	Edeka	177 (741)	10.4	3.9	2.0	1.2	6.9	15,7	0.8	-1	A	Drinking water, 23.5% soy protein, vegetable oils (rapeseed, coconut), acidity regulator (buffered vinegar), stabiliser (methylcellulose), flavourings, fruit and vegetable concentrates (beetroot, apple, carrot, hibiscus), table salt, barley malt extract, spices (garlic, onions, black pepper), soy sauce powder (soybeans, wheat, salt).
**11**	„Hackbällc hen“ Vegan	Green Cuisine	Edeka	201 (840)	13.0	1.1	4.9	0.8	6.8	13,0	1.1	-1	A	Rehydrated pea protein (66%), rapeseed oil, onion, pea flour, bamboo fibre, stabiliser: methylcellulose, natural flavouring, onion powder, barley malt extract, tomato paste, psyllium husk fibre, garlic powder, salt, citrus fibre, basil, black pepper, sage, pea fibre, whole oat flour, starch, spices, tomato powder, mushroom powder.
**12**	Vegane ~~Fleisch~~ Mini-Frikadellen	Green Legend	Edeka	275 (1,150)	19.0	1.8	14.0	2.5	2.5	11,0	1.1	5	C	Drinking water, rapeseed oil, pea protein (10%), onions, chopped sunflower seeds, spices, natural flavourings, stabiliser: methylcellulose; table salt, spice extract, mustard (drinking water, mustard seeds, spirit vinegar, table salt, spices), sugar, parsley, colouring food: beetroot powder.
**13**	Plant-Based Hack	Green Mountain	Edeka	180 (753)	9.2	0.8	7.1	1.9	3.1	15,0	1.7	3	C	Water, textured pea protein 17%, rapeseed oil, pea flour, fermented onion extract, wheat gluten, thickener: methylcellulose, natural flavouring, pea protein 1.5%, spirit vinegar, colouring beetroot juice concentrate, wheat flour, barley malt extract, iron, vitamin B12.
**14**	Vegane Cevapcici	Greenforce	Rewe	232 (972)	16.0	4.0	6.5	1.5	6.0	13,0	2.2	12	D	Drinking water, 13% textured pea protein (pea protein, pea extract), paprika, rapeseed oil, spices, partially defatted sunflower seeds, onions, fully hydrogenated coconut fat, thickeners: methylcellulose, cellulose; 3% pea flour, 2% pea protein, table salt, potato starch, flavourings, smoke, acidity regulators: potassium lactate, sodium acetates; maltodextrin.
**15**	Vegane Mini-Frika	Greenforce	Rewe	232 (972)	16.0	4.0	6.5	1.5	6.0	13,0	2.2	12	D	Drinking water, 13% textured pea protein (pea protein, pea extract), onions, rapeseed oil, partially defatted sunflower seeds, fully hydrogenated coconut fat, thickeners: methylcellulose, cellulose; 3% pea flour, 2% pea protein, table salt, maize grits, parsley, flavourings, potato starch, spices, herbs, spice extract, acidity regulators: potassium lactate, sodium acetates; maltodextrin.
**16**	Vegane Köttbullar	Greenforce	Rewe	234 (980)	16.0	4.0	7.0	1.5	6.0	13,0	2.2	12	D	Drinking water, 13% textured pea protein (pea protein, pea extract), onions, rapeseed oil, partially defatted sunflower seeds, fully hydrogenated coconut fat, thickeners: methylcellulose, cellulose; 3% pea flour, maize grits, 2% pea protein, table salt, spices, flavourings, potato starch, caramel powder (caramelised sugar syrup, maltodextrin), acidity regulators: potassium lactate, sodium acetates; maltodextrin.
**17**	Veganes Hack	Greenforce	Rewe	139 (580)	5.4	0.9	5.4	2.4	5.4	14,0	1.4	-1	A	93% soaked sunflower protein (water, 33% sunflower protein), spices, sunflower oil, salt, natural mushroom flavour, caramel, yeast extract, spirit vinegar.
**18**	Like Hack	Like Meat	Rewe	143 (598)	2.7	0.4	7.5	2.5	5.5	20,0	1.4	-3	A	Water, textured soy protein 35%, soy protein concentrate 2%, sunflower oil, sea salt, flavouring, caramelised sugar, pepper.
**19**	Like Frikadellen	Like Meat	Edeka	155 (648)	8.7	4.6	4.8	2.3	8.5	10,0	1.5	7	C	Water, textured soy protein 14%, onions, gluten-free oat hull fibre, sunflower oil, coconut fat, spices, colouring foods (beetroot powder, caramelised carrot concentrate, carrot concentrate), starch, table salt, citrus fibre, natural flavouring, dextrose, thickeners (methylcellulose, carrageenan), colour (vegetable carbon).
**20**	The Wonder Veganes Hack	My Vay	Aldi Nord	170 (710)	9.0	8.0	5.5	1.0	3.5	15,0	1.3	14	D	Drinking water, 10% textured soy protein concentrate, 9% coconut oil, 8% textured soy protein isolate, acidity regulators: potassium lactate, sodium acetates; natural flavourings, 3% textured maize starch, thickener: methylcellulose; dextrose, table salt, beetroot extract, natural paprika flavouring, psyllium husk, inulin, preservative: potassium sorbate.

No.	Products	Brand	Source	Energy kcal/100 g) (kJ/100 g)	Fat (g/100 g)	Sat. fat (g/100 g)	CHO (g/100 g)	Sugar (g/100 g)	Fibre (g/100 g)	Protein (g/100 g)	Salt (g/100 g)	FSAm-NPS	Nutri Score	Ingredients
**21**	The Wonder Vegane Cevapcici	My Vay	Aldi Nord	172 (718)	9.4	8.4	5.2	0.8	3.1	15,0	1.7	17	D	Drinking water, 9% textured soy protein concentrate, 8% coconut oil, 8% textured soy protein isolate, acidity regulators: potassium lactate, sodium acetates; natural flavourings, 3% textured maize starch, thickener: methylcellulose; table salt, dextrose, psyllium husk, inulin, beetroot extract, herbs, natural paprika flavouring, pepper extract, preservative: potassium sorbate.
**22**	Vegane Frikadellen Paprika	My Vay	Aldi Nord	213 (893)	12.2	1.0	13.8	2.1	2.2	11,1	1.2	4	C	Drinking water, 17% textured wheat protein (75% wheat protein, wheat flour), 16% rapeseed oil, 9% paprika, wheat flour, toasted onions, mustard (drinking water, mustard seeds, spirit vinegar, table salt, spices, spice extract), natural flavouring, thickener: methylcellulose; spices, iodised table salt (table salt, potassium iodate), sugar, tomato paste concentrate, caramel (caramelised sugar syrup, maltodextrin), herbs, dextrose, yeast.
**23**	Vegane Frikadellen Klassik	My Vay	Aldi Nord	233 (974)	13.1	1.0	15.1	1.9	2.7	12,4	1.8	5	C	Drinking water, 18% textured wheat protein (75% wheat protein, wheat flour), 17% rapeseed oil, wheat flour, toasted onions, mustard (drinking water, mustard seeds, spirit vinegar, table salt, spices, spice extract), natural flavouring, thickener: methylcellulose; herbs, spices, iodised table salt (table salt, potassium iodate), caramel (caramelised sugar syrup, maltodextrin), dextrose, yeast.
**24**	Vegane Frikadellen Petersilie	My Vay	Aldi Nord	250 (1,048)	15.2	1.2	13.8	1.3	1.6	14,0	1.9	13	D	Drinking water, 18% textured wheat protein (75% wheat protein, wheat flour), 17% rapeseed oil, wheat flour, toasted onions, mustard (drinking water, mustard seeds, spirit vinegar, table salt, spices, spice extract), natural flavouring, thickener: methylcellulose; herbs, spices, iodised table salt (table salt, potassium iodate), caramel (caramelised sugar syrup, maltodextrin), dextrose, yeast.
**25**	Vegane Frikadellen Petersilie	My Vay	Aldi Süd	215 (898)	14.0	4.0	6.5	1.7	6.0	13,0	2.2	12	D	Drinking water, 12% textured pea protein (pea protein, pea extract), onions, rapeseed oil, partially defatted sunflower seeds, fully hydrogenated coconut fat, thickeners: methylcellulose, cellulose; 3% pea flour, 2% pea protein, table salt, maize grits, 1% parsley, flavourings, potato starch, spices (nutmeg, onion), marjoram, spice extract, acidity regulators: potassium lactate, sodium acetates; maltodextrin.
**26**	Vegane Frikadellen Paprika	My Vay	Aldi Süd	215 (898)	14.0	4.0	6.5	1.5	6.0	13,0	2.2	12	D	Drinking water, 12% textured pea protein (pea protein, pea extract), 7% paprika, rapeseed oil, spices (chilli, garlic, cumin, paprika, pepper, onion), partially defatted sunflower seeds, onions, fully hydrogenated coconut fat, thickeners: methylcellulose, cellulose; 3% pea flour, 2% pea protein, table salt, potato starch, flavourings, oak wood smoke, acidity regulators: potassium lactate, sodium acetates; maltodextrin.
**27**	Vegane Frikadellen Klassik	My Vay	Aldi Süd	217 (910)	14.0	4.0	7.0	1.5	6.5	13,0	2.2	11	D	Drinking water, 12% textured pea protein (pea protein, pea extract), onions, rapeseed oil, partially defatted sunflower seeds, fully hydrogenated coconut fat, thickeners: methylcellulose, cellulose; 3% pea flour, maize grits, 2% pea protein, table salt, spices (garlic, pepper, allspice, onion), flavourings, potato starch, colouring food: caramel powder (caramelised sugar syrup, maltodextrin), acidity regulators: potassium lactate, sodium acetates; maltodextrin.
**28**	Vegane Burger	My Veggie	Edeka	174 (729)	10.2	0.7	4.7	2.3	5.6	13,3	1.3	0	A	Water, 22% textured pea protein, mushrooms, rapeseed oil, spices, thickener: methylcellulose; table salt, colouring food: beetroot juice concentrate, carob powder; dextrose, pea protein, onion extract, smoked dextrose (dextrose, smoke), tomato powder, herbs.
**29**	Vegane Frikadellen	My Veggie	Edeka	244 (1,020)	15.9	1.5	10.4	1.5	4.9	12,7	1.6	9	C	Water, 18% textured soy protein, 6.4% rapeseed oil, onions, toasted onions, potato flakes, table salt, mustard (water, mustard seed, spirit vinegar, sugar, table salt, spices), spices, thickener: methylcellulose; acidity regulator: potassium lactate; dextrose, herbs.
**30**	Vegane Frikadellen	My Veggie	Edeka	244 (1,020)	15.9	1.5	10.4	1.5	4.9	12,7	1.6	9	C	Water, 17% textured soy protein, rapeseed oil, onions, toasted onions, potato flakes, table salt, mustard (water, mustard seed, spirit vinegar, sugar, table salt,

No.	Products	Brand	Source	Energy kcal/100 g) (kJ/100 g)	Fat (g/100 g)	Sat. fat (g/100 g)	CHO (g/100 g)	Sugar (g/100 g)	Fibre (g/100 g)	Protein (g/100 g)	Salt (g/100 g)	FSAm-NPS	Nutri Score	Ingredients
														spices), spices, thickener: methylcellulose; acidity regulator: potassium lactate; herbs, dextrose.
31	Veganes Hack	pure vood	Penny	249 (1,043)	18.0	11.8	6.3	2.0	3.5	14,2	2.0	21	E	Water, 13.5% pea protein, coconut fat, pea extract, rapeseed oil, flavours, natural flavour, thickeners (methylcellulose, cellulose), acidity regulators (potassium lactate, potassium acetate), table salt, spirit vinegar, colouring foodstuff (beetroot powder), maltodextrin, caramel syrup, spices, smoke flavouring.
32	Veganes Hack	Rewe Beste Wahl	Rewe	249 (1,043)	18.0	11.8	6.3	2.0	3.5	14,2	2.0	21	E	Water, 13.5% pea protein, coconut fat, pea extract, rapeseed oil, flavours, natural flavour, thickeners (methylcellulose, cellulose), acidity regulators (potassium lactate, potassium acetate), table salt, spirit vinegar, colouring foodstuff (beetroot powder), maltodextrin, caramel syrup, spices, smoke flavouring.
33	Vegane Burger Patties	Rewe Beste Wahl	Rewe	132 (553)	8.1	0.7	4.7	2.3	5.7	7,5	1.3	1	B	Rehydrated pea protein 45% (water, pea protein concentrate), water, mushrooms, rapeseed oil, table salt, thickener (methylcellulose), onions, colouring foodstuff (beetroot juice concentrate), pea protein 0.8%, dextrose, onion extract, carob powder, spices, smoke.
34	Mini-Frikadellen	Rewe Beste Wahl	Rewe	245 (1,026)	16.0	3.5	7.0	1.5	7.5	15,0	2.2	11	D	Water, onions, textured WHEAT PROTEIN 9% (WHEAT PROTEIN 95%, wheat starch, firming agent (calcium sulphate)), rapeseed oil, coconut fat (fully hydrogenated), textured pea protein 3% (pea protein 51%, pea extract), sunflower protein, pea flour 3%, thickeners (methylcellulose, cellulose), pea protein 2%, table salt, citrus fibre, maize granules, parsley, natural flavours, potato starch, spices, herbs, spice extract, acidity regulators (potassium lactate, sodium acetates), maltodextrin.
35	Vegane Burger	Rewe Beste Wahl	Rewe	144 (602)	6.3	0.8	4.6	0.4	6.1	14,2	1.5	-1	A	Water, 15% pea protein, pea extract, rapeseed oil, flavours, natural flavour, thickeners (methylcellulose, cellulose), acidity regulators (potassium lactate, potassium acetate), spirit vinegar, colouring foodstuff (beetroot powder), maltodextrin, caramel syrup, table salt, spices, smoke flavouring.
36	Vegane Mühlen Frikadellen Minis	Rügenwalder Mühle	Rewe	167 (698)	7.9	0.6	8.1	1.5	7.6	12,0	1.8	1	B	Drinking water, onions, 13% SOY PROTEIN, rapeseed oil, flavour, starch, bamboo fibres, thickener: methylcellulose, processed Eucheuma seaweed; sunflower protein, potato protein, citrus fibres, spices, table salt, spirit vinegar, caramel, glucose.
37	Veganes Mühlen Hack	Rügenwalder Mühle	Rewe	105 (441)	1.5	0.1	2.0	0.5	5.7	18,0	1.8	-1	A	Drinking water, SOY PROTEIN, rapeseed oil, acidity regulator: buffered vinegar, table salt, natural flavour, spirit vinegar, caramel.
38	Veganes Mühlen Hack	Rügenwalder Mühle	Rewe	155 (648)	9.3	5.2	1.4	0.5	5.2	14,0	1.1	9	C	Drinking water, 20% SOY PROTEIN, coconut oil, rapeseed oil, thickener: methylcellulose, spirit vinegar, starch, flavour, table salt, colouring foodstuffs: concentrates of radish, beetroot, paprika, carrot; protective culture.
39	Vegane Mühlen Frikadellen	Rügenwalder Mühle	Rewe	170 (712)	9.0	0.7	10.0	0.7	8.0	8,5	1.7	2	B	Drinking water, 11% SOY PROTEIN, rapeseed oil, onions, WHEAT FLOUR, starch, OAT FIBRES, table salt, spices, spice extracts, spirit vinegar, natural flavour, thickener: methylcellulose, colouring foodstuffs: concentrates of beetroot, blueberry, carrot, caramelised carrot; yeast, sugar, maltodextrin.
40	Vegane Mühlen Hackröllchen	Rügenwalder Mühle	Rewe	156 (651)	9.4	5.3	1.5	0.5	5.0	14,0	1.4	10	C	Drinking water, 17% SOY PROTEIN CONCENTRATE, coconut oil, rapeseed oil, thickener: methylcellulose, 1.8% SOY PROTEIN ISOLATE, starch, spirit vinegar, table salt, spices, flavour, colouring foodstuffs: concentrates of radish, beetroot, paprika, carrot; protective culture.

No.	Products	Brand	Source	Energy kcal/100 g) (kJ/100 g)	Fat (g/100 g)	Sat. fat (g/100 g)	CHO (g/100 g)	Sugar (g/100 g)	Fibre (g/100 g)	Protein (g/100 g)	Salt (g/100 g)	FSAm-NPS	Nutri Score	Ingredients
**41**	Vegane Hackbällchen	take it veggie	Kaufland	231 (965)	16.0	1.5	5.0	0.5	2.0	16,0	1.5	4	C	Water, plant protein extrudate (14% pea protein, 2.5% chickpea powder), rapeseed oil, thickener: methylcellulose; onions, 1.4% pea protein, iodised table salt (table salt, potassium iodate), spices, potato starch, citrus fibres, natural flavour (contains barley), yeast extract, colouring foodstuff: barley malt extract.
**42**	Vegane Burger-Scheiben	take it veggie	Kaufland	214 (896)	14.0	1.0	5.0	0.5	2.0	16,0	1.5	3	C	Water, plant protein extrudate (14% pea protein, 2.5% chickpea powder), rapeseed oil, thickener: methylcellulose; onions, 1.4% pea protein, iodised table salt (table salt, potassium iodate), spices, potato starch, citrus fibres, natural flavour (contains barley), yeast extract, colouring foodstuff: barley malt extract.
**43**	Hick-Hack-Hurra	The Vegetarian Butcher	Rewe	126 (529)	0.5	0.1	2.9	2.0	6.4	24,0	1.3	-4	A	91% soy base (drinking water, SOY PROTEIN, BARLEY MALT EXTRACT), natural flavours, spirit vinegar, dextrose, table salt, yeast extract, spices (pepper, garlic, onions, ginger, coriander, mace), caramelised sugar, iron diphosphate, vitamin B12.
**44**	Pflanzliche Frikadellen	vegan leben	Famila	212 (886)	16.0	8.1	4.9	1.6	5.2	10,0	1.3	14	D	Water, plant oils (coconut, sunflower), 13% soy protein, onions, 3% wheat protein, thickeners: methylcellulose, carrageenan; spices, wheat straw fibre, natural flavour, wheat flour, salt, yeast extract, acidity regulator: citric acid; barley malt extract, spirit vinegar, herbs, iron, zinc, vitamin B12.
**45**	gehacktes	Vegini	Rewe	272 (1,139)	18.0	16.0	5.7	1.5	5.3	20,0	1.5	17	D	Drinking water, pea protein, coconut oil, onion, pea fibre, stabiliser: methylcellulose, spirit vinegar, beetroot juice concentrate, potato starch, spices, apple juice concentrate, caramel syrup, sunflower oil, spice extract, salt.
**46**	Vegane Burger Patties	Vehappy	Netto	174 (729)	10.2	0.7	4.7	2.3	5.6	13,3	1.3	0	A	Water, 21% textured pea protein, mushrooms, rapeseed oil, spices, thickener: methylcellulose; table salt, colouring foodstuff: beetroot juice concentrate, carob powder; dextrose, pea protein, onion extract, smoked dextrose (dextrose, smoke), tomato powder, herbs.
**47**	vegane Cevapcici	Vemondo	Lidl	236 (989)	16.0	4.0	7.0	3.0	5.0	14,0	2.2	13	D	Drinking water, 10% textured wheat protein (wheat protein, wheat starch, firming agent: calcium sulphate), paprika, rapeseed oil, spices, onions, fully hydrogenated coconut fat, 3% textured pea protein (pea protein, pea extract), 3% sunflower protein, thickeners: methylcellulose, cellulose; 3% pea flour, 2% pea protein, table salt, potato starch, flavours, oak smoke, acidity regulators: potassium lactate, sodium acetates; maltodextrin.
**48**	vegane Snack Frikadellen	Vemondo	Lidl	240 (1,006)	16.0	3.5	7.0	1.5	5.0	15,0	2.2	14	D	Drinking water, onions, 9% textured wheat protein (wheat protein, wheat starch, firming agent: calcium sulphate), rapeseed oil, fully hydrogenated coconut fat, 3% textured pea protein (pea protein, pea extract), 3% sunflower protein, 3% pea flour, thickeners: methylcellulose, cellulose; 2% pea protein, table salt, maize granules, parsley, natural flavours, potato starch, spices, herbs, spice extract, acidity regulators: potassium lactate, sodium acetates; maltodextrin.
**49**	veganes Hack	Vemondo	Lidl	104 (437)	1.5	0.2	2.1	0.4	5.0	18,0	1.3	-2	A	Drinking water, 28% textured soy protein, citrus fibre, table salt, rapeseed oil, caramel syrup, maltodextrin, spice extracts, tomato juice concentrate, colouring radish concentrate, mushroom juice concentrate.

No.	Products	Brand	Source	Energy kcal/100 g) (kJ/100 g)	Fat (g/100 g)	Sat. fat (g/100 g)	CHO (g/100 g)	Sugar (g/100 g)	Fibre (g/100 g)	Protein (g/100 g)	Salt (g/100 g)	FSAm-NPS	Nutri Score	Ingredients
**50**	vegane Cavapcici	Vemondo	Lidl	232 (972)	16.0	4.0	6.5	1.5	6.0	13,0	2.2	12	D	Drinking water, 10% textured pea protein (pea protein, pea extract), paprika, rapeseed oil, partially defatted sunflower seeds, onions, fully hydrogenated coconut fat, thickeners: methylcellulose, cellulose; 3% pea flour, table salt, 2% textured wheat protein (wheat protein, wheat starch, firming agent: calcium sulphate), 2% pea protein, potato starch, spices, flavours, oak smoke, maltodextrin, acidity regulators: potassium lactate, sodium acetates.
**51**	vegane Snack Frikadellen	Vemondo	Lidl	232 (972)	16.0	4.0	6.5	1.5	6.0	13,0	2.2	12	D	Drinking water, 10% textured pea protein (pea protein, pea extract), onions, rapeseed oil, partially defatted sunflower seeds, onions, fully hydrogenated coconut fat, thickeners: methylcellulose, cellulose; 3% pea flour, table salt, 2% textured wheat protein (wheat protein, wheat starch, firming agent: calcium sulphate), 2% pea protein, parsley, potato starch, spices, herbs, maize granules, flavours, spice extracts, maltodextrin, acidity regulators: potassium lactate, sodium acetate.
**52**	Vegane Frikadellen	Garden Gourmet	Markant	213 (892)	12.6	0.9	10.0	1.1	6.5	12,0	1.0	-2	A	Drinking water, 15.2% soy protein, variable amounts of vegetable oils (rapeseed, sunflower), onions, breadcrumb (wheat flour, table salt, yeast, rapeseed oil, paprika extract), spirit vinegar, maize starch, herbs (1.3% parsley, marjoram), potato flakes, soy sauce (soybeans, wheat, salt), table salt, flavours, spices (garlic powder, white pepper), yeast extract, stabiliser (methylcellulose).
**Plant-based Chicken Products**														
**53**	Veganes Chicken BBQ	endori	Rewe	222 (930)	7.8	0.7	6.1	1.8	4.3	30,0	1.2	-2	A	Water, 21% wheat gluten, 19% mushroom protein, 6% pea protein, rapeseed oil, maize fibre, spices, spirit vinegar, potato starch, table salt, apple cider vinegar, dextrose, sugar, garlic purée, potato fibre, garlic, smoked table salt (table salt, smoke), psyllium husks, thickener: guar gum; natural flavours.
**54**	Veganes Chicken Natur	endori	Rewe	221 (924)	7.9	0.7	5.6	1.0	4.7	29,0	1.3	-1	A	Water, 21% wheat gluten, 19% mushroom protein, rapeseed oil, 6% pea protein, maize fibre, spirit vinegar, potato starch, table salt, apple cider vinegar, dextrose, spices, garlic purée, potato fibre, psyllium husks, thickener: guar gum; natural flavours.
**55**	Veganes Chicken Kebab	endori	Edeka	205 (857)	6.0	0.6	6.1	1.2	3.7	29,0	1.2	-1	A	Water, 21% wheat gluten, 19% mushroom protein, 6% pea protein, rapeseed oil, spirit vinegar, maize fibre, spices, potato starch, apple cider vinegar, garlic, table salt, dextrose, tomato juice concentrate, herbs, potato fibre, garlic purée, psyllium husks, natural flavours.
**56**	Veganes Chicken Chili Paprika	endori	Edeka	220 (920)	7.7	0.8	5.7	1.0	4.5	29,0	1.2	-2	A	Water, 21% wheat gluten, 19% mushroom protein, rapeseed oil, 6% pea protein, smoked paprika powder (paprika, smoke), chilli, spices, spirit vinegar (Austria: spirit vinegar), maize fibre, potato starch, table salt, apple cider vinegar, dextrose, garlic purée, potato fibre, smoked table salt (table salt, smoke), psyllium husks, sugar, thickener: guar gum; natural flavours.
**57**	Vegane Chunks Klassik	Food For Future	Penny	132 (554)	3.4	0.4	2.5	0.1	6.1	19,8	1.6	-2	A	Water, soy protein concentrate 25%, rapeseed oil, natural flavours, table salt.
**58**	Vegane Filet-Streifen	Garden Gourmet	Rewe	232 (971)	12.4	1.0	3.2	2.8	8.5	22,9	0.9	-6	A	Drinking water, 17.6% soy protein, variable amounts of vegetable oils (sunflower, rapeseed), 9.7% wheat protein, apple purée, acidity regulator (buffered vinegar), spices (onion powder, garlic, black pepper), lemon fibres, stabiliser (methylcellulose), yeast extract, maize starch, table salt, spirit vinegar, natural flavours.
**59**	Plant-Based Chicken Chunks	Green Mountain	Edeka	105 (438)	1.4	0.1	2.3	0.1	5.1	18,0	1.0	-4	A	Water, soy protein 27%, natural flavour, rapeseed oil, table salt, iron, vitamin B12.

No.	Products	Brand	Source	Energy kcal/100 g) (kJ/100 g)	Fat (g/100 g)	Sat. fat (g/100 g)	CHO (g/100 g)	Sugar (g/100 g)	Fibre (g/100 g)	Protein (g/100 g)	Salt (g/100 g)	FSAm-NPS	Nutri Score	Ingredients
**60**	Plant-Based Paprika Chunks	Green Mountain	Edeka	128 (537)	5.0	0.4	3.6	1.6	4.5	15,0	2.3	10	C	Water, soy protein 22%, ketchup (tomato paste concentrate, water, glucose syrup, white wine vinegar, raw cane sugar, maize starch, iodised table salt [table salt, potassium iodide]), rapeseed oil, fermented onion extract, garlic, table salt, natural flavour, herbs, colouring paprika extract 0.4%, paprika 0.3%, iodised table salt (table salt, potassium iodide), curry, pepper, iron, vitamin B12.
**61**	Plant-Based Curry Chunks	Green Mountain	Edeka	158 (659)	8.0	3.2	5.3	2.0	4.3	14,0	2.3	13	D	Water, 19% soy protein, 5% coconut milk powder, rapeseed oil, coriander, table salt, garlic, lime juice from concentrate, lemon juice concentrate, pepperoncini, tomato powder, curry 0.8%, iodised table salt (table salt, potassium iodide), turmeric, natural flavour, olive oil, spirit vinegar, iron, vitamin B12.
**62**	Like Chicken	Like Meat	Rewe	103 (431)	1.8	0.2	0.0	0.0	6.5	19,0	0.9	-6	A	Water, soy protein concentrate 25%, sunflower oil, natural flavour, salt, maltodextrin, onion, sugar, spices.
**63**	Like Grilled Chicken	Like Meat	Rewe	115 (481)	2.9	0.4	0.0	0.0	7.1	19,0	1.2	-5	A	Water, soy protein concentrate 24%, sunflower oil, natural flavours, paprika 1.7%, sea salt, spices, herbs, sugar, paprika extract, dextrose.
**64**	The Wonder Chunks Chicken-Style	My Vay	Aldi Nord	107 (447)	1.9	0.3	0.5	0.5	5.8	19,0	1.1	-4	A	Water, 26% soy protein concentrate, sunflower oil, natural flavours, flavouring, salt, spices (paprika powder, white pepper, ground ginger, onion powder, ground nutmeg, ground mace, ground cardamom seed, garlic powder), maltodextrin, sugar.
**65**	The Wonder Chunks Chicken-Style	My Vay	Aldi Süd	132 (554)	3.4	0.4	2.5	0.0	6.1	20,0	1.6	-2	A	Drinking water, 25% soy protein concentrate, rapeseed oil, natural flavours, table salt.
**66**	Veganes Geschnetz eltes Hähnchen Art	My Veggie	Edeka	132 (553)	3.5	0.4	3.7	0.5	5.8	18,5	1.6	-2	A	Water, 25% soy protein concentrate, natural flavour, rapeseed oil, table salt.
**67**	Chicken Kräuter & Zitrone	planted.	Rewe	195 (815)	10.0	1.0	2.2	0.5	4.0	21,0	1.9	10	C	Water, 26% pea protein, rapeseed oil, pea fibres, spices, herbs 0.8% (rosemary, thyme), lemon peel 0.4%, lemon juice concentrate 0.3%, mustard seeds, spirit vinegar, salt, vitamin B12.
**68**	Chicken Natur	planted.	Rewe	145 (607)	3.4	0.6	1.8	0.2	4.3	24,0	1.2	-3	A	Water, 33% pea protein, pea fibres, rapeseed oil, salt, vitamin B12.
**69**	Veganes Mühlen Geschnetz eltes	Rügenwald er Mühle	Rewe	126 (526)	3.3	0.4	1.4	0.5	5.0	20,0	2.0	1	B	Drinking water, 20% soy protein (contains wheat), flavour, rapeseed oil, table salt, spirit vinegar, spices, herbs, protective culture.
**70**	Vegane Chickeriki Streifen	The Vegetarian Butcher	Rewe	142 (595)	3.6	0.4	2.9	0.5	6.2	21,0	1.1	-4	A	89% soy base (drinking water, soy protein), natural flavours, sunflower oil, table salt, acidifier (citric acid), vitamin B12.
**71**	vegane Chunks Chicken Style	Vemondo	Lidl	129 (538)	3.9	0.4	0.5	0.2	5.6	20,0	1.1	-4	A	Drinking water, 25% soy protein, 3% pea protein, rapeseed oil, spirit vinegar, table salt.
**72**	Vegane ~~Hähnchen~~ Spieße Hot & Spicy	Green Legend	Edeka	298 (1,248)	23.0	1.4	6.4	1.2	4.2	15,0	1.2	1	B	Drinking water, rapeseed oil, wheat protein (7%), field bean protein (3%), pea protein (2%), spices, spirit vinegar, cane sugar, modified starch, spice extracts, stabiliser: methylcellulose; table salt, starch, natural flavours, dextrose, thickener: xanthan gum.

No.	Products	Brand	Source	Energy kcal/100 g) (kJ/100 g)	Fat (g/100 g)	Sat. fat (g/100 g)	CHO (g/100 g)	Sugar (g/100 g)	Fibre (g/100 g)	Protein (g/100 g)	Salt (g/100 g)	FSAm-NPS	Nutri Score	Ingredients
**73**	Vegane ~~Hähnchen~~ Spieße Sweet BBQ	Green Legend	Edeka	291 (1,219)	21.0	1.3	9.2	4.3	3.6	15,0	1.3	10	C	Drinking water, rapeseed oil, wheat protein (7%), field bean protein (3%), pea protein (2%), stabiliser: methylcellulose; table salt, spice extract, sugar, wheat flour, apricot juice concentrate, tomato paste, spices, spirit vinegar, smoked salt (salt, smoke), starch, natural flavours, caramel sugar, herbs, thickener: guar gum; smoke flavour, dextrose.
**74**	The Wonder Vegane Grillspieße Curry	My Vay	Aldi Nord	223 (933)	13.0	1.2	6.5	1.4	6.5	17,0	1.9	8	C	Drinking water, 12% rapeseed oil, 11% textured plant protein blend (59% soy protein isolate, 27% wheat protein, wheat starch), 7% textured soy protein concentrate, 3% wheat protein, thickener: methylcellulose; natural flavours, spices (white pepper, turmeric, coriander, fenugreek, cumin, ginger, fennel seed, allspice, cinnamon, mace), wheat flour, fractionated pea flour, sea salt, glucose syrup, table salt, dextrose, turmeric extract.
**75**	The Wonder Vegane Flammens pieße BBQ	My Vay	Aldi Nord	215 (900)	13.0	1.2	4.6	1.3	6.5	17,0	1.9	8	C	Drinking water, 11% rapeseed oil, 11% textured plant protein blend (59% soy protein isolate, 27% wheat protein, wheat starch), 7% textured soy protein concentrate, natural flavours, 3% wheat protein, thickener: methylcellulose; wheat flour, fractionated pea flour, sea salt, spices, sugar, table salt, glucose syrup, herbs, dextrose, tomato powder, spirit vinegar powder, paprika extract, caramel sugar, smoke flavour.
**76**	The Wonder Vegane Flammens pieße Hot	My Vay	Aldi Nord	212 (888)	12.0	1.0	5.8	1.3	7.0	17,0	1.9	7	C	Drinking water, 11% rapeseed oil, 11% textured plant protein blend (59% soy protein isolate, 27% wheat protein, wheat starch), 7% textured soy protein concentrate, 3% wheat protein, thickener: methylcellulose; natural flavours, fractionated pea flour, spices, wheat flour, sea salt, sugar, table salt, herbs, dextrose, tomato powder, paprika extract.
**77**	The Wonder Vegane Flammspie ße Tikka	My Vay	Aldi Nord	227 (948)	13.0	1.2	7.6	1.2	6.1	17,0	1.9	9	C	Drinking water, 12% rapeseed oil, 11% textured plant protein blend (59% soy protein isolate, 27% wheat protein, wheat starch), 7% textured soy protein concentrate, 3% wheat protein, thickener: methylcellulose; natural flavours, wheat flour, fractionated pea flour, spices, sea salt, glucose syrup, table salt, sugar, dextrose, tomato powder, spice extracts (paprika, turmeric extract), lime oil.
**78**	Grillspieß	planted.	Rewe	128 (535)	2.8	0.5	3.8	1.2	4.0	20,0	1.6	0	A	Water, 24% pea protein, rapeseed oil, pea fibres, lemon juice, white wine vinegar, herbs 0.9%, spices 0.7%, salt, corn starch, vitamin B12.
**79**	Grillspieß Kräuter	planted.	Edeka	128 (535)	2.8	0.5	3.8	1.2	4.0	20,0	1.6	0	A	Water, 24% pea protein, rapeseed oil, pea fibres, lemon juice, white wine vinegar, herbs 0.9%, spices 0.7%, salt, corn starch, vitamin B12.
**80**	SoChicken Spieße	SoFine	Edeka	190 (793)	9.4	1.2	7.1	0.8	1.8	18,2	1.4	2	B	Rehydrated soy and wheat protein, water, soybeans, vegetable oils (sunflower, rapeseed) in variable proportions, soy protein, wheat flour, potato starch, thickener (methylcellulose), natural flavour, spirit vinegar, sea salt, firming agent (calcium sulphate), potassium chloride, dextrose, vitamins (B2, B12), mineral (iron pyrophosphate).
**81**	Vegane ~~Hähnch en~~ Filets Classic	Green Legend	Edeka	283 (1,182)	21.0	1.6	5.4	0.3	5.2	16,0	1.2	1	B	Drinking water, rapeseed oil, wheat protein (8%), field bean protein (5%), pea protein (3%), stabiliser: methylcellulose; starch, spice extract, natural flavouring, wheat flour, modified starch (contains wheat), table salt, dextrose.
**82**	Vegane ~~Hähnchen~~ Filets Spicy Herbs	Green Legend	Edeka	341 (1,428)	29.0	2.7	4.4	0.3	5.2	14,0	1.3	10	C	Drinking water, rapeseed oil, wheat protein (7%), field bean protein (4%), pea protein (3%), fully hydrogenated rapeseed oil, herbs (contains celery), starch, stabiliser: methylcellulose; spice extracts, natural flavouring, wheat flour, spices, modified starch (contains wheat), table salt, dextrose.
**83**	Plant-Based Filet	Green Mountain	Edeka	154 (646)	8.3	0.7	5.1	0.5	4.9	12,0	1.5	2	B	Water, textured wheat protein 8.5%, textured soya protein 8.5%, rapeseed oil, thickeners (methylcellulose, xanthan gum), natural flavouring, pea protein 2.5%, fermented onion extract, inulin, spirit vinegar, concentrated lemon juice, spices,

No.	Products	Brand	Source	Energy kcal/100 g) (kJ/100 g)	Fat (g/100 g)	Sat. fat (g/100 g)	CHO (g/100 g)	Sugar (g/100 g)	Fibre (g/100 g)	Protein (g/100 g)	Salt (g/100 g)	FSAm-NPS	Nutri Score	Ingredients
	(Hähnchen -Art)													ketchup (tomato paste concentrate, water, glucose syrup, white wine vinegar, raw cane sugar, maize starch, iodised table salt [salt, potassium iodide]), curry, iodised table salt (salt, potassium iodide), colouring foodstuff: beetroot juice concentrate, iron, vitamin B12.
**84**	Like Chicken Filet	Like Meat	Rewe	174 (728)	9.2	1.1	7.8	0.0	6.1	12,0	1.5	3	C	Water, textured plant protein 15% (wheat protein, soya protein isolate, starch [maize, wheat], pea protein), sunflower oil, gluten-free oat hull fibre, starch, thickener (methylcellulose), natural flavourings, wheat protein, salt, onion powder, dextrose.
**85**	Veganes Steak Typ „Hähnchen steak“ mit Paprikama rinade	My Vay	Aldi Nord	238 (996)	19.0	1.6	5.4	1.8	3.7	10,0	2.9	16	D	90% vegan steak (drinking water, rapeseed oil, 5.4% textured soya protein, 4% wheat gluten, 2.7% textured wheat protein, emulsifiers: methylcellulose, xanthan gum; acidity regulator: potassium lactate; natural flavouring, preservatives: sodium acetate, potassium acetate; bamboo fibre, pea protein, dextrose, maize flour, table salt, firming agent: potassium chloride; thickener: konjac; maltodextrin), 10% marinade (rapeseed oil, spices [contains yellow mustard seed], 0.1% paprika, table salt, sugar, spirit vinegar, palm fat, spice extracts [pepper extract, paprika extract], herbs).
**86**	Chicken Filet Natur	planted.	Edeka	141 (588)	7.3	0.7	1.5	0.3	2.5	16,0	1.2	0	A	Water, pea protein 20%, rapeseed oil, pea fibre, spices (contains mustard), concentrated lime juice, yeast extract, salt, vitamin B12.
**87**	Veganes Filet Hähnchen-Art Paprika	take it veggie	Kaufland	206 (861)	13.0	1.5	3.5	1.5	6.0	16,0	1.5	1	B	Water, soya protein (13%), marinade (10%) (rapeseed oil, onion powder, glucose syrup, salt, tomato powder, maltodextrin, paprika powder, fully hydrogenated rapeseed oil, natural flavouring, smoked chilli powder, spirit vinegar powder, smoke flavouring, beetroot juice concentrate, paprika extract, antioxidant: rosemary extract), rapeseed oil, thickener: methylcellulose, textured wheat protein product (3%) (wheat protein, wheat starch), natural flavouring, yeast extract, salt, pea fibre, potato starch, spices.
**88**	Vegane Steaks nach Art Hähnchen	take it veggie	Kaufland	238 (996)	19.0	1.6	5.4	1.8	3.9	10,0	1.6	5	C	Vegan steaks (90%) (drinking water, rapeseed oil, textured soya protein [5.4%], wheat gluten [4%], textured wheat protein [2.7%], thickeners: methylcellulose, xanthan gum, cellulose, konjac; acidity regulator: potassium lactate; natural flavouring, preservatives: sodium acetate, potassium acetate; pea protein, dextrose, maize flour, table salt, maltodextrin), paprika marinade (10%) (rapeseed oil, spices [contains yellow mustard seed], paprika, table salt, sugar, spirit vinegar, palm fat, spice extracts, herbs).
**89**	Chick-Eria Filets	The Vegetarian Butcher	Rewe	151 (630)	7.2	0.8	3.3	0.5	4.8	16,0	0.9	-3	A	54% soya base (drinking water, soya protein, starch, wheat gluten), drinking water, vegetable oils (sunflower, rapeseed, in variable proportions), soya protein, thickeners (methylcellulose, konjac gum, processed eucheuma seaweed), citrus fibre, natural flavouring, yeast extract, starch, spirit vinegar / alcohol vinegar, table salt, acidity regulators (lactic acid, malic acid), dextrose, vitamin B12.
**90**	Veganes Steak nach Art Hähnchen Pikant mariniert	Vemondo	Lidl	255 (1,065)	18.0	2.0	7.1	1.5	2.9	15,0	1.1	3	C	Drinking water, rapeseed oil, 8.1% textured wheat protein, 5.4% textured pea protein, 3.6% field bean protein, 3.2% wheat protein, emulsifier: methylcellulose; starch, table salt, natural flavourings, spirit vinegar, lemon juice powder, spices, sugar, tomato paste, fully hydrogenated rapeseed oil, vegetables (carrots, white cabbage), herbs.
**91**	Wie Hähnchen brust	Gutfried	Kaufland	178 (746)	12.0	1.6	5.0	2.2	1.6	12,0	2.0	12	D	Drinking water, rapeseed oil (11%), wheat protein (9.5%), pea protein (4.5%), thickeners: carrageenan, methylcellulose, konjac; acidity regulators: calcium carbonate, buffered vinegar; starch (contains wheat), natural flavourings, dextrose, table salt, field bean protein, linseed flour, lemon juice powder, spice extracts.

No.	Products	Brand	Source	**Energy** kcal/100 g) (kJ/100 g)	**Fat** (g/100 g)	**Sat. fat** (g/100 g)	**CHO** (g/100 g)	**Sugar** (g/100 g)	**Fibre** (g/100 g)	**Protein** (g/100 g)	**Salt** (g/100 g)	**FSAm-NPS**	**Nutri Score**	**Ingredients**
**92**	veganer Aufschnitt hauchdünn geschnitten Klassik	Vemondo	Lidl	105 (438)	5.7	0.4	2.8	0.1	2.5	9,4	2.0	7	C	Water, 8% textured wheat protein (6% wheat protein, wheat flour), 5% rapeseed oil, 4% potato protein, thickeners: processed eucheuma seaweed, konjac; natural flavourings, iodised table salt (salt, potassium iodate), dried lentil sourdough, spices, colouring sweet potato concentrate, acidity regulator: buffered vinegar.
**93**	veganer Aufschnitt hauchdünn geschnitten Curry	Vemondo	Lidl	105 (438)	5.7	0.4	2.8	0.1	2.5	9,4	2.0	7	C	Water, 8% textured wheat protein (6% wheat protein, wheat flour), 5% rapeseed oil, 4% potato protein, thickeners: processed eucheuma seaweed, konjac; natural flavourings, iodised table salt (salt, potassium iodate), dried lentil sourdough, spices, herbs, colouring sweet potato concentrate, acidity regulator: buffered vinegar.
**94**	Veganer Hauchschnitt Klassisch - Typ Hähnchen	Rügenwalder Mühle	Rewe	92 (385)	3.6	0.3	3.4	0.5	6.4	8,4	2.0	3	C	Drinking water, 9% wheat gluten, thickeners: carrageenan, konjac; rapeseed oil, bamboo fibre, 2% wheat flour, spice extracts, table salt, hemp protein, natural flavouring, spirit vinegar, spices, colouring foods: concentrates of radish, carrot; concentrated lemon juice.
**95**	Veganer Hauchschnitt Curry - Typ Hähnchen	Rügenwalder Mühle	Rewe	92 (385)	3.6	0.3	3.4	0.5	6.4	8,4	2.0	3	C	Drinking water, 9% wheat gluten, thickeners: carrageenan, konjac; rapeseed oil, bamboo fibre, 2% wheat flour, spice extracts, table salt, hemp protein, 1% curry, natural flavouring, spirit vinegar, spices, colouring foods: concentrates of radish, carrot; concentrated lemon juice.
**Plant-based breaded meat products**														
**96**	Beyond Nuggets Chicken-Style	Beyond Meat	Rewe	287 (1,200)	16.0	1.8	19.0	1.1	4.0	15,0	1.2	2	B	Water, wheat flour, wheat gluten (10%), field bean protein (6%), flavourings, modified maize starch, rapeseed oil, stabilisers: methylcellulose, cellulose; pea starch, herbs and spices, coconut oil, salt, colouring: calcium carbonate; maize starch, rice flour, yeast extract, garlic powder, onion powder, pea protein, dried yeast, sugar, raising agents: diphosphates, sodium carbonates; sunflower oil.
**97**	Beyond Schnitzel	Beyond Meat	Famila	269 (1,125)	14.0	1.9	19.0	1.1	3.6	15,0	1.1	2	B	Water, wheat flour, wheat gluten (10%), field bean protein (6%), flavouring, modified maize starch, rapeseed oil, stabilisers (methylcellulose, cellulose), pea starch, coconut oil, colouring: calcium carbonate, salt, maize starch, rice flour, yeast extract, garlic powder, onion powder, pea protein, raising agents (diphosphates, sodium carbonates), sunflower oil, spices and herbs, dried yeast, sugar.
**98**	Vegane Chicken Nuggets	endori	Edeka	205 (859)	9.6	0.9	14.0	0.5	5.3	14,0	1.5	1	B	Water, rice flour, rapeseed oil, starch, 4% textured pea protein (pea protein, pea flour), 4% pea protein, 3% textured wheat protein (wheat protein, wheat flour), wheat straw fibre, chickpea flour, spirit vinegar (UK: distilled vinegar), thickener: methylcellulose; maize flour, table salt, 0.6% wheat gluten, apple vinegar, spices, wholegrain oat flour, psyllium husk, pea fibre, dextrose, vegetable concentrate (paprika, carrot), natural flavourings.
**99**	Veganes Schnitzel	endori	Edeka	199 (833)	9.1	0.8	16.0	0.6	4.6	11,0	1.5	3	C	Water, 6% textured pea protein (pea protein, pea flour), rapeseed oil, rice flour, 5% pea protein, starch, maize flakes, wheat straw fibre, chickpea flour, spirit vinegar (UK: distilled vinegar), thickener: methylcellulose; maize flour, table salt, spices, wheat gluten, wholegrain oat flour, psyllium husk, dextrose, vegetables (leek, onion, parsnip, white cabbage, garlic, carrot, tomato), pea fibre, carrot juice concentrate powder (maltodextrin, carrot juice concentrate), natural flavourings.

No.	Products	Brand	Source	Energy kcal/100 g) (kJ/100 g)	Fat (g/100 g)	Sat. fat (g/100 g)	CHO (g/100 g)	Sugar (g/100 g)	Fibre (g/100 g)	Protein (g/100 g)	Salt (g/100 g)	FSAm-NPS	Nutri Score	Ingredients
**100**	Vegane Nuggets	Food For Future	Penny	270 (1,130)	16.0	1.1	19.0	0.5	1.3	12,0	1.4	6	C	Water, rapeseed oil, flour (wheat, maize), wheat protein 6%, pea protein 4%, field bean protein 2%, acidity regulators (potassium lactate, potassium acetates), stabiliser (methylcellulose), table salt, starch (contains wheat), natural flavourings, spices, yeast.
**101**	Vegane Schnitzel	Food For Future	Penny	285 (1,193)	16.0	1.2	22.0	1.2	3.4	11,0	1.4	5	C	Water, rapeseed oil, wheat protein 11%, flour (wheat, maize), stabiliser (methylcellulose), modified starch (contains wheat), inulin, acidity regulators (potassium acetates, potassium lactate), durum wheat semolina, table salt, natural flavourings, pea fibre, potato flakes, wheat fibre, spices, yeast.
**102**	Vegane Schnitzel Hähnchen-Art	Garden Gourmet	Kaufland	235 (983)	11.7	0.8	17.6	1.4	5.3	12,3	0.8	-2	A	Drinking water, 16.9% breadcrumb coating (wheat flour, yeast, salt, rapeseed oil, paprika extract, paprika powder, turmeric), 14.4% soya protein, vegetable oils in varying proportions (rapeseed, sunflower), wheat flour, maize starch, natural flavourings, acidity regulator (buffered vinegar), stabilisers (methylcellulose, guar gum), citrus fibre, table salt, onion powder, 0.2% garlic powder.
**103**	Vegane Knusper-Filets Hähnchen-Art	Garden Gourmet	Edeka	246 (1,028)	10.1	1.0	18.2	0.5	3.5	18,6	1.1	0	A	Drinking water, 13.2% breadcrumb coating (wheat flour, wheat starch, rapeseed oil, yeast, table salt), 12.2% soya protein, 9.3% wheat protein, wheat flour, sunflower oil, spirit vinegar, natural flavouring, yeast extract, table salt, sugar, black pepper, thickening agent (locust bean gum).
**104**	„Chicken“ Nuggets mit „Cheese“-Alternative Vegan	Green Cuisine	Edeka	294 (1,231)	17.0	3.6	23.0	0.5	5.3	9,9	0.9	3	C	Rehydrated wheat protein (36%), breadcrumb (wheat flour, paprika, yeast, salt, turmeric), rapeseed oil, water, “cheese” alternative (12%) (water, coconut oil, starch, modified starch, salt, lentil protein, natural flavouring, colouring: beta-carotene), wheat flour, stabiliser: methylcellulose, bamboo fibre, tapioca starch, natural flavouring, garlic powder, salt, white pepper.
**105**	„Chicken“ Nuggets Vegan	Green Cuisine	Edeka	304 (1,274)	16.0	1.2	25.0	0.5	6.6	12,0	1.0	0	A	Rehydrated wheat protein (38%), breadcrumb (wheat flour, whole wheat flour, salt, yeast, paprika powder, turmeric), rapeseed oil, water, wheat flour, maize flour, bamboo fibre, stabiliser: methylcellulose, tapioca starch, natural flavouring, salt, garlic powder, white pepper, onion powder.
**106**	Vegane ~~Chicken~~ Sticks	Green Legend	Edeka	315 (1,320)	19.0	1.4	24.0	0.9	3.0	11,0	1.9	13	D	Drinking water, rapeseed oil, flour (wheat, maize), wheat protein (7%), field bean protein (3%), modified starch (contains wheat), pea protein (2%), durum wheat semolina, stabiliser: methylcellulose; spice extract, starch, table salt, natural flavouring, spices, yeast.
**107**	Vegane ~~Hähnchen~~ Mini-Schnitzel	Green Legend	Edeka	327 (1,367)	22.0	1.7	17.0	0.6	3.0	14,0	1.3	11	D	Drinking water, rapeseed oil, flour (wheat, maize), wheat protein (7%), field bean protein (3%), modified starch (contains wheat), pea protein (2%), durum wheat semolina, stabiliser: methylcellulose; spice extract, starch, table salt, natural flavouring, spices, yeast.
**108**	Plant-Based Schnitzel (Wiener Art)	Green Mountain	Edeka	206 (862)	8.6	0.8	17.0	0.4	4.2	13,0	1.7	3	C	Water, textured wheat protein 18% (wheat protein, wheat flour), breadcrumb (wheat flour, table salt, yeast), vegetable oils (rapeseed, sunflower), fermented onion extract, thickener: methylcellulose, natural flavouring, wheat flour, wheat starch, colouring beetroot juice concentrate, iodised table salt (salt, potassium iodide), iron, vitamin B12.
**109**	Vegane Chicken Nuggets	Iglo Green Cuisine	Kaufland	304 (1,274)	16.0	1.2	25.0	0.5	6.6	12,0	1.0	0	A	Rehydrated wheat protein (38%), breadcrumb (wheat flour, whole wheat flour, salt, yeast, paprika powder, turmeric), rapeseed oil, water, wheat flour, maize flour, bamboo fibre, stabiliser: methylcellulose, tapioca starch, natural flavouring, salt, garlic powder, white pepper, onion powder.
**110**	„Chicken“ Dinos	Iglo Green Cuisine	Kaufland	304 (1,272)	17.0	1.2	24.0	0.5	6.0	11,0	1.0	1	B	Rehydrated wheat protein (39%), rapeseed oil, breadcrumb (wheat flour, salt, paprika powder, yeast, turmeric), water, wheat flour, maize flour, bamboo fibre, stabiliser: methylcellulose, tapioca starch, natural flavouring, salt, garlic powder, white pepper, onion powder.
**111**	Like Schnitzel	Like Meat	Rewe	229 (959)	12.0	1.7	14.0	1.2	5.8	14,0	1.3	1	B	Water, coating 25.4% (wheat flour, sunflower oil, water, wheat bran, salt, sugar, yeast, margarine, barley malt extract), soya protein concentrate 9.5%, textured

No.	Products	Brand	Source	Energy kcal/100 g) (kJ/100 g)	Fat (g/100 g)	Sat. fat (g/100 g)	CHO (g/100 g)	Sugar (g/100 g)	Fibre (g/100 g)	Protein (g/100 g)	Salt (g/100 g)	FSAm-NPS	Nutri Score	Ingredients
														plant protein 4.5% (soya protein isolate 2.2%, wheat protein 1.5%, starch (maize, wheat), pea protein 0.1%), sunflower oil, natural flavourings, gluten-free oat hull fibre, thickener (methylcellulose), salt.
**112**	Like Chicken Burger	Like Meat	Rewe	217 (910)	11.0	1.3	13.0	0.9	4.7	13,0	1.5	3	C	Water, sunflower oil, textured plant protein 11% (wheat protein 4%, soya protein isolate 3%, starch (maize, wheat), pea protein 0.6%), wheat flour, gluten-free oat hull fibre, wheat protein, herbs, spices, fennel, maize flour, raising agent (disodium diphosphate), wheat starch, yeast, yeast extract, modified starch, thickener (methylcellulose), natural flavouring, dextrose, table salt, sugar, colouring (paprika extract).
**113**	Like Nuggets	Like Meat	Edeka	227 (950)	11.0	1.4	17.0	1.0	5.8	12,0	1.3	2	B	Water, wheat flour, textured plant protein 12% (soya protein isolate 6%, wheat protein 3%, starch (maize, wheat), pea protein 0.3%), sunflower oil, gluten-free oat hull fibre, thickener (methylcellulose), soya protein concentrate, table salt, natural flavouring, dextrose, spices, sugar, yeast, yeast extract, rapeseed oil, lemon juice concentrate, colourings (curcumin, paprika extract).
**114**	Like Chicken Wings	Like Meat	Edeka	201 (841)	9.3	1.2	13.0	0.6	7.9	13,0	1.3	-1	A	Water, cornflake coating 15% (maize, sugar, table salt), soya protein concentrate 15%, sunflower oil, gluten-free oat hull fibre, thickener (methylcellulose), natural flavouring, starch, maize flour, table salt, dextrose, emulsifier (xanthan gum).
**115**	Vegane Schnitzel Paprika-Tomate	mein veggie Tag	Aldi Nord	206 (862)	8.9	0.8	16.0	3.7	5.0	13,0	1.8	9	C	Drinking water, wheat flour, rapeseed oil, 8% soya protein concentrate, 5% paprika, 3% textured wheat protein blend (75% wheat protein, wheat flour), 3% tomato purée, sugar, 2% wheat protein, thickeners: methylcellulose, xanthan gum, guar gum; flavourings, gherkins, iodised table salt (salt, potassium iodate), spices (contains mustard flour), modified starch, spirit vinegar, 1% soya protein isolate, dried onion, table salt, dextrose, yeast, potato starch, spice extracts, dried paprika.
**116**	Vegane Schnitzel Klassik	mein veggie Tag	Aldi Nord	221 (923)	10.0	0.7	17.0	2.1	-2.9	14,0	1.8	5	C	Drinking water, wheat flour, rapeseed oil, textured soya protein concentrate 10%, textured wheat protein blend 4% (75% wheat protein, wheat flour), wheat protein 3%, natural flavourings, thickener: methylcellulose; iodised table salt (salt, potassium iodate), soya protein isolate 1%, maize flour, spices, sunflower oil, wheat starch, dextrose, yeast, table salt, potato starch.
**117**	Vegane Schnitzel Brokkoli	mein veggie Tag	Aldi Nord	196 (818)	9.0	1.1	15.0	1.8	3.5	12,0	1.8	10	C	Drinking water, wheat flour, broccoli filling 16% (broccoli 45%, drinking water, modified starch, coconut oil, thickeners: methylcellulose, carrageenan, locust bean gum, xanthan gum; table salt, starch, sugar, dextrose, sea salt, spices, spice extracts (contains celery), colouring: carotene; potato protein), textured soya protein concentrate 8%, rapeseed oil, textured wheat protein blend 3% (75% wheat protein, wheat flour), wheat protein 2%, iodised table salt (salt, potassium iodate), soya protein isolate 1%, dextrose, potato starch, spices, herbs, yeast, flavourings, thickener: methylcellulose.
**118**	Vegane Mini-Schnitzel	My Vay	Aldi Nord	327 (1,367)	22.0	1.7	17.0	0.6	3.2	14,0	1.3	10	C	Drinking water, rapeseed oil, flour (wheat, maize), 7% wheat protein, 3% field bean protein, 2% pea protein, modified starch (contains wheat), durum wheat semolina, natural flavourings, stabiliser: methylcellulose; pea fibre, starch, table salt, spices, yeast.
**119**	Vegane Nuggets	My Veggie	Edeka	270 (1,130)	16.3	1.1	18.9	0.5	1.3	11,5	1.4	6	C	Water, rapeseed oil, wheat flour, 9.0% wheat protein, 7.0% pea protein, 4.0% field bean protein, acidity regulators: potassium lactates, potassium acetates; emulsifier: methylcellulose; maize flour, natural flavouring, table salt, wheat starch, starch, modified starch, spices, yeast.
**120**	Vegane Schnitzel	My Veggie	Edeka	232 (971)	10.0	1.5	20.0	0.8	5.0	13,0	1.4	2	B	22% rehydrated wheat protein, 22% rehydrated soya protein, breadcrumbs (wheat flour, table salt, yeast, spices), water, coating (water, wheat flour), sunflower oil, soya flour, thickener: methylcellulose; natural flavouring, wheat flour, wheat fibre, vinegar, bamboo fibre, sea salt.

No.	Products	Brand	Source	Energy kcal/100 g) (kJ/100 g)	Fat (g/100 g)	Sat. fat (g/100 g)	CHO (g/100 g)	Sugar (g/100 g)	Fibre (g/100 g)	Protein (g/100 g)	Salt (g/100 g)	FSAm-NPS	Nutri Score	Ingredients
**121**	Dino Nuggets	Rewe Beste Wahl	Rewe	285 (1,193)	16.2	1.2	22.0	1.2	3.5	11,3	1.4	5	C	Water, rapeseed oil, WHEAT PROTEIN 11%, flour (WHEAT, maize), stabiliser (methylcellulose), modified starch (contains WHEAT), inulin, durum WHEAT semolina, table salt, natural flavourings, pea fibre, potato flakes, WHEAT fibre, spices, yeast
**122**	Schnitzel	Rewe Beste Wahl	Rewe	285 (1,193)	16.2	1.2	22.0	1.2	3.4	11,3	1.4	5	C	Water, rapeseed oil, WHEAT PROTEIN 11%, flour (WHEAT, maize), stabiliser (methylcellulose), modified starch (contains WHEAT), inulin, acidity regulators (potassium acetates, potassium lactates), durum WHEAT semolina, table salt, natural flavourings, pea fibre, potato flakes, WHEAT fibre, spices, yeast
**123**	Schnitzel	Rewe Beste Wahl	Rewe	299 (1,253)	19.1	1.3	17.3	0.5	3.3	13,3	1.4	4	C	Water, rapeseed oil, flour (WHEAT, maize), WHEAT PROTEIN 6%, pea protein 5%, modified starch (contains WHEAT), field bean protein 3%, durum WHEAT semolina, emulsifier (methylcellulose), starch, table salt, natural flavouring, spices, yeast
**124**	Vegane Mühlen Cordon Bleu	Rügenwalder Mühle	Rewe	208 (872)	11.0	0.9	13.0	1.2	5.0	12,0	1.5	3	C	Drinking water, WHEAT FLOUR, rapeseed oil, 9% SOYA PROTEIN, WHEAT GLUTEN, OAT FIBRE, starch, salt, thickener: methylcellulose, maize flour, natural flavouring, WHEAT STARCH, spirit vinegar, spices, sugar, psyllium husks, potato protein, yeast, colourings: beta-carotene, anthocyanins
**125**	Vegane Mühlen Schnitzel	Rügenwalder Mühle	Rewe	202 (846)	10.0	0.9	11.0	0.6	6.4	14,0	1.9	7	C	Drinking water, WHEAT FLOUR, 11% SOYA PROTEIN, rapeseed oil, WHEAT GLUTEN, OAT FIBRE, salt, thickener: methylcellulose, maize flour, natural flavouring, WHEAT STARCH, spirit vinegar, spices, sugar, psyllium husks, yeast
**126**	Vegane Mühlen Nuggets	Rügenwalder Mühle	Rewe	214 (896)	11.0	1.0	10.0	1.5	3.7	17,0	1.8	3	C	Drinking water, WHEAT FLOUR, 11% SOYA PROTEIN CONCENTRATE, rapeseed oil, WHEAT GLUTEN, 4% SOYA PROTEIN ISOLATE, salt, thickener: methylcellulose, spirit vinegar, maize flour, natural flavouring, WHEAT STARCH, sugar, spices, yeast
**127**	Vegane Mühlen Crispies Brezel	Rügenwalder Mühle	Rewe	245 (1,023)	13.0	1.2	16.0	1.4	6.1	13,0	1.5	8	C	Drinking water, WHEAT flour, rapeseed oil, 9% SOYA protein, WHEAT gluten, OAT fibre, salt, thickener: methylcellulose, maize flour, WHEAT starch, spirit vinegar, natural flavouring, yeast, spices, sugar, psyllium husks, pretzel lye (acidity regulator: sodium hydroxide)
**128**	Vegane Mühlen Crispies Western Style	Rügenwalder Mühle	Rewe	236 (986)	10.0	0.8	20.0	1.3	6.5	12,0	1.9	7	C	Drinking water, WHEAT FLOUR, rapeseed oil, 8% SOYA PROTEIN, WHEAT GLUTEN, maize flour, salt, OAT FIBRE, WHEAT STARCH, modified starch, thickener: methylcellulose, natural flavouring, spirit vinegar, spices, yeast, sugar, psyllium husks, dextrose, colouring: paprika extract
**129**	Vegane Abenteuer Nuggets	Rügenwalder Mühle	Rewe	214 (894)	11.0	0.9	13.0	0.9	6.0	13,0	1.0	-2	A	Drinking water, WHEAT FLOUR, SOYA PROTEIN, rapeseed oil, WHEAT GLUTEN, OAT FIBRE, maize flour, salt, WHEAT STARCH, spirit vinegar, natural flavouring, thickener: methylcellulose, psyllium husks, spices, starch, yeast
**130**	Vegane Schnitzel	take it veggie	Kaufland	268 (1,120)	14.0	1.5	24.0	1.0	3.0	10,0	1.5	11	D	Water, breadcrumb coating [Wheat flour, table salt, yeast, spices, wheat starch, raising agents (sodium carbonates, diphosphates)], rapeseed oil, extruded vegetable protein (12%) [wheat protein (4%), soya protein (4%), potato starch], rice flour, rice extrudate, thickener: methylcellulose; table salt, natural flavouring, sugar, linseed, spices, onions, garlic
**131**	Vegane Nuggets	take it veggie	Kaufland	290 (1,214)	17.0	1.5	23.0	1.0	3.0	10,0	1.8	12	D	Drinking water, breadcrumb coating (wheat flour, table salt, yeast, spices, wheat starch, raising agents: sodium carbonates, diphosphates), rapeseed oil, plant-based protein extrudate (wheat protein (4%), soya protein (4%), potato starch), rice flour, rice extrudate, thickener: methylcellulose; table salt, natural flavouring, sugar, spices, potato starch, linseed, onions, garlic.
**132**	Veganes Cordon Bleu	take it veggie	Kaufland	195 (817)	10.5	0.8	12.1	1.1	6.4	10,1	1.3	0	A	Drinking water, wheat flour, vegetable oils (rapeseed oil, sunflower oil), wheat protein (10%), wheat fibres, modified starch, spices (onion powder, celery, white pepper, paprika), pea fibre, table salt, soya protein concentrate, thickeners (methylcellulose, konjac, carrageenan), natural flavourings (contains wheat), starch (contains wheat), maltodextrin, glucose syrup, yeast, colouring: carotene; colouring foodstuff: red radish concentrate, acid: citric acid.

No.	Products	Brand	Source	Energy kcal/100 g) (kJ/100 g)	Fat (g/100 g)	Sat. fat (g/100 g)	CHO (g/100 g)	Sugar (g/100 g)	Fibre (g/100 g)	Protein (g/100 g)	Salt (g/100 g)	FSAm-NPS	Nutri Score	Ingredients
**133**	Crispy Chickimicki Burger	The Vegetarian Butcher	Rewe	240 (1,005)	16.0	1.1	13.0	1.3	6.0	8,8	1.2	2	B	50% soya base (drinking water, SOYA PROTEIN, WHEAT GLUTEN, starch), WHEAT FLOUR, drinking water, vegetable oils (sunflower, rapeseed, in varying proportions), starch (with WHEAT), thickener (methylcellulose), bamboo fibre, psyllium husk, table salt, natural flavourings, WHEAT GLUTEN, OAT FIBRE, yeast extract, acidity regulator (potassium acetate), dextrose, raising agents (diphosphates, sodium carbonates), spirit vinegar, sugar, yeast, acid (citric acid), spices, spice extracts (with CELERY), lovage, iron diphosphate, vitamin B12.
**134**	Beflügel Nuggets	The Vegetarian Butcher	Rewe	224 (936)	11.0	0.9	19.0	0.5	6.4	9,0	1.5	2	B	58% soya base (drinking water, SOYA PROTEIN), 12% cornflakes (maize, sugar, table salt), vegetable oils (sunflower, rapeseed, in varying proportions), flour (maize, rice), OAT FIBRE, modified starch, natural flavourings, thickener (methylcellulose), acidity regulator (buffered vinegar), starch, table salt, SOYA PROTEIN, sea salt, acid (citric acid), iron diphosphate, vitamin B12.
**135**	Wie'n Schnitzel	The Vegetarian Butcher	Rewe	283 (1,186)	18.0	2.9	18.0	0.7	6.5	9,2	0.9	2	B	Drinking water, 23% soya base (drinking water, SOYA PROTEIN, WHEAT GLUTEN, starch), 20% breadcrumbs (WHEAT FLOUR, WHEAT FIBRE, yeast, table salt), vegetable oils (sunflower, maize, in varying proportions), OAT FIBRE, SOYA PROTEIN, vegetable fats (shea, coconut), WHEAT FLOUR, thickener (methylcellulose), natural flavouring, starch, modified WHEAT STARCH, psyllium husk, spirit vinegar, table salt, acid (lactic acid), pepper, iron diphosphate, vitamin B12.
**136**	Crispy Filets	Tindle	Edeka	238 (997)	12.0	2.9	15.0	0.2	4.9	15,0	1.0	0	A	Water, wheat flour, soya concentrate, sunflower oil, wheat gluten, wheat starch, coconut oil, thickener (methylcellulose), natural flavourings, oat fibre, maize starch, salt, white pepper, yeast.
**137**	Wings	Tindle	Edeka	206 (863)	8.9	2.4	15.0	0.5	4.7	15,0	1.4	2	B	Water, wheat flour, soya concentrate, sunflower oil, wheat starch, wheat gluten, natural flavourings, breadcrumbs (wheat flour, yeast, salt), maize flour, coconut oil, thickener (methylcellulose), oat fibre, wheat grits, spices, salt, modified starch, dextrose, stabiliser (diphosphates), raising agent (sodium bicarbonate).
**138**	Filet Bites	Tindle	Edeka	272 (1,138)	15.0	3.2	18.0	0.6	3.8	14,0	0.6	3	C	Water, wheat flour, soya concentrate, sunflower oil, wheat gluten, wheat starch, maize flour, coconut oil, natural flavourings, thickener (methylcellulose), breadcrumbs (wheat flour, yeast, salt), wheat grits, oat fibre, modified starch, spices, salt, dextrose, stabiliser (diphosphates), raising agent (sodium bicarbonate).
**139**	Pflanzliche s Cordon Bleu	vegan leben	Famila	255 (1,069)	13.0	2.5	22.0	1.7	3.5	11,0	1.0	4	C	Water, breadcrumbs (wheat flour, baker’s yeast, salt, spices), wheat protein 11%, sunflower oil, vegan melting slices with vegetable fat base 7% (water, modified starch [potato, maize], vegetable oil [coconut, rapeseed], sea salt, stabilisers: xanthan gum, locust bean gum; natural flavouring, fermented cane sugar, lemon juice, colouring: carotene), wheat starch, onions, vegan ham 5% (water, rapeseed oil, stabilisers: carrageenan, locust bean gum, xanthan gum; pea protein, spices, maltodextrin, sea salt, beetroot concentrate, spirit vinegar, acidity regulator: calcium citrates), wheat flour, mustard (water, mustard seeds, spirit vinegar, salt, sugar, spices), thickener: methylcellulose; spices, sugar, salt, acidity regulator: citric acid; spirit vinegar, natural flavouring, herbs.
**140**	Vegane Schnitzel	Vehappy	Netto	232 (971)	10.0	1.5	20.0	0.8	5.0	13,0	1.4	2	B	44% rehydrated soya and wheat protein, breadcrumbs (wheat flour, table salt, yeast, paprika powder, turmeric powder), water, coating (water, wheat flour), sunflower oil, soya flour, thickener: methylcellulose; natural flavouring, wheat flour, wheat fibres, vinegar, bamboo fibres, sea salt.
**141**	Vegane Nuggets	Vehappy	Netto	237 (992)	10.0	1.5	20.0	0.8	5.5	14,0	1.3	1	B	42% rehydrated soya and wheat protein, breadcrumbs (wheat flour, table salt, yeast, paprika powder, turmeric powder), water, sunflower oil, coating (water, wheat flour), onions, soya flour, thickener: methylcellulose; natural flavouring, wheat flour, wheat fibres, vinegar, bamboo fibres, spices, sea salt.
**142**	veganes Cordon	Vemondo	Lidl	240 (1,006)	14.0	1.0	20.0	0.5	3.6	7,0	1.4	6	C	Drinking water, coating (wheat flour, wheat starch, table salt, yeast, spices), rapeseed oil, 8% vegan melting slice based on rapeseed oil (drinking water,

No.	Products	Brand	Source	Energy kcal/100 g) (kJ/100 g)	Fat (g/100 g)	Sat. fat (g/100 g)	CHO (g/100 g)	Sugar (g/100 g)	Fibre (g/100 g)	Protein (g/100 g)	Salt (g/100 g)	FSAm-NPS	Nutri Score	Ingredients
	Bleu paniert													0.6% rapeseed oil, modified starch, starch, table salt, flavouring, colouring foodstuff: carrot concentrate), 6% vegan pea protein product in ham style (drinking water, rapeseed oil, thickeners: konjac, processed eucheuma seaweed, locust bean gum, methylcellulose, spirit vinegar, natural flavouring, table salt, 0.1% pea protein, potato starch, sugar, colouring: anthocyanins; spices), 5% textured wheat protein (wheat protein, wheat flour), 5% textured soya protein, rice flour, rice flakes, table salt, natural flavouring, spirit vinegar, potato starch, linseed, thickener: methylcellulose; spices.
**143**	veganes Schnitzel paniert	Vemondo	Lidl	268 (1,120)	14.0	1.5	24.0	1.0	3.0	10,0	1.5	11	D	Drinking water, coating (wheat flour, table salt, yeast, spices, wheat starch, raising agents: sodium carbonates, diphosphates), rapeseed oil, rice flour, rice flakes, 4% wheat protein, 4% soya protein, potato starch, table salt, thickener: methylcellulose; natural flavouring, sugar, linseed, spices.
**144**	vegane Nuggets	Vemondo	Lidl	161 (674)	4.9	0.5	18.1	2.0	7.0	7,6	1.0	-1	A	Drinking water, rapeseed oil, breadcrumbs (wheat flour, table salt, yeast), 5.5% textured soya flour, 5% textured wheat protein (wheat protein, wheat flour, pea fibres), potato starch, maize flour, wheat fibres, oat powder, thickener: methylcellulose; wheat semolina, spices, table salt, wheat gluten, lemon juice concentrate, sugar, sea salt, maltodextrin.
**145**	Veganes Knuspriges Schnitzel Hähnchen-Art	Vivera	Rewe	262 (1,096)	13.0	0.8	20.0	1.9	4.2	13,0	1.6	3	C	62% hydrated plant proteins (water, 13% SOYA PROTEIN, 2% WHEAT PROTEIN), cornflakes (maize, sugar, salt, BARLEY MALT EXTRACT), vegetable oils (sunflower, rapeseed in varying proportions), WHEAT FLOUR, starch (contains WHEAT), spirit vinegar, thickener (methylcellulose), natural flavourings, reduced sodium salt, salt, spices, water, potato fibres, onion powder, garlic powder, iron(II) gluconate, marjoram, vitamin B12.
**146**	Vegane Nuggets	Vivera	Rewe	275 (1,152)	13.0	1.3	20.0	1.9	3.6	17,0	1.8	11	D	62% hydrated plant proteins (water, 17% SOYA PROTEIN, 2% WHEAT PROTEIN), cornflakes (maize, sugar, salt, BARLEY MALT EXTRACT), vegetable oils (sunflower, rapeseed in varying proportions), WHEAT FLOUR, spirit vinegar, starch (contains WHEAT), thickener (methylcellulose), natural flavourings, table salt, reduced sodium salt, onion, potato fibres, dried glucose syrup, dextrose, yeast extract, preservative (sodium diacetate), cumin, spice extract, herbal extract, iron(II) gluconate, vitamin B12.
**147**	Vegane Schnitzel	Vivera	Rewe	247 (1,032)	11.0	1.0	19.0	1.2	3.4	17,0	1.5	3	C	62% hydrated plant proteins (water, 17% SOYA PROTEIN, 2% WHEAT PROTEIN), breadcrumbs (WHEAT FLOUR, yeast, salt), vegetable oils (sunflower, rapeseed in varying proportions), spirit vinegar, WHEAT FLOUR, starch (contains WHEAT), thickener (methylcellulose), natural flavourings, table salt, reduced sodium salt, onion, potato fibres, dried glucose syrup, dextrose, yeast extract, preservative (sodium diacetate), cumin, spice extract, herbal extract, iron(II) gluconate, vitamin B12.
**148**	Pflanzliche Nuggets	vegan leben	Markant	276 (1,154)	13.0	1.5	25.0	2.9	1.5	14,0	1.0	3	C	Water, breadcrumbs (wheat flour, spices, salt, baker's yeast), wheat protein 13%, wheat starch, onions, sunflower oil, wheat flour, mustard (water, mustard seeds, spirit vinegar, salt, sugar, spices), thickener: methylcellulose; sugar, salt, herbs, spices, spirit vinegar, acidifier: citric acid; natural flavouring.
**149**	Pflanzliche Schnitzel	vegan leben	Markant	172 (721)	5.4	0.5	19.0	1.6	5.5	9,1	0.9	0	A	Water, breadcrumbs (wheat flour, baker's yeast, salt), soya protein 11%, onions, sunflower oil, wheat starch, wheat straw fibre, wheat flour, wheat protein 2%, thickener: methylcellulose, carrageenan; spices, salt, yeast extract, acidity regulators: potassium acetate, citric acid; natural flavouring, dried mushrooms, herbs, iron, zinc, vitamin B12.
**150**	Beyond Tenders	Beyond Meat	Rewe	295 (1,235)	17.0	2.1	19.0	1.1	3.5	15,0	1.2	3	C	Water, WHEAT GLUTEN (11%), field bean protein (7%), WHEAT FLOUR, flavourings, rapeseed oil, stabilisers: methylcellulose, cellulose; pea starch, modified maize starch, yeast extract, coconut oil, colouring: calcium carbonate; maize starch, rice flour, garlic powder, onion powder, pea protein, raising agents:

No.	Products	Brand	Source	Energy kcal/100 g) (kJ/100 g)	Fat (g/100 g)	Sat. fat (g/100 g)	CHO (g/100 g)	Sugar (g/100 g)	Fibre (g/100 g)	Protein (g/100 g)	Salt (g/100 g)	FSAm-NPS	Nutri Score	Ingredients
														diphosphates, sodium carbonates; sunflower oil, herbs and spices, dried yeast, sugar.
**Plant-based red meat products**														
**151**	Vegane Chunks Döner Style	Food For Future	Penny	137 (574)	3.9	0.6	4.2	1.9	6.4	18,1	2.5	9	C	Water, soya protein concentrate 25%, rapeseed oil, table salt, natural flavourings, sugar, spices, herbs, dextrose, paprika extract.
**152**	Veganes Gyros	Food For Future	Penny	104 (435)	4.1	0.4	5.9	3.9	5.0	8,4	1.2	2	B	Onions 42%, water, textured soya flour 16%, rapeseed oil, spices, table salt, herbs, tomato powder, spice extracts, thickener (xanthan gum), acidifier (citric acid).
**153**	Plant-Based Kebab	Green Mountain	Edeka	102 (427)	2.8	0.4	3.1	1.3	4.1	14,0	2.3	11	D	Water, soya protein 19%, fermented onion extract, spices, colouring paprika extract, table salt, herbs, natural flavouring, rapeseed oil, raw cane sugar, iodised table salt (table salt, potassium iodide), garlic, iron, vitamin B12.
**154**	Like Gyros	Like Meat	Rewe	111 (463)	2.1	0.3	1.9	0.0	5.9	18,0	2.0	0	A	Water, SOYA PROTEIN CONCENTRATE 25%, marinade 5% (water, sunflower oil, table salt, spices, herbs, spice extracts), natural flavouring, flavouring, table salt.
**155**	Like Döner	Like Meat	Rewe	135 (564)	4.0	0.5	0.9	0.0	7.5	19,0	1.8	-3	A	Water, soya protein concentrate 26%, marinade 6% (rapeseed oil, shea butter, spices, herbs, table salt, spirit vinegar, sugar, dextrose, paprika extract), natural flavouring, flavouring, table salt.
**156**	Like Beef Strips	Like Meat	Rewe	120 (504)	2.4	0.3	5.8	2.8	5.6	16,0	1.8	0	A	Water, soya protein concentrate 23%, sunflower oil, sugar, spices, starch, natural flavouring, table salt, lemon juice powder, caramel syrup powder, yeast extract, herbs, flavouring, colouring (paprika extract).
**157**	Like Sweet & Chili Beef Style	Like Meat	Rewe	105 (440)	0.3	0.0	6.6	5.7	5.6	16,0	2.4	10	C	Water, soya protein concentrate 24%, sugar, tomato puree, table salt, spices, chilli powder, spirit vinegar, pineapple juice concentrate, natural flavouring, flavouring.
**158**	The Wonder Chunks Döner-Style	My Vay	Aldi Nord	113 (472)	3.0	0.4	0.5	0.5	5.6	19,0	1.4	-3	A	Water, 25.6% soya protein concentrate, 4.5% marinade (shea butter, spices (black pepper, cumin, paprika, garlic, chilli, coriander, turmeric, caraway, fenugreek, ginger, fennel), herbs (oregano, rosemary, parsley), salt, spirit vinegar, sugar, dextrose, paprika extract, antioxidant (ascorbic acid), salt, flavouring, maltodextrin, natural flavouring).
**159**	Veganes Geschnetzeltes Nach Gyros Art	My Vay	Aldi Nord	105 (438)	1.5	0.2	1.6	1.2	6.2	18,0	1.9	0	A	Water, 38% onions, 16% soya protein concentrate, sunflower oil, salt, spices, herbs, spice extracts (paprika extract, pepper extract), natural flavouring, onion granulate, paprika powder.
**160**	The Wonder Chunks Döner-Style	My Vay	Aldi Süd	137 (574)	3.9	0.6	4.2	1.9	6.3	18,0	2.5	10	C	Drinking water, 25% soya protein concentrate, rapeseed oil, table salt, natural flavourings, sugar, spices, herbs, dextrose, paprika extract.
**161**	Veganes Geschnetzeltes Gyros Art	My Veggie	Edeka	139 (582)	4.2	0.4	4.5	2.0	6.1	17,7	1.8	-1	A	Water, 25% soya protein concentrate, rapeseed oil, table salt, natural flavouring, sugar, spices, herbs, dextrose, spice extract.
**162**	Kebab Original	planted.	Rewe	212 (889)	12.0	1.0	2.8	0.5	3.8	22,0	2.0	10	C	Water, pea protein 30%, rapeseed oil, pea fibres, spices, mushroom powder, yeast extract, salt, vitamin B12.
**163**	Gyros	Rewe Beste Wahl	Rewe	104 (435)	4.1	0.4	5.9	3.9	5.0	8,4	1.2	2	B	Onions 42%, water, textured soya flour 16%, rapeseed oil, spices, table salt, herbs, tomato powder, spice extracts, thickener (xanthan gum), acidifier (citric acid).

No.	Products	Brand	Source	**Energy** kcal/100 g) (kJ/100 g)	**Fat** (g/100 g)	**Sat. fat** (g/100 g)	**CHO** (g/100 g)	**Sugar** (g/100 g)	**Fibre** (g/100 g)	**Protein** (g/100 g)	**Salt** (g/100 g)	**FSAm-NPS**	**Nutri Score**	**Ingredients**
**164**	Veganes Gyros	take it veggie	Kaufland	110 (460)	4.6	0.4	5.8	2.7	3.5	9,6	1.1	2	B	40% onions, drinking water, 16% soya flour, rapeseed oil, spices, table salt, herbs (parsley, thyme, marjoram, oregano, basil, rosemary), spice extracts, thickener: xanthan gum.
**165**	vegane Chunks Döner Style	Vemondo	Lidl	168 (703)	8.8	0.8	1.6	0.7	5.5	18,0	1.5	-1	A	Drinking water, 18% soya protein, 13% marinade [rapeseed oil, spices, herbs, smoked paprika powder (paprika, oak smoke), table salt], 5% pea protein, spirit vinegar.
**166**	veganes Gyros mit Zwiebeln	Vemondo	Lidl	110 (461)	4.9	0.7	4.2	2.8	4.4	10,2	1.2	0	A	Drinking water, 31% onions, 20% textured soya flour, rapeseed oil, table salt, spices, herbs, tomato powder, onion powder, garlic powder, spice extracts, thickener: xanthan gum; acidity regulator: citric acid.
**167**	Plant-Based Beef Chunks	Green Mountain	Edeka	187 (782)	8.6	1.0	2.5	0.3	1.6	24,0	2.3	13	D	Water, pea protein 28%, rapeseed oil, soya protein 2%, pea fibres, natural flavouring, pea starch, table salt, spices, colouring plant extracts, cassava starch, iron, vitamin B12.
**168**	Pulled BBQ	planted.	Rewe	139 (582)	3.3	0.6	6.6	3.2	3.9	19,0	1.6	0	A	Water, plant proteins 23% (pea, sunflower, oats), tomato puree, apple cider vinegar, apple juice concentrate, pea fibre, rapeseed oil, brown sugar, white wine vinegar, mustard seeds, spirit vinegar, spices, salt, maize starch, yeast extract, vitamin B12.
**169**	Pulled Spicy Kräuter	planted.	Famila	224 (939)	14.0	1.3	3.7	0.8	4.4	19,0	1.8	9	C	Water, plant proteins 23% (pea, sunflower), rapeseed oil, pea fibres, lemon juice, white wine vinegar, herbs 1.7%, spices, salt, maize starch, vitamin B12.
**170**	Vegane ~~Fleisch~~ Steaks Wild Pepper	Green Legend	Edeka	320 (1,340)	26.0	2.7	10.6	1.4	3.3	10,0	1.1	5	C	Drinking water, rapeseed oil, onions, pea protein (8%), chopped sunflower seeds, spices, shea butter, natural flavourings, spice extracts, stabiliser: methylcellulose; table salt, mustard (drinking water, mustard seeds, spirit vinegar, table salt, spices), sugar, herbs, colouring foodstuff: beetroot powder.
**171**	Vegane ~~Fleisch~~ Steaks Smoky BBQ	Green Legend	Edeka	231 (965)	14.0	1.1	15.0	5.6	3.0	10,0	1.1	5	C	Drinking water, rapeseed oil, onions, pea protein (8%), chopped sunflower seeds, sugar, spices, natural flavours, stabiliser: methylcellulose; spice extracts, table salt, mustard (drinking water, mustard seeds, spirit vinegar, table salt, spices), apricot juice concentrate, tomato puree, spirit vinegar, smoked salt (table salt, smoke), caramel sugar, herbs, thickener: guar gum; colouring foodstuff: beetroot powder.
**172**	Plant-Based Steak	Green Mountain	Edeka	163 (681)	8.5	0.8	6.4	2.9	3.5	13,0	1.5	3	C	Water, textured wheat protein 18% (wheat protein, wheat flour), rapeseed oil, thickener methylcellulose, wheat protein 3%, natural flavouring, fermented onion extract, spirit vinegar, colouring beetroot juice concentrate, iodised table salt (table salt, potassium iodide), barley malt extract, wheat flour, iron, vitamin B12.
**173**	Plant-Based Pfeffer Medaillon	Green Mountain	Edeka	181 (756)	9.4	0.8	11.0	2.9	1.1	12,0	1.6	5	C	Water, textured soya protein 9%, textured wheat protein 9%, rapeseed oil, thickener methylcellulose, wheat gluten, natural flavouring, fermented onion extract, spirit vinegar, colouring carrot juice concentrate, colouring beetroot juice concentrate, iodised table salt (table salt, potassium iodide), acidifier lactic acid, barley malt extract, pepper 0.2%, wheat flour, iron sulfate, vitamin B12.
**174**	Vegane Medaillons Kräuter	Greenforce	Rewe	210 (880)	14.0	1.5	6.0	1.0	7.0	12,0	2.0	8	C	90% vegan medallion (drinking water, mushrooms, 15% textured pea protein [pea protein, pea flour], rapeseed oil, thickeners: carrageenan, cellulose, konjac, methylcellulose; flavours, potato starch, defatted sunflower seeds, acidity regulator: sodium acetates; table salt, yeast extract, spice extract, maltodextrin), 10% spice marinade (vegetable oils [rapeseed oil, fully hardened rapeseed oil], spices [0.2% wild garlic, garlic, pepper, onion], seasoning, table salt, sea salt).
**175**	Vegane Medaillons Paprika	Greenforce	Rewe	210 (880)	14.0	1.5	6.0	1.0	7.0	12,0	2.0	8	C	90% vegan medallion (drinking water, mushrooms, 15% textured pea protein [pea protein, pea flour], rapeseed oil, thickeners: carrageenan, cellulose, konjac, methylcellulose; flavours, potato starch, defatted sunflower seeds, acidity regulator: sodium acetates; table salt, yeast extract, spice extract, maltodextrin),

No.	Products	Brand	Source	Energy kcal/100 g) (kJ/100 g)	Fat (g/100 g)	Sat. fat (g/100 g)	CHO (g/100 g)	Sugar (g/100 g)	Fibre (g/100 g)	Protein (g/100 g)	Salt (g/100 g)	FSAm-NPS	Nutri Score	Ingredients
														10% spice marinade (rapeseed oil, spices [chili, garlic, coriander, 0.6% paprika, pepper, onions], table salt, emulsifier: mono- and diglycerides of fatty acids; sugar, herbs, spice extracts).
**176**	Veganes Steak Typ „Schwein" Mit Sweet Chili Marinade	My Vay	Aldi Nord	269 (1,125)	21.0	1.9	7.3	3.3	4.6	11,0	2.4	13	D	90% vegan steak (drinking water, rapeseed oil, 6% textured soya protein, 4.5% wheat protein, 3% textured wheat protein, acidity regulator: potassium lactate; table salt, pea protein, textured soya flour, dextrose, spices (white pepper, onions), glucose syrup, maize flour, natural flavouring, antioxidant: citric acid; colouring foodstuff: sweet potato concentrate; firming agent: potassium chloride; preservatives: sodium acetates, potassium acetate; emulsifiers: methylcellulose, xanthan; thickener: konjac), 10% marinade (rapeseed oil, sugar, spices, table salt, fully hardened rapeseed oil, herbs, spice extracts).
**177**	Veganes Steak Typ „Rindersteak" mit BBQ-Marinade	My Vay	Aldi Nord	240 (1,005)	18.0	1.6	5.8	2.6	4.4	12,0	2.3	12	D	90% vegan steak (drinking water, rapeseed oil, 5.4% textured soya protein, 4.5% wheat gluten, 2.7% textured wheat protein, emulsifier: methylcellulose; acidity regulator: potassium lactate; citrus fibre, colouring foodstuff: beetroot juice concentrate, bamboo fibre, preservatives: sodium acetates, potassium acetate; natural flavouring, flavour, caramelised sugar, table salt, dextrose, firming agent: potassium chloride; maltodextrin), 10% marinade (rapeseed oil, spices (contains yellow mustard seed), table salt, sugar, spirit vinegar, palm fat, spice extracts, herbs).
**178**	Veganes Steak	Vivera	Edeka	199 (834)	10.0	4.7	6.8	1.2	4.9	18,0	1.1	9	C	75% hydrated plant proteins (water, 17% wheat protein, 3% soya protein), plant oils (sunflower, rapeseed in varying proportions), coconut oil, flavours, thickener (methylcellulose), spirit vinegar, wheat flour, plant fibres (sugarcane, citrus), colouring (betalain/red beet), safflower concentrate, starch (contains wheat), reduced sodium table salt, water, spices, table salt, garlic powder, onion powder, mushroom powder, oregano, iron(II) gluconate, barley malt extract, vitamin B12.
**179**	Vegane ~~Fleisch~~ Ribs Sweet & Smoky mit BBQ Glaze	Green Legend	Edeka	266 (1,112)	16.0	1.1	19.0	13.0	5.5	9,0	1.1	9	C	Drinking water, rapeseed oil, pea protein (10%), onions, chopped sunflower seeds, spices (onion, tomato, pepper, garlic, parsnip, lovage), natural flavours, stabilizer: methylcellulose; table salt, spice extract, mustard (drinking water, mustard seeds, spirit vinegar, table salt, spices), sugar, apricot juice concentrate, tomato paste, spirit vinegar, smoked salt (table salt, smoke), caramel sugar, thickener: guar gum; smoke flavours, maltodextrin.<br><br>Ingredients BBQ Glaze: drinking water, sugar, maple syrup, spices, spirit vinegar, table salt, spice extract, herbs, thickener: xanthan.
**180**	Roast Beaf	Peas of Heaven	Rewe	139 (580)	6.0	2.2	2.2	1.6	2.7	7,6	2.4	14	D	Water, vegetable fat (sheaf fat, coconut fat), pea protein, stabilizers (carrageenan, konjac gum, locust bean gum, xanthan, guar gum), vegetable broth, salt, black pepper, preservatives (sodium diacetate, potassium sorbate), natural flavour, onion, dextrose, tomato, flavour, acid regulators (malic acid, lactic acid, potassium lactate), spice.
**181**	Vegane Roastbeef Art	Greenforce	Rewe	211 (881)	5.8	1.1	6.1	2.6	9.6	29,0	1.8	6	C	Water, 32% WHEAT PROTEIN, wheat starch, chickpea flour, vegetables (carrots, beetroot, garlic), extra virgin olive oil, iodized salt (sea salt, potassium iodate), yeast extracts, natural vegetable flavours, antioxidant: ascorbic acid, natural tomato flavour, dextrose, spices, herbs.
**182**	Veganes Virginia-Steak	Wheaty	Edeka	248 (1,038)	11.0	8.8	8.9	3.2	3.9	26,0	1.9	19	E	76% seitan (water, WHEAT protein), coconut fat, yeast extract, onions, oat fibre, spices, paprika, MUSTARD, pepper, rock salt, coriander, wheat flour, psyllium husk powder.
**Plant-based bratwurst**														
**183**	Beyond Sausage	Beyond Meat	Rewe	237 (990)	16.0	7.0	5.0	0.0	3.0	17,0	1.4	14	D	Water, pea protein (16%), coconut oil, sunflower oil, flavours, rice protein, faba bean protein, potato starch, salt, vegetable concentrates (beetroot, carrot, bell

No.	Products	Brand	Source	Energy kcal/100 g) (kJ/100 g)	Fat (g/100 g)	Sat. fat (g/100 g)	CHO (g/100 g)	Sugar (g/100 g)	Fibre (g/100 g)	Protein (g/100 g)	Salt (g/100 g)	FSAm-NPS	Nutri Score	Ingredients
														pepper), apple fibre, stabilizers (methylcellulose, calcium chloride), gelling agent (sodium alginate).
184	Vegane Bratwurst Krakauer-Style	Billie Green	Rewe	309 (1,291)	16.0	1.2	6.7	2.9	3.0	33,0	2.1	14	D	33% WHEAT GLUTEN, drinking water, rapeseed oil, lemon juice from lemon juice concentrate, textured plant protein (WHEAT PROTEIN, pea protein), garlic, spices, citrus fibre, table salt, WHEAT STARCH, vegetables: onion; yeast extract, dextrose, colouring foods: radish concentrate, apple concentrate, black currant concentrate; flavour, beech wood smoke. Edible casing (stabilizers: sodium alginate, konjac gum, guar gum).
185	Vegane Bratwurst	endori	Rewe	200 (838)	12.0	1.0	8.5	3.4	5.0	11,0	1.6	3	C	Water, rapeseed oil, 5% WHEAT GLUTEN, edible casing (gelling agent: sodium alginate; stabilizer: calcium chloride), 4% pea protein, 3% textured pea protein (pea protein, pea flour), starch, thickener: methylcellulose; WHEAT STRAW FIBRE, diced onion, distilled vinegar (AT: wine spirit vinegar), dextrose, table salt, spices, herbs, psyllium husks, natural flavours.
186	Vegane Bratwurst	Food For Future	Penny	199 (833)	17.0	1.6	4.8	2.9	3.8	5,4	2.8	15	D	Water, rapeseed oil 16%, thickeners (carrageenan, konjac, methylcellulose), pea protein 4%, spice extracts, natural flavours, table salt, faba bean protein, dextrose, spices, starch, linseed meal.
187	Sensationa l Bratwurst	Garden Gourmet	Rewe	211 (881)	16.0	1.1	2.3	0.5	6.6	11,6	1.0	-1	A	Drinking water, rapeseed oil, 16.5% SOY PROTEIN, acid regulator (buffered vinegar), natural flavours, stabilizer (methylcellulose), spices (garlic, coriander, nutmeg, ginger, pepper), marjoram, apple puree, lemon fibre, table salt, edible alginate casing (thickener (sodium alginate), stabilizer (calcium chloride), tapioca starch), fruit and plant concentrates (beetroot, bell pepper, carrot).
188	Like Bratwurst	Like Meat	Rewe	142 (596)	7.8	5.3	2.3	0.0	6.6	13,0	1.4	8	C	Water, SOY PROTEIN CONCENTRATE 15%, coconut fat, sunflower oil, potato flakes, edible casing (water, coating agent (calcium alginate)), spices (coriander, marjoram, pepper), spice extracts, psyllium husks, seasoning, thickener (methylcellulose), natural flavour, salt, dextrose.
189	Like Schinken Bratwurst	Like Meat	Edeka	184 (770)	11.0	6.1	7.6	1.3	7.0	11,0	1.5	11	D	Water, pea protein isolate 11.8%, pea fibre, coconut fat, sunflower oil, edible casing (water, gelling agent (calcium alginate), flavour), potato flakes, thickener (methylcellulose), spice extracts, spices, natural flavour, caramel sugar, colouring food (concentrates of radish, apple, black currant), maltodextrin, smoked maltodextrin (maltodextrin, smoke), salt, smoked salt (salt, smoke), colouring agent (iron oxides and iron hydroxides).
190	Vegane Bratwurst	My Veggie	Edeka	212 (887)	17.0	1.4	6.7	2.9	5.0	6,1	2.6	13	D	Water, 16% rapeseed oil, 8.5% pea protein, thickeners: processed Eucheuma algae, methylcellulose, konjac; starch, table salt, spices, sugar, dextrose, natural flavour, citrus fibre, spice extracts, acidifier: citric acid; marjoram.
191	Perfekte Bratwurst	Peas of Heaven	Rewe	225 (942)	20.0	1.5	4.7	0.2	4.3	7,3	1.9	10	C	Water, rapeseed oil, pea protein, starch (pea, potato), salt, thickeners (methylcellulose, processed Eucheuma algae, konjac gum), spices, garlic, citrus fibres, acid regulator (tartaric acid), yeast extract, glucose, flavour, stabilizer (E450).
192	Kesewurst	Peas of Heaven	Rewe	212 (886)	17.0	3.2	6.3	0.0	4.6	6,9	1.9	12	D	Water, rapeseed oil, Violife block (10%) (water, coconut oil, starch, modified starch, sea salt, flavours, olive extract, colour (beta-carotene), vitamin B12), pea protein, stabilizers (processed Eucheuma algae, methylcellulose, konjac gum), starch, salt, citrus fibres, spices, flavours, paprika powder, chili, garlic. Plant casing: stabilizers (sodium alginate, konjac gum, guar gum).
193	Bratwurst Kräuter	planted.	Edeka	196 (820)	14.0	1.9	0.8	0.3	0.5	17,0	1.7	11	D	Water, pea protein 20%, sunflower oil, spices, salt, yeast extract, vitamin B12.
194	Bratwurst	Rewe Beste Wahl	Rewe	199 (833)	17.0	1.6	4.8	2.9	3.8	5,4	2.8	15	D	Water, rapeseed oil 16%, thickeners (carrageenan, konjac, methylcellulose), pea protein 4%, spice extracts, natural flavours, table salt, faba bean protein, dextrose, spices, starch, linseed meal.
195	Vegane Mühlen	Rügenwald er Mühle	Rewe	167 (699)	12.0	1.0	6.4	0.8	3.2	7,1	1.8	7	C	Drinking water, rapeseed oil, 7% WHEAT GLUTEN, onions, starch, thickeners: methylcellulose, carrageenan; natural flavour, spice extracts, table salt, distilled

No.	Products	Brand	Source	Energy kcal/100 g) (kJ/100 g)	Fat (g/100 g)	Sat. fat (g/100 g)	CHO (g/100 g)	Sugar (g/100 g)	Fibre (g/100 g)	Protein (g/100 g)	Salt (g/100 g)	FSAm-NPS	Nutri Score	Ingredients
	Rostbratwürstchen													vinegar, 1% WHEAT FLOUR, spices, potato protein, OAT FIBRES, glucose, herbs.
**196**	Vegane Bundesliga Bratwurst	Rügenwalder Mühle	Rewe	174 (726)	12.0	1.0	6.6	0.6	3.8	8,2	1.9	10	C	Drinking water, rapeseed oil, 7% wheat gluten, onions, starch, thickeners: methylcellulose, carrageenan; natural flavour, spice extracts, table salt, faba bean protein, distilled vinegar, oat fibres, 1% wheat flour, spices, glucose.
**197**	Vegane Mini Bratwurst	take it veggie	Kaufland	183 (767)	13.0	2.0	3.0	1.0	6.0	11,0	2.0	9	C	Drinking water, 13% pea protein, rapeseed oil, mushrooms, thickeners: tara gum, methylcellulose, konjac, processed Eucheuma algae, xanthan; natural flavour, table salt, sugar, spices, herbs, dextrose, onions, edible casing made of alginate (coating agent: sodium alginate; thickener: guar gum; firming agent: calcium chloride).
**198**	Pflanzliche Kräutergriller	vegan leben	Famila	171 (717)	7.7	1.0	7.4	1.3	0.0	18,0	1.8	3	C	Water, isolated soy protein 8.8%, rapeseed oil, textured soy protein 6.6%, wheat protein 5.2%, rice flour, salt, flavours, thickener: carrageenan; emulsifier: methylcellulose; spices, barley malt extract.
**199**	Vegane Bratwurst	Vehappy	Netto	212 (887)	17.0	1.4	6.7	2.9	5.0	6,1	2.6	13	D	Water, 16% rapeseed oil, 8% pea protein, thickeners: processed Eucheuma algae, methylcellulose, konjac; starch, table salt, spices, sugar, dextrose, natural flavour, citrus fibre, spice extracts, acidifier: citric acid; herbs.
**200**	vegane Bratwurst	Vemondo	Lidl	111 (463)	6.0	5.5	2.3	1.2	5.9	9,1	2.6	15	D	Drinking water, 10% pea protein, coconut fat (fully hydrogenated), thickeners: methylcellulose, carrageenan, konjac; table salt, yeast extract, dextrose, spices, psyllium husks, spice extracts, herbs, flavours, acid regulators: calcium lactate, sodium acetates.
**201**	Bruzzler veggie	Wiesenhof	Rewe	191 (801)	12.0	1.0	12.0	3.0	0.0	9,0	2.2	12	D	Drinking water, rapeseed oil (18%), pea protein (5%), thickeners: methylcellulose, carrageenan, konjac; potato starch, table salt, spices, spice extracts, flavour, dextrose, maltodextrin, glucose syrup, sugar.
**202**	Bruzzler veggie Berner Style	Wiesenhof	Rewe	171 (717)	13.0	3.0	7.0	1.0	4.0	5,0	2.0	12	D	65% Vegan Wieners with plant protein: drinking water, rapeseed oil, 4.8% pea protein, potato starch, thickeners: carrageenan, methylcellulose, konjac; table salt, spices, spice extracts, flavours, seasoning (from rapeseed and corn), colorants: iron oxide and iron hydroxide, annatto bixin. 25% Vegan bacon with plant protein: drinking water, 9.5% pea protein, sunflower oil, thickeners: methylcellulose, carrageenan, konjac; fibres (citrus, pea), spices, table salt, spice extracts, flavour, acid regulator: calcium lactate, seasoning (from rapeseed and corn), natural flavour, sugar, carrot juice concentrate, colorants: iron oxides and iron hydroxides, paprika extract. 10% Vegan cheese alternative: drinking water, coconut fat, 70% starch (tapioca, cassava), maltodextrin, thickeners: modified potato starch, xanthan; table salt, natural flavour, acidifier: lactic acid; acid regulator: calcium lactate; colorant: beta-carotene.
**203**	Pflanzliche Currywurst	vegan leben	Famila	175 (733)	9.3	0.8	6.0	0.5	0.0	17,0	1.9	11	D	Water, isolated soy protein 10.9%, rapeseed oil, wheat protein 6.6%, modified starch, textured plant protein 1.8% (isolated soy protein, corn starch, wheat gluten), salt, thickeners: carrageenan, konjac; flavours, vinegar powder, emulsifier: methylcellulose; spice extracts, colorants: iron oxide, iron hydroxide; barley malt extract, smoke flavour.
**204**	Pflanzliche Rostbratwurst	vegan leben	Markant	206 (862)	14.0	1.1	7.3	0.7	1.4	12,0	1.4	5	C	Water, rapeseed oil, textured wheat protein 6%, isolated pea protein 6%, wheat gluten, flavours, thickeners: carrageenan, konjac; modified corn starch, salt, emulsifier: methylcellulose; wheat starch, spices, wheat fibres, vinegar powder, potato protein, spice extracts, glucose, antioxidant: ascorbic acid.
**Plant-based cooked sausages**														
**205**	Vegane Mortadella Klassik	Billie Green	Rewe	144 (603)	10.0	0.8	3.5	1.5	10.0	5,5	1.7	2	B	Drinking water, 10% rapeseed oil, inulin, soy protein isolate, thickeners: carrageenan, processed Eucheuma algae, konjac; distilled vinegar, table salt, flavour, dextrose, flavourings, spices.
**206**	Vegane Mortadella	Billie Green	Rewe	136 (571)	9.0	0.6	5.0	3.2	8.5	5,0	1.7	2	B	Drinking water, 12% pickled cherry peppers (cherry peppers, water, sugar, distilled vinegar), 8.5% rapeseed oil, inulin, soy protein isolate, thickeners:

No.	Products	Brand	Source	Energy kcal/100 g) (kJ/100 g)	Fat (g/100 g)	Sat. fat (g/100 g)	CHO (g/100 g)	Sugar (g/100 g)	Fibre (g/100 g)	Protein (g/100 g)	Salt (g/100 g)	FSAm-NPS	Nutri Score	Ingredients
	Kirschpaprika													carrageenan, processed Eucheuma algae, konjac; distilled vinegar, table salt, flavour, dextrose, flavourings, spices.
**207**	Vegane Mortadella Gartenkräuter	Billie Green	Rewe	144 (603)	10.0	0.8	3.5	1.5	10.0	5,5	1.7	2	B	Drinking water, 10% rapeseed oil, inulin, soy protein isolate, thickeners: carrageenan, processed Eucheuma algae, konjac; distilled vinegar, table salt, flavour, dextrose, spices, herbs, flavourings.
**208**	Vegane Fleischwurst	Food For Future	Penny	128 (537)	10.5	0.3	4.0	2.0	1.8	4,0	2.2	11	D	Water, rapeseed oil, pea protein 2.8%, faba bean protein 1.2%, natural flavours, thickeners (carrageenan, konjac, methylcellulose), rice starch, linseed meal, colouring foods (paprika extract, beetroot juice concentrate), spices, sugar, table salt, dextrose, maltodextrin.
**209**	Veganer Aufschnitt Typ Lyoner Grillgemüse	Food For Future	Penny	164 (685)	14.0	1.0	4.0	1.5	4.0	5,0	1.8	7	C	Water, grilled vegetables 20% (pepper 46.5%, zucchini 24%, green beans), water, tomato paste, distilled vinegar, spices, table salt, antioxidant (ascorbic acid), rapeseed oil, herbs, sugar, thickener (carob bean gum), rapeseed oil, pea protein 4%, faba bean protein 3%, thickeners (processed Eucheuma algae, konjac, methylcellulose), pea flour, spice extracts, starch, natural flavours, colouring foods (beetroot juice concentrate, paprika extract), spices.
**210**	Veganer Aufschnitt Typ Lyoner Paprika	Food For Future	Penny	173 (723)	15.0	1.2	2.0	1.3	4.0	6,0	1.8	10	C	Water, rapeseed oil, pepper 8%, pea protein 4%, faba bean protein 3%, thickeners (processed Eucheuma algae, konjac, methylcellulose), pea flour, spice extracts, starch, natural flavours, colouring foods (beetroot juice concentrate, paprika juice concentrate), spices.
**211**	Veganer Aufschnitt Typ Lyoner	Food For Future	Penny	181 (759)	15.5	1.2	3.0	1.3	4.0	6,0	1.8	10	C	Water, rapeseed oil, pea protein 4%, fava bean protein 3%, thickeners (processed Eucheuma seaweed, konjac, methylcellulose), pea flour, spice extracts, starch, natural flavours, colouring food (red beet juice concentrate, paprika juice concentrate), spices.
**212**	Vegane ~~Geflügel~~ Mortadella Paprika	Green Legend	Edeka	76 (320)	6.5	0.6	1.7	0.1	0.0	3,0	2.0	8	C	Drinking water, rapeseed oil, 8% bell peppers (red, green), 3% pea protein, plant fibres (citrus fibres, pea fibres), thickeners: processed Eucheuma seaweed, konjac; natural flavour, table salt, potato starch, acidity regulator: calcium lactate; colouring food: red radish concentrate.
**213**	Vegane ~~Geflügel~~ Mortadella	Green Legend	Edeka	76 (320)	6.5	0.6	1.7	0.1	0.0	3,0	2.0	8	C	Drinking water, rapeseed oil, 3% pea protein, plant fibres (citrus fibres, pea fibres), thickeners: processed Eucheuma seaweed, konjac; natural flavour, table salt, potato starch, acidity regulator: calcium lactate; colouring food: red radish concentrate.
**214**	Vegane ~~Geflügel~~ Wurst Typ Fleischwurst	Green Legend	Edeka	165 (692)	15.0	1.5	2.7	0.5	5.1	3,0	2.5	13	D	Drinking water, rapeseed oil, 3% pea protein, natural flavour, thickeners: processed Eucheuma seaweed, konjac, methylcellulose; citrus fibre, table salt, dextrose, emulsifier: citric acid esters of mono- and diglycerides of fatty acids; colouring food: red radish concentrate.
**215**	Vegane Mini Wiener	Greenforce	Rewe	109 (455)	6.8	0.8	0.9	0.0	5.3	9,0	2.5	10	C	Drinking water, rapeseed oil, 9% pea protein, thickeners: methylcellulose, processed Eucheuma seaweed, konjac; citrus fibre, table salt, yeast extracts, dextrose, spices, natural flavours, spice extracts, acidity regulators: calcium lactate, sodium acetate; colouring agents: iron oxides and hydroxides; smoke flavour, preservative: potassium sorbate; beechwood smoke.
**216**	Veganer Leberkäse	Greenforce	Rewe	167 (699)	14.0	1.0	1.7	0.3	4.2	7,2	2.5	12	D	Drinking water, rapeseed oil, 4% pea protein, fava bean protein, thickeners: carrageenan, methylcellulose, konjac; flavours, spices, herbs, table salt, pea fibre, seasoning, antioxidant: ascorbic acid; acidity regulators: calcium lactate, sodium acetate; colouring agents: iron oxides and hydroxides.

No.	Products	Brand	Source	Energy kcal/100 g) (kJ/100 g)	Fat (g/100 g)	Sat. fat (g/100 g)	CHO (g/100 g)	Sugar (g/100 g)	Fibre (g/100 g)	Protein (g/100 g)	Salt (g/100 g)	FSAm-NPS	Nutri Score	Ingredients
**217**	Vegane Hot Dogs Classic	Hobelz Veggie World	Famila	242 (1,012)	20.3	1.5	5.3	0.5	2.2	9,0	2.3	15	D	Water, rapeseed oil, isolated soy protein 8.5%, thickeners: processed Eucheuma seaweed; flavours (including yeast extract), dried vegetables (onion, garlic), emulsifier: methylcellulose; salt, sugar, thickener: konjac; wheat fibre, modified corn starch, spices, colorants: iron oxides and hydroxides.
**218**	Pflanzlicher Aufschnitt Grillgemüse	Love Veggie	Edeka	192 (804)	16.0	1.3	4.5	2.6	5.5	4,5	2.9	14	D	Drinking water, 17% rapeseed oil, 13% grilled vegetables (zucchini, bell pepper, tomato, sunflower oil), thickeners: carrageenan, xanthan, konjac; 4% sunflower protein, dextrose, table salt, starch, seasoning, spices, antioxidant: ascorbic acid; natural flavour, sweet potato concentrate, acidifier: citric acid; colouring agent: carotenoids.
**219**	Pflanzlicher Aufschnitt nach Lyoner Art	Love Veggie	Edeka	210 (880)	18.0	1.4	4.1	2.1	6.2	4,9	2.9	14	D	Drinking water, 19% rapeseed oil, thickeners: carrageenan, xanthan, konjac; 5% sunflower protein, dextrose, table salt, starch, seasoning, spices, antioxidant: ascorbic acid; natural flavour, sweet potato concentrate, acidifier: citric acid; colouring agent: carotenoids.
**220**	Pflanzlicher Aufschnitt Dill & Gurke	Love Veggie	Edeka	193 (809)	17.0	1.6	3.5	2.6	5.9	4,5	2.8	13	D	Drinking water, 17% rapeseed oil, thickeners: carrageenan, xanthan, konjac; 9% cucumber, 4% sunflower protein, dextrose, table salt, starch, seasoning, spices, antioxidant: ascorbic acid; natural flavour, sweet potato concentrate, dill, acidifier: citric acid; colouring agent: carotenoids.
**221**	Veganer Aufschnitt Klassik	My Vay	Aldi Süd	146 (611)	12.0	0.9	2.6	1.5	5.6	3,7	2.5	10	C	Drinking water, 12% rapeseed oil, thickeners: processed Eucheuma seaweed, konjac; 2% pea protein, 2% potato protein, natural flavours, iodized table salt (table salt, potassium iodate), dextrose, spices, spice extracts, acidity regulator: sodium acetates, acidifier: citric acid, antioxidant: ascorbic acid, colouring agent: carotene.
**222**	Veganer Aufschnitt Bunter Pfeffer	My Vay	Aldi Süd	146 (612)	12.0	0.9	2.7	1.4	5.6	3,7	2.5	10	C	Drinking water, 12% rapeseed oil, thickeners: processed Eucheuma seaweed, konjac; 2% pea protein, 2% potato protein, natural flavours, iodized table salt (table salt, potassium iodate), dextrose, 1% pepper (green, black), paprika, spices, spice extracts, acidity regulator: sodium acetates, acidifier: citric acid, antioxidant: ascorbic acid, colouring agent: carotene.
**223**	Veganer Aufschnitt Gurke	My Vay	Aldi Süd	138 (577)	12.0	0.8	2.2	1.4	5.5	3,7	2.4	9	C	Drinking water, 12% cucumber pieces (cucumbers, drinking water, spirit vinegar, table salt), 10% rapeseed oil, thickeners: processed Eucheuma seaweed, konjac; 2% pea protein, 2% potato protein, natural flavours, iodized table salt (table salt, potassium iodate), dextrose, spices, spice extracts, acidity regulator: sodium acetates, antioxidant: ascorbic acid, acidifier: citric acid, colouring agent: carotene.
**224**	Veganer Aufschnitt Grillgemüse	My Vay	Aldi Süd	137 (575)	12.0	0.9	2.4	1.5	5.9	3,6	2.2	8	C	Drinking water, 11% rapeseed oil, 7% grilled and pickled vegetable mix (eggplant, artichokes, zucchini, drinking water, table salt, acidifier: citric acid, antioxidant: ascorbic acid), thickeners: processed Eucheuma seaweed, konjac; 2% pickled paprika (paprika, drinking water, spirit vinegar, sugar, table salt), 2% pea protein, 2% potato protein, natural flavours, iodized table salt (table salt, potassium iodate), dextrose, spices, spice extracts, acidity regulator: sodium acetates, antioxidant: ascorbic acid, acidifier: citric acid, colouring agent: carotene.
**225**	Veganer Leverkäse Aufschnitt Fein	My Vay	Aldi Süd	193 (806)	18.0	1.4	2.0	0.0	4.8	4,0	2.0	10	C	Drinking water, 18% rapeseed oil, 4% pea protein, thickeners: methylcellulose, konjac; gelling agent: processed Eucheuma seaweed; natural flavours, iodized table salt (table salt, potassium iodate), spices, dextrose, citrus fibre, spice extracts, colouring agent: iron oxides and iron hydroxides.
**226**	Veganer Leverkäse Aufschnitt Zwiebel	My Vay	Aldi Süd	203 (849)	18.0	1.4	3.7	2.3	5.5	4,5	2.0	9	C	Drinking water, 17% rapeseed oil, 4% toasted onions, 3% pea protein, thickeners: methylcellulose, konjac; gelling agent: processed Eucheuma seaweed; natural flavours, iodized table salt (table salt, potassium iodate), spices, dextrose, citrus fibre, spice extracts, colouring agent: iron oxides and iron hydroxides.

No.	Products	Brand	Source	Energy kcal/100 g) (kJ/100 g)	Fat (g/100 g)	Sat. fat (g/100 g)	CHO (g/100 g)	Sugar (g/100 g)	Fibre (g/100 g)	Protein (g/100 g)	Salt (g/100 g)	FSAm-NPS	Nutri Score	Ingredients
**227**	Veganer Brotzeit Leverkäse Fein	My Vay	Aldi Süd	193 (806)	18.0	1.4	2.0	0.0	4.8	4,0	2.0	10	C	Drinking water, 18% rapeseed oil, 4% pea protein, thickeners: methylcellulose, konjac; gelling agent: processed Eucheuma seaweed; natural flavours, iodized table salt (table salt, potassium iodate), spices, dextrose, citrus fibre, spice extracts, colouring agent: iron oxides and iron hydroxides.
**228**	Veganer Brotzeit Leverkäse Paprika	My Vay	Aldi Süd	200 (836)	18.0	1.4	2.7	1.4	6.0	4,5	2.0	9	C	Drinking water, 17% rapeseed oil, 3% pea protein, 3% paprika, thickeners: methylcellulose, konjac; gelling agent: processed Eucheuma seaweed; natural flavours, iodized table salt (table salt, potassium iodate), spices, dextrose, citrus fibre, spice extracts, colouring agent: iron oxides and iron hydroxides.
**229**	Veganer Aufschnitt Nach Art Lyoner	My Veggie	Edeka	106 (443)	7.7	0.6	4.7	1.2	4.3	2,6	2.1	9	C	Water, plant oils (sunflower, rapeseed), sunflower protein, citrus fibre, potato starch, table salt, spices, spice extracts, thickeners: carrageenan, konjac, xanthan; starch, acidity regulators: sodium lactate, sodium acetates; sugar, dextrose, psyllium husks, colouring foods: radish extract, paprika extract, beetroot powder; acidifier: acetic acid; flavour.
**230**	Vegane Fleischwurst Klassik	My Veggie	Edeka	110 (459)	7.9	0.9	4.4	0.9	5.8	2,7	2.1	8	C	Water, plant oils (sunflower, rapeseed), sunflower protein, citrus fibre, potato starch, table salt, spices, spice extracts, thickeners: carrageenan, konjac, xanthan, methylcellulose; starch, acidity regulators: sodium lactate, sodium acetates; sugar, dextrose, psyllium husks, colouring foods: radish extract, paprika extract, beetroot powder; flavour.
**231**	Veganer Aufschnitt Nach Art Lyoner Mit Pikanter Paprika	My Veggie	Edeka	111 (464)	7.7	0.6	5.6	1.8	4.7	2,7	2.1	9	C	Water, vegetable oils (sunflower, rapeseed), paprika, sunflower protein, citrus fibre, potato starch, table salt, spices, spice extracts, thickeners: carrageenan, konjac, xanthan; starch, acidity regulators: sodium lactate, sodium acetates; sugar, dextrose, psyllium husks, colouring foods: radish extract, paprika extract, beetroot powder; acidifier: acetic acid; flavour.
**232**	Aufschnitt Typ Lyoner	Rewe Beste Wahl	Rewe	181 (759)	15.5	1.2	3.0	1.0	4.0	6,0	1.8	10	C	Water, rapeseed oil, 4% pea protein, 3% fava bean protein, thickeners (processed Eucheuma seaweed, konjac, methylcellulose), pea flour, spice extracts, starch, natural flavours, colouring foods (beetroot juice concentrate, paprika juice concentrate), spices.
**233**	Aufschnitt Typ Paprika-Lyoner	Rewe Beste Wahl	Rewe	173 (723)	15.0	1.2	2.0	1.3	4.0	6,0	1.8	10	C	Water, rapeseed oil, 8% paprika, 4% pea protein, 3% fava bean protein, thickeners (processed Eucheuma seaweed, konjac, methylcellulose), pea flour, spice extracts, starch, natural flavours, colouring foods (beetroot juice concentrate, paprika juice concentrate), spices.
**234**	Wurst Typ Fleischwurst	Rewe Beste Wahl	Rewe	183 (766)	15.0	1.0	1.0	1.0	4.0	5,5	2.4	12	D	Water, rapeseed oil, pea protein 4%, natural flavourings, fava bean protein 3%, thickeners (processed Eucheuma seaweed, konjac, methylcellulose), modified starch, spice extracts, starch, colouring foodstuff (paprika juice concentrate, beetroot juice concentrate), spices
**235**	Veganer Schinken Spicker Mortadella	Rügenwalder Mühle	Rewe	116 (487)	9.0	0.7	3.1	1.5	8.0	2,2	2.2	6	C	Water, rapeseed oil, 4% pea protein, natural flavours, 3% fava bean protein, thickeners (processed Eucheuma seaweed, konjac, methylcellulose), modified starch, spice extracts, starch, colouring foods (paprika juice concentrate, beetroot juice concentrate), spices.
**236**	Veganer Schinken Spicker Grillgemüse	Rügenwalder Mühle	Rewe	120 (502)	9.2	0.7	3.8	2.3	7.4	2,2	2.3	8	C	Drinking water, 9% rapeseed oil, bamboo fibres, thickeners: carrageenan, tara flour, konjac; spice extracts, 2% sunflower protein, table salt, natural flavour, glucose, citrus fibres, spices, potato protein, colorants: anthocyanins, carotenes.
**237**	Veganer Schinkenspicker Schnittlauch	Rügenwalder Mühle	Rewe	141 (590)	12.0	0.7	3.4	1.7	7.1	2,2	2.3	8	C	Drinking water, 12% grilled vegetables [67% paprika, 33% zucchini], rapeseed oil, thickeners: carrageenan, konjac, tara flour; bamboo fibres, spice extracts, 2% sunflower protein, table salt, glucose, natural flavour, citrus fibres, spices, 0.3% herbs, colorants: anthocyanins, carotenes; potato protein.

No.	Products	Brand	Source	Energy kcal/100 g) (kJ/100 g)	Fat (g/100 g)	Sat. fat (g/100 g)	CHO (g/100 g)	Sugar (g/100 g)	Fibre (g/100 g)	Protein (g/100 g)	Salt (g/100 g)	FSAm-NPS	Nutri Score	Ingredients
**238**	Veganer Schinken Spicker Bunte Paprika	Rügenwalder Mühle	Rewe	112 (470)	8.6	0.7	3.5	2.0	6.8	2,2	2.3	8	C	Drinking water, rapeseed oil, 4.4% chives, thickeners: carrageenan, tara flour, konjac; bamboo fibres, spice extracts, 2% sunflower protein, table salt, natural flavour, citrus fibres, spices, glucose, colorants: anthocyanins, carotenes; potato protein.
**239**	Vegane Abenteuer Mortadella	Rügenwalder Mühle	Rewe	109 (458)	8.6	0.7	1.5	0.0	8.6	2,7	1.2	0	A	Drinking water, rapeseed oil, 6% paprika, bamboo fibres, spice extracts, 2% sunflower protein, table salt, glucose, natural flavour, citrus fibres, thickeners: carrageenan, tara flour, konjac; spices, potato protein, colorants: anthocyanins, carotenes.
**240**	Veganer Schinken Spicker Bunter Pfeffer	Rügenwalder Mühle	Rewe	116 (487)	9.0	0.7	3.1	1.5	8.0	2,2	2.3	7	C	Drinking water, rapeseed oil, bamboo fibres, spice extracts, 2% sunflower protein, potato protein, natural flavour, citrus fibres, thickeners: carrageenan, konjac, tara flour; table salt, spices, colorants: anthocyanins, carotenes.
**241**	Veganer Schinken Spicker Typ Fleischwurst	Rügenwalder Mühle	Rewe	221 (923)	16.0	1.5	2.2	0.0	2.7	16,0	1.7	11	D	Drinking water, pea protein, rapeseed oil, natural flavour, bamboo fibres, fava bean protein, table salt, psyllium husks, colouring foods: radish, carrot; spices, spice extracts, distilled vinegar, smoked paprika, smoke flavour.
**242**	Vegane Abenteuer Würstchen	Rügenwalder Mühle	Rewe	167 (699)	12.0	1.0	6.4	0.8	3.2	7,1	1.8	7	C	Drinking water, pea protein, rapeseed oil, natural flavour, fava bean protein, bamboo fibres, psyllium husks, spices, distilled vinegar, colouring foods: concentrates from radish, carrot, paprika; smoke.
**243**	Veganer Aufschnitt mit Broccoli	take it veggie	Kaufland	181 (758)	14.4	1.1	6.5	3.0	4.3	4,7	2.4	12	D	Drinking water, 13% rapeseed oil, 10% broccoli, thickeners: carrageenan, konjac, 3% pea protein, modified starch, iodized table salt (table salt, potassium iodate), natural flavours (contains mustard), 1% fava bean protein, dextrose, starch, spices, acidity regulator: sodium acetates; flaxseed meal, onions, sugar, spice extracts, herbs, paprika juice concentrate, beetroot juice concentrate.
**244**	Veganer Aufschnitt mit Paprika	take it veggie	Kaufland	200 (835)	16.5	1.5	6.8	2.7	4.0	4,5	2.6	14	D	Drinking water, 14% rapeseed oil, thickeners: carrageenan, konjac, 3% pea protein, modified starch, iodized table salt (table salt, potassium iodate), natural flavours (contains mustard), 1% fava bean protein, dextrose, starch, spices, acidity regulator: sodium acetates; flaxseed meal, onions, sugar, spice extracts, herbs, paprika juice concentrate, beetroot juice concentrate, beechwood smoke.
**245**	Pflanzlicher Aufschnitt wie Lyoner	vegan leben	Famila	99 (413)	6.5	0.5	1.3	1.3	5.9	5,9	2.3	9	C	Water, isolated pea protein 7%, rapeseed oil, thickeners: carrageenan, konjac, xanthan; citrus fibre, salt, 1.5% paprika, flavours, white wine vinegar, glucose, dried vegetables (garlic, onion), radish extract, spices, spice extracts, antioxidant: ascorbic acid; psyllium husks.
**246**	veganer Aufschnitt Mortadella-Art	Vemondo	Lidl	81 (339)	4.9	0.4	3.7	0.7	4.0	3,7	1.9	8	C	Drinking water, 4.6% rapeseed oil, 4% soy protein, citrus fibres, modified starch, thickeners: carrageenan, konjac gum, xanthan; table salt, natural flavour, sugar, colouring radish concentrate, spices, dextrose, psyllium husks, spice extracts, antioxidant: ascorbic acid.
**247**	veganer Aufschnitt mit buntem Pfeffer	Vemondo	Lidl	84 (352)	4.9	0.4	4.2	0.6	4.3	3,8	1.9	7	C	Ingredients: Drinking water, 4.6% rapeseed oil, 4% soy protein, citrus fibres, modified starch, thickeners: carrageenan, konjac gum, xanthan; table salt, natural flavour, sugar, 0.9% whole pepper (black, green), colouring radish concentrate, 0.2% crushed pepper (pink, white), spices, dextrose, psyllium husks, spice extracts, antioxidant: ascorbic acid.
**248**	Vegane Weisswurst Art	Greenforce	Rewe	172 (720)	16.0	1.1	1.7	0.0	4.2	4,3	1.8	9	C	Drinking water, rapeseed oil, 4% pea protein, thickeners: methylcellulose, carrageenan, konjac; onions, natural flavours, table salt, pea flour, partly defatted sunflower seeds, parsley, seasoning, spices, acidity regulators: calcium lactate, sodium acetates.

No.	Products	Brand	Source	Energy kcal/100 g) (kJ/100 g)	Fat (g/100 g)	Sat. fat (g/100 g)	CHO (g/100 g)	Sugar (g/100 g)	Fibre (g/100 g)	Protein (g/100 g)	Salt (g/100 g)	FSAm-NPS	Nutri Score	Ingredients
**249**	Veganer Aufschnitt	take it veggie	Kaufland	178 (745)	14.5	1.1	6.0	2.7	3.8	4,5	2.5	14	D	Drinking water, 13% rapeseed oil, 10% paprika, thickeners: carrageenan, konjac, 3% pea protein, modified starch, iodized table salt (table salt, potassium iodate), natural flavours (contains mustard), 1% fava bean protein, dextrose, starch, spices, acidity regulator: sodium acetates; flaxseed meal, onions, sugar, spice extracts, herbs, paprika juice concentrate, beetroot juice concentrate.
**250**	Veganer Aufschnitt mit Schnittlauch	Vemondo	Lidl	82 (345)	5.0	0.4	3.3	0.8	4.7	3,9	1.9	7	C	Drinking water, 4.6% rapeseed oil, 4% soy protein, citrus fibres, modified starch, thickeners: carrageenan, konjac gum, xanthan; table salt, natural flavour, sugar, 0.4% chives, colouring radish concentrate, spices, dextrose, psyllium husks, spice extracts, antioxidant: ascorbic acid.
**251**	Veganer Aufschnitt mit Grillgemüse	Vemondo	Lidl	80 (336)	4.8	0.4	3.9	0.5	3.9	3,6	1.8	8	C	Drinking water, 7.2% grilled vegetables in variable weight proportions [bell peppers (red, green, yellow), zucchini, eggplant, onions], 4.3% rapeseed oil, 3.7% soy protein, citrus fibres, modified starch, thickeners: carrageenan, konjac gum, xanthan; table salt, natural flavour, sugar, colouring radish concentrate, spices, dextrose, psyllium husks, spice extracts, antioxidant: ascorbic acid.
**Plant-based salami**														
**252**	Vegane Salami Baguette Style	Billie Green	Rewe	257 (1,075)	10.0	8.7	8.5	3.4	2.0	32,0	3.0	25	E	Drinking water, 30% WHEAT GLUTEN, 10% textured plant protein (WHEAT PROTEIN, pea protein), coconut fat, garlic, onions; colouring foods: radish concentrate, carrot concentrate, caramelized carrot concentrate, table salt, natural flavour, lemon juice from lemon juice concentrate, WHEAT STARCH, dextrose, yeast extract, spices, citrus fibre, rice flour, beechwood smoke.
**253**	Vegane Salami Klassik	Billie Green	Rewe	265 (1,110)	9.6	0.8	9.5	4.7	3.6	33,0	3.4	19	E	34% WHEAT GLUTEN, drinking water, 12% textured WHEAT PROTEIN (WHEAT PROTEIN, WHEAT FLOUR), rapeseed oil, lemon juice from lemon juice concentrate, colouring foods: radish concentrate, apple concentrate, black currant concentrate, paprika concentrate, carrot concentrate; table salt, garlic, citrus fibre, onion, natural flavour, dextrose, yeast extract, spices, smoked salt (salt, smoke), rice flour, carrot, beechwood smoke.
**254**	Vegane Salami mit Pfeffer	Billie Green	Rewe	265 (1,110)	9.6	0.8	9.5	4.7	3.6	33,0	3.4	19	E	34% WHEAT GLUTEN, drinking water, 12% textured WHEAT PROTEIN (WHEAT PROTEIN, WHEAT FLOUR), rapeseed oil, lemon juice from lemon juice concentrate, colouring foods: radish concentrate, apple concentrate, black currant concentrate, paprika concentrate, carrot concentrate; table salt, garlic, citrus fibre, onion, natural flavour, 1.2% mixed pepper (white, black, green, red), dextrose, yeast extract, spices, smoked salt (salt, smoke), rice flour, carrot, beechwood smoke.
**255**	Snack Salami Style Klassik	Billie Green	Rewe	324 (1,354)	16.0	14.0	10.0	4.3	1.8	34,0	2.7	28	E	31% WHEAT GLUTEN, 13% coconut fat, 9.4% textured plant protein (WHEAT PROTEIN, pea protein), lemon juice from lemon juice concentrate, drinking water, garlic, onion, rapeseed oil, colouring foods: radish concentrate, apple concentrate, black currant concentrate; table salt, yeast extract, WHEAT STARCH, flavour, citrus fibre, vegetable concentrate (caramelized carrot, carrot), spices, beechwood smoke. Edible casing made from alginate (stabilizer: calcium chloride; coating agent: sodium alginate).
**256**	Vegane Salami Chorizo-Style	Billie Green	Rewe	261 (1,091)	10.0	6.3	10.0	4.1	3.0	31,0	3.2	25	E	37% WHEAT GLUTEN, drinking water, coconut fat, garlic, lemon juice from lemon juice concentrate, table salt, 3% textured plant protein (WHEAT PROTEIN, pea protein), rapeseed oil, colouring foods: paprika concentrate, carrot concentrate, radish concentrate, apple concentrate, black currant concentrate, beetroot juice concentrate; ground psyllium husks, onion, carrots, spices, herbs, paprika oil extract, dextrose, smoked paprika (paprika, smoke),

No.	Products	Brand	Source	Energy kcal/100 g) (kJ/100 g)	Fat (g/100 g)	Sat. fat (g/100 g)	CHO (g/100 g)	Sugar (g/100 g)	Fibre (g/100 g)	Protein (g/100 g)	Salt (g/100 g)	FSAm-NPS	Nutri Score	Ingredients
														yeast extract, WHEAT STARCH, citrus fibre, natural flavour, rice flour, tomato powder, spice extract, smoked salt (table salt, smoke), beechwood smoke.
**257**	Snack Salami-Style mit Chili	Billie Green	Rewe	318 (1,332)	16.0	14.0	9.2	4.1	2.8	33,0	2.7	27	E	31% WHEAT GLUTEN, 13% coconut fat, 9.4% textured plant protein (WHEAT PROTEIN, pea protein), lemon juice from lemon juice concentrate, drinking water, garlic, onion, rapeseed oil, colouring foods: radish concentrate, apple concentrate, black currant concentrate; table salt, yeast extract, WHEAT STARCH, flavour, 1% chili, citrus fibre, vegetable concentrate (caramelized carrot, carrot), spices, beechwood smoke. Edible casing made from alginate (stabilizer: calcium chloride; coating agent: sodium alginate).
**258**	Vegane Salami Klassik am Stück	Billie Green	Rewe	265 (1,110)	9.6	0.8	9.5	4.7	3.6	33,0	3.4	19	E	34% WHEAT GLUTEN, drinking water, 12% textured WHEAT PROTEIN (WHEAT PROTEIN, WHEAT FLOUR), rapeseed oil, lemon juice from lemon juice concentrate, colouring foods: radish concentrate, apple concentrate, black currant concentrate, paprika concentrate, carrot concentrate; table salt, garlic, citrus fibre, diced onion, natural flavour, dextrose, yeast extract, spices, smoked salt (salt, smoke), rice flour, carrot, beechwood smoke.
**259**	Veganer Aufschnitt Typ Salami Klassik	Food For Future	Penny	167 (697)	12.0	1.0	4.5	2.0	4.0	8,5	2.0	10	C	Water, rapeseed oil, pea protein 9%, fava bean protein 1.6%, thickeners (processed eucheuma algae, konjac, methylcellulose), spice extracts, starch, flaxseed meal, colouring foods (paprika juice concentrate, beetroot juice concentrate), spices, citrus fibre, natural flavours.
**260**	Vegane ~~Geflügel~~ Salami	Green Legend	Edeka	136 (571)	9.0	0.8	7.0	5.0	0.0	7,0	3.0	16	D	Drinking water, rapeseed oil, 7% plant proteins (pea protein, fava bean protein), thickeners: carrageenan, konjac, methylcellulose; starch, flaxseed meal, colouring food: beetroot juice concentrate; flavours, yeast extract, spices, spice extracts, sugar, colorants: paprika extract, iron oxides and iron hydroxides; smoke flavour, maltodextrin, acidifier: lactic acid; acidity regulator: calcium lactate.
**261**	Vegane Chorizo Art	Greenforce	Rewe	277 (1,160)	14.0	9.0	8.4	4.0	3.3	28,0	3.3	27	E	Water, 33% WHEAT PROTEIN, coconut oil, WHEAT STARCH, sunflower oil, iodized salt (sea salt, potassium iodate), tomato concentrate, beetroot, spices, antioxidant: ascorbic acid, natural flavours, dried red pepper, dextrose, apple vinegar, onion, inactive yeast, garlic, herbs.
**262**	Vegane Wurzerl Chili	My Vay	Aldi Nord	343 (1,435)	25.0	3.0	13.0	7.0	8.5	13,0	3.2	18	D	24% textured soy flour, sunflower oil, drinking water, carrots, 2.4% soy protein, table salt, 1% chilies, starch, spices (chili, paprika), dextrose, spice extracts (chili, paprika), stabilizer: sodium acetate; thickener: processed eucheuma algae, methylcellulose; colouring: beetroot red; natural flavour, edible casing made of alginate (coating agent: sodium alginate; modified starch, firming agent: calcium chloride).
**263**	Vegane Wurzerl Klassik	My Vay	Aldi Nord	343 (1,435)	25.0	3.0	13.0	7.0	8.5	13,0	3.2	18	D	24% textured soy flour, sunflower oil, drinking water, carrots, 2.4% soy protein, table salt, starch, spices (onion, paprika, garlic), dextrose, spice extracts (paprika, pepper), tomato powder, sugar, smoked maltodextrin (maltodextrin, smoke), stabilizer: sodium acetate; antioxidant: ascorbic acid; thickener: processed eucheuma algae, methylcellulose; colouring: beetroot red; natural flavour, smoke flavour, edible casing made of alginate (coating agent: sodium alginate; modified starch, firming agent: calcium chloride).
**264**	Vegane Wurzerl BBQ	My Vay	Aldi Nord	343 (1,435)	25.0	3.0	13.0	7.0	8.5	13,0	3.2	18	D	24% textured soy flour, sunflower oil, drinking water, carrots, 2.4% soy protein, table salt, starch, spices (paprika, black pepper, garlic), sugar, tomato powder, dextrose, spice extracts (chili, lovage), stabilizer: sodium acetate; thickener: processed eucheuma algae, methylcellulose; colouring: beetroot red, ammonium sulfite caramel colour; smoke flavour, edible casing made of alginate (coating agent: sodium alginate; modified starch, firming agent: calcium chloride).
**265**	Veganer Aufschnitt nach Art	My Veggie	Edeka	214 (894)	19.0	1.1	5.5	3.5	1.5	5,0	2.2	14	D	Water, rapeseed oil, pea protein, fava bean protein, pepper mix (black, green, red pepper), thickeners: carrageenan, konjac, methylcellulose, guar gum; modified starch, flaxseed meal, table salt, colouring foods: paprika extract,

No.	Products	Brand	Source	Energy kcal/100 g) (kJ/100 g)	Fat (g/100 g)	Sat. fat (g/100 g)	CHO (g/100 g)	Sugar (g/100 g)	Fibre (g/100 g)	Protein (g/100 g)	Salt (g/100 g)	FSAm-NPS	Nutri Score	Ingredients
	Salami mit Buntem Pfeffer													fenugreek extract, beetroot juice concentrate; natural flavours, spices, spice extracts, sugar, colorants: iron oxides and iron hydroxides.
**266**	Veganer Aufschnitt Nach Salami Art	My Veggie	Edeka	214 (894)	19.0	1.1	5.5	3.5	1.5	5,0	2.2	14	D	Water, rapeseed oil, pea protein, fava bean protein, thickeners: carrageenan, konjac, methylcellulose, guar gum; modified starch, flaxseed meal, table salt, colouring foods: paprika extract, fenugreek extract, beetroot juice concentrate; natural flavours, spices, spice extracts, sugar, colorants: iron oxides and iron hydroxides.
**267**	Perfekte Chorizo	Peas of Heaven	Rewe	229 (959)	18.7	1.4	8.0	1.1	4.4	7,6	1.7	9	C	Water, rapeseed oil, pea protein, starch (pea, potato), thickeners (methylcellulose, processed eucheuma algae, konjac gum), spices, chili, garlic, paprika, citrus fibres, salt, acid regulator (tartaric acid), dextrose, flavours, stabilizer (E450), sugar.
**268**	Aufschnitt Typ Salami	Rewe Beste Wahl	Rewe	176 (737)	12.2	1.5	6.5	2.7	3.2	8,8	2.1	12	D	Water, 11% rapeseed oil, 6% textured pea protein, 3% pea protein, natural flavours, thickeners (processed eucheuma algae, konjac gum, methylcellulose), spice extracts, 1.6% fava bean protein, colouring foods (paprika juice concentrate, beetroot juice concentrate), spices, modified starch, citrus fibre, flaxseed meal.
**269**	Vegane Mühlen Salami Feinschmecker Art	Rügenwalder Mühle	Rewe	231 (967)	7.7	0.6	10.0	2.3	2.4	29,0	2.9	16	D	Drinking water, 34% WHEAT GLUTEN, rapeseed oil, flavour, starch, sunflower protein, colouring foods: concentrates from radish, carrot, caramelized carrot, paprika; table salt, 1% WHEAT FLOUR, bamboo fibres, spices, glucose, smoked cooking salt.
**270**	Vegane Mühlen Salami Klassisch	Rügenwalder Mühle	Rewe	130 (543)	9.1	0.9	3.8	0.5	5.6	5,7	2.4	9	C	Drinking water, rapeseed oil, WHEAT GLUTEN, flavour, bamboo fibres, table salt, potato protein, WHEAT FLOUR, citrus fibres, spices, thickeners: carrageenan, konjac; glucose, colouring foods: concentrate from radish, carrot; colorants: iron oxides, carotenoids.
**271**	Vegestory Snack Piri-Piri	SNACK !T	Kaufland	398 (1,665)	25.0	10.0	18.0	3.7	7.5	22,0	2.5	21	E	Soy protein 34%, rapeseed oil, water, dried onion, plant-based coconut fat, starch, salt, spices including Piri-Piri chillies 0.2%, contains mustard, plant-based fat (shea), gluten-free wheat fibre, spice extracts, flavours, smoke flavour, sugar, beetroot powder 0.4%, antioxidant: sodium ascorbate; iron, vitamin B12.<br>Edible casing – gelling agent: sodium alginate, stabilisers: cellulose, calcium chloride.
**272**	Vegestory Snack 3 Seeds	SNACK !T	Kaufland	436 (1,825)	33.0	8.2	10.0	3.3	7.5	22,0	2.7	21	E	Seeds (sunflower seeds 21%, linseed & chia seeds (Salvia hispanica) 2%), soy protein 19%, water, rapeseed oil, dried onion, plant-based coconut fat, starch, salt, spices (contains mustard), plant-based fat (shea), sugar, gluten-free wheat fibre, spice extracts, flavours, smoke flavour, beetroot powder 0.2%, antioxidant: sodium ascorbate; iron, vitamin B12.<br>Edible casing – gelling agent: sodium alginate, stabilisers: cellulose, calcium chloride.
**273**	Vegestory Snack Original	SNACK !T	Kaufland	396 (1,655)	25.0	10.0	18.0	3.7	7.5	22,0	2.5	21	E	Soy protein 34%, rapeseed oil, water, dried onion, plant-based coconut fat, starch, salt, spices (contains mustard), plant-based fat (shea), gluten-free wheat fibre, spice extracts, flavours, smoke flavour, sugar, beetroot powder 0.2%, antioxidant: sodium ascorbate; iron, vitamin B12. Edible casing – gelling agent: sodium alginate, stabilisers: cellulose, calcium chloride.
**274**	Veganer Snack Salami Art	Veggy Friends	Rewe	274 (1,147)	12.0	0.9	7.3	0.9	2.4	33,0	2.7	16	D	56% seitan (water, wheat protein), water, wheat protein texturate (wheat protein, wheat flour), rapeseed oil, yeast extract, flavours, smoke flavour, soy sauce (water, soybeans, wheat flour, salt), rock salt, spices, onions, mustard flour, thickener: locust bean gum, acidifier: vegan lactic acid, colouring: iron oxides and iron hydroxides, beechwood smoke.

No.	Products	Brand	Source	Energy kcal/100 g) (kJ/100 g)	Fat (g/100 g)	Sat. fat (g/100 g)	CHO (g/100 g)	Sugar (g/100 g)	Fibre (g/100 g)	Protein (g/100 g)	Salt (g/100 g)	FSAm-NPS	Nutri Score	Ingredients
**275**	Vegane Merguez	Wheaty	Edeka	256 (1,073)	12.0	1.4	6.1	1.6	2.8	29,0	1.6	11	D	71% seitan (water, wheat protein), onions, high-oleic sunflower oil, yeast extract, red paprika, spices, garlic, rock salt, thickeners (agar-agar, guar gum and locust bean gum), beechwood smoke.
**Plant-based ham and bacon products**														
**276**	Vegane Schinkenwürfel	Billie Green	Rewe	227 (949)	3.4	0.9	11.0	5.6	3.0	36,0	2.6	15	D	42% wheat gluten, drinking water, garlic, dextrose, lemon juice from lemon juice concentrate, tomato paste, colouring foods: paprika concentrate, radish concentrate, carrot concentrate; table salt, fractionated pea flour, 1.2% textured wheat protein (wheat protein, wheat flour), natural flavour, citrus fibre, smoked salt (salt, smoke).
**277**	Veganer Bacon Klassik	Billie Green	Rewe	229 (960)	6.0	0.8	7.4	5.4	2.3	35,0	2.8	16	D	45% wheat gluten, drinking water, garlic, lemon juice from lemon juice concentrate, rapeseed oil, dextrose, table salt, tomato paste, natural flavour, fractionated pea flour, colouring foods: paprika concentrate, radish concentrate, carrot concentrate; 0.7% textured wheat protein (wheat protein, wheat flour), citrus fibre, smoked salt (salt, smoke), beechwood smoke.
**278**	Veganer Grill-Bacon mit Paprika-Marinade	Billie Green	Rewe	329 (1,378)	17.5	1.7	9.0	5.5	2.0	33,0	2.7	19	E	32% wheat gluten, rapeseed oil, drinking water, lemon juice from lemon juice concentrate, textured plant protein (wheat protein, pea protein), garlic, onion, carrots, dextrose, table salt, yeast extract, glucose syrup, wheat starch, colouring foods: radish concentrate, carrot concentrate, paprika extract; fully hydrogenated rapeseed oil, spices, seasoning (natural flavour, smoked maltodextrin, smoked salt, spices), rice flour, pea protein, sugar, flavour, dried garlic, dried vegetable paprika, herbs, rice starch, caramelised sugar syrup.
**279**	Vegane Kochschinken Art	Greenforce	Rewe	228 (953)	10.0	1.1	9.1	4.2	1.8	24,0	1.8	12	D	Water, 19% textured plant protein (wheat, wheat starch, pea), 9% wheat protein, sunflower oil, 2% pea protein, gelling agent: agar-agar, natural flavours, vegetable concentrate (radish, carrot), seasoning [brown sugar, yeast extract (yeast extract, salt), maize flour, natural flavours, smoked paprika, caramelised sugar, onion powder, salt, rapeseed oil], potato protein, salt, apple cider vinegar, acid regulator: lactic acid, dextrose.
**280**	Vegane Bresaola Art	Greenforce	Rewe	202 (844)	2.0	0.5	14.0	3.7	4.4	30,0	2.2	11	D	Water, 37% wheat protein, 7% wheat starch, vegetables (beetroot, carrots, onion, garlic), yeast extracts, chickpea flour, natural flavour, iodised salt (sea salt, potassium iodate), extra virgin olive oil, dextrose, antioxidant: ascorbic acid, herbs, natural sandalwood aroma, spices.
**281**	Wie Lachsschinken	Gutfried	Rewe	64 (269)	1.3	0.5	8.5	3.5	3.0	3,1	2.6	13	D	Drinking water, modified starches 6%, fava bean protein 3%, trehalose, thickeners: carrageenan, locust bean gum, konjac, table salt, rapeseed oil 1%, rice flour, natural flavours, spirit vinegar, maize starch, lemon juice powder, citrus fibre, colouring foodstuffs: paprika, red beet juice concentrate, sweet potato powder; firming agent: potassium chloride, acidity regulator: sodium carbonates, beechwood smoke.
**282**	Wie Lachsschinken Pfeffer	Gutfried	Rewe	64 (269)	1.3	0.5	8.5	3.5	3.0	3,1	2.6	13	D	Drinking water, modified starches 6%, fava bean protein 3%, trehalose, thickeners: carrageenan, locust bean gum, konjac, table salt, rapeseed oil 1%, rice flour, black pepper 1%, natural flavours, spirit vinegar, maize starch, lemon juice powder, citrus fibre, colouring foodstuffs: paprika, red beet juice concentrate, sweet potato powder; firming agent: potassium chloride, acidity regulator: sodium carbonates, beechwood smoke.
**283**	Veganer Bacon	My Veggie	Edeka	160 (671)	11.0	1.6	4.0	1.2	6.7	8,4	2.6	11	D	Water, 13% sunflower oil, pea texturate, 4.1% pea protein, flavour, thickeners: carrageenan, methylcellulose, konjac; 1.7% fava bean protein, modified starch, starch, dextrose, linseed flour, colouring foodstuffs: paprika juice concentrate, red beet juice concentrate; acidity regulator: buffered vinegar; spice extracts, spices, lemon juice powder, beechwood smoke.

No.	Products	Brand	Source	Energy kcal/100 g) (kJ/100 g)	Fat (g/100 g)	Sat. fat (g/100 g)	CHO (g/100 g)	Sugar (g/100 g)	Fibre (g/100 g)	Protein (g/100 g)	Salt (g/100 g)	FSAm-NPS	Nutri Score	Ingredients
**284**	Vegane Baconwürfel	My Veggie	Edeka	160 (671)	11.0	1.6	4.0	1.2	6.7	8,4	2.6	11	D	Water, 12% sunflower oil, pea texturate, flavour, 3.9% pea protein, thickeners: carrageenan, methylcellulose, konjac; modified starch, 1.6% fava bean protein, colouring foodstuffs: paprika juice concentrate, red beet juice concentrate; starch, dextrose, linseed flour, acidity regulator: sodium acetates; spice extracts, spices, lemon juice powder, beechwood smoke.
**285**	Veganer Speck	take it veggie	Kaufland	95 (398)	0.6	0.1	2.1	0.6	5.0	18,0	3.3	15	D	86% rehydrated soy protein [water, soy (30%)], natural flavour, vinegar, salt, preservatives (potassium diacetate, potassium acetate), colour (iron oxide), smoke flavour.
**286**	Veganer Speck	Vivera	Rewe	90 (376)	0.5	0.1	1.8	0.9	4.8	17,0	2.8	12	D	92% hydrated plant protein (water, 24% soy protein), spirit vinegar, flavour, natural flavours, salt, beetroot powder, smoke flavours, water, ferrous gluconate, colour (beta-carotene), vitamin B12.
**Plant-based spreadable sausages**														
**287**	Wie Teewurst	Gutfried	Netto	383 (1,602)	36.0	15.0	8.5	0.8	1.0	7,0	2.0	23	E	Drinking water, rapeseed oil (20%), coconut fat (15%), pea protein isolate (7%), starches (maize, tapioca), rice flour, table salt, linseed flour, dextrose, spices, smoked maltodextrin (maltodextrin, smoke), smoked table salt (salt, smoke), acidifier: citric acid; colour: iron oxides and hydroxides; flavours.
**298**	Wie Leberwurst	Gutfried	Netto	247 (1,035)	20.5	1.6	7.5	1.5	4.8	6,5	2.0	11	D	Drinking water, rapeseed oil 19.5%, pea protein 5%, modified starches, bamboo fibres, fava bean protein, table salt, tomatoes, sugar, spices, herbs, roasted onions (onions, spices, sunflower oil), dextrose, thickener: konjac; colouring foodstuff: beetroot powder; maltodextrin, natural flavours, colour: paprika extract, iron oxides and hydroxides.
**289**	Vegane Pastete Mediterrane Art	Kips	Edeka	256 (1,073)	22.0	8.0	8.4	1.7	1.3	6,2	1.5	17	D	Rehydrated soy protein 30%, plant oils (sunflower, coconut), rehydrated pea protein 16%, vegetables 14% (onion, beetroot, carrot purée, red pepper), green olives 4%, sun-dried tomatoes 2.9%, modified maize starch, maltodextrin, drinking water, salt, concentrated tomato paste, spices, hydrolysed maize protein, dextrose, antioxidants: E300, E301, mushroom extract, yeast extract.
**290**	Vegane Streichwurst Cremig	Kips	Edeka	224 (937)	19.0	10.0	6.2	1.2	1.5	6,8	1.5	18	D	Rehydrated soy protein 31%, rehydrated pea protein 24%, plant oils (coconut, sunflower), vegetables 11% (onion, carrot purée, beetroot), drinking water, maltodextrin, potato starch, modified maize starch, pea fibre, spices, hydrolysed maize protein, salt, concentrated tomato paste, dextrose, antioxidants: E301, E300, mushroom extract, yeast extract, natural flavour, radish concentrate.
**291**	Vegane Streichwurst Nach Leberwurst Art	Kühlmann	Edeka	320 (1,339)	29.0	2.8	12.0	3.2	0.3	3,6	2.7	18	D	Drinking water, rapeseed oil 28%, plant protein (fava bean protein), starch, modified starch, sugar, table salt (partly smoked), natural flavour, tomato powder, spices, vinegar, emulsifiers: mono- and diglycerides of fatty acids, lecithins; colouring foodstuff (concentrate of radish and carrot), spice extracts, colour: iron oxides and hydroxides.
**292**	Vegane Pommersche Schnittlauch	Rügenwalder Mühle	Rewe	219 (916)	17.0	1.2	7.3	0.7	5.1	7,2	2.1	11	D	Drinking water, rapeseed oil, 7% pea protein, starch, 3% chives, inulin, sunflower protein, fava bean protein, citrus fibres, table salt, pea fibres, sugar, spices, natural flavour, colouring foodstuffs: concentrates of radish, carrot, paprika; smoke flavour.
**293**	Vegane Teewurst fein	Rügenwalder Mühle	Rewe	242 (1,014)	22.0	3.8	6.6	0.6	5.0	2,8	2.1	14	D	Drinking water, rapeseed oil, starch, shea butter, flavour (contains smoke), 2.4% pea protein, citrus fibres, bamboo fibres, table salt, spices, pea fibres, psyllium husks, colouring foodstuffs: concentrates of radish, carrot.
**294**	Vegane Pommersche	Rügenwalder Mühle	Rewe	217 (908)	16.0	1.2	8.9	1.2	6.1	6,8	2.0	9	C	Drinking water, 20% onions, rapeseed oil, 11% apples, 7% pea protein, starch, inulin, sunflower protein, fava bean protein, citrus fibres, table salt, pea fibres, sugar, spices, natural flavour, colouring foodstuffs: concentrates of radish, carrot, paprika; smoke flavour.

No.	Products	Brand	Source	**Energy** kcal/100 g) (kJ/100 g)	**Fat** (g/100 g)	**Sat. fat** (g/100 g)	**CHO** (g/100 g)	**Sugar** (g/100 g)	**Fibre** (g/100 g)	**Protein** (g/100 g)	**Salt** (g/100 g)	**FSAm-NPS**	**Nutri Score**	**Ingredients**
**295**	Veganes Mühlen Mett fein	Rügenwalder Mühle	Rewe	66 (278)	2.5	0.2	4.7	1.1	4.7	4,0	1.9	6	C	Drinking water, 25% onions, starch, 3% pea protein, citrus fibres, natural flavour, rapeseed oil, table salt, psyllium husks, spices, pea fibres, thickeners: carrageenan, konjac, guar gum; colouring foodstuffs: concentrates of radish, paprika, carrot; spice extracts, potato protein.
**296**	Veganes Mühlen Mett Chili	Rügenwalder Mühle	Rewe	64 (267)	2.6	0.2	4.2	1.4	5.1	4,0	2.0	6	C	Drinking water, 25% onions, starch, 3% chilli, 3% pea protein, citrus fibres, rapeseed oil, table salt, natural flavour, psyllium husks, spices, pea fibres, thickeners: carrageenan, konjac, guar gum; colouring foodstuffs: concentrates of radish, paprika, carrot; spice extracts, potato protein.
**297**	Vegane Pommersche fein	Rügenwalder Mühle	Rewe	217 (910)	17.0	1.2	7.0	0.7	5.1	7,1	2.1	11	D	Drinking water, rapeseed oil, 7% pea protein, starch, inulin, sunflower protein, fava bean protein, citrus fibres, table salt, pea fibres, sugar, spices, natural flavour, colouring foodstuffs: concentrates of radish, carrot, paprika; smoke flavour.
**298**	Veganer Abenteuer Streich	Rügenwalder Mühle	Rewe	210 (878)	17.0	1.2	5.8	0.0	6.1	6,0	1.2	3	C	Drinking water, rapeseed oil, 5% pea protein, starch, inulin, sunflower protein, natural flavour, citrus fibres, pea fibres, spices, colouring foodstuffs: concentrates of radish, carrot, paprika; table salt, smoke flavour.

Table S6: Nutritional composition of all animal-based meat and sausage products included in the analyses, grouped by subcategory. Sat. fat = saturated fat.

No.	Product	Source	Energy (kcal/100 g) (kJ/100 g)	Fat (g/100 g)	Carbohydrates (g/100 g)	CHO (g/100 g)	Sat. Fat (g/ 00 g)	Salt (g/100 g)	Fibre (g/100 g)	Protein (g/100 g)	FSAm-NPS-Score	Nutri-Score
**Minced meat products**												
**1**	Minced beef, cooked	BLS	266 (1,113)	17.4	0.1	0.1	7.8	0.6	0.0	27.5	12	D
**2**	Minced beef, raw	BLS	207 (866)	14.0	0.1	0.0	6.2	0.6	0.0	20.5	8	C
**3**	Beef meatball, fried (prepared without added fat)	BLS	238 (996)	12.8	8.0	1.3	5.4	1.2	0.8	22.5	13	D
**4**	Minced pork, cooked	BLS	342 (1,431)	27.3	0.0	0.0	11.9	0.8	0.0	24.8	17	D
**5**	Minced pork, raw	BLS	276 (1,155)	22.5	0.0	0.0	9.8	0.6	0.0	19.0	14	D
**6**	Minced pork, frozen	BLS	276 (1,155)	22.5	0.0	0.0	9.8	0.6	0.0	19.0	14	D
**7**	Pork meatball, fried (prepared without added fat)	BLS	289 (1,209)	19.4	7.8	1.2	8.2	1.2	0.8	20.8	17	D
**8**	Minced pork/beef, raw	BLS	233 (975)	17.6	0.0	0.0	7.4	0.6	0.0	18.9	11	D
**9**	Minced pork/beef, frozen	BLS	233 (975)	17.6	0.0	0.0	7.4	0.6	0.0	18.9	11	D
**10**	Minced beef/pork, raw	BLS	224 (937)	16.4	0.1	0.0	7.0	0.6	0.0	19.4	12	D
**11**	Minced beef/pork, frozen	BLS	224 (937)	16.4	0.1	0.0	7.0	0.6	0.0	19.4	13	D
**12**	Beef/pork meatball, fried (prepared without added fat)	BLS	264 (1,105)	16.1	7.9	1.3	6.8	1.2	0.8	21.6	15	D
**13**	Chopped steak, raw	BLS	203 (849)	15.3	1.0	0.8	6.4	1.6	0.2	15.4	16	D
**14**	Pork meatball (standard recipe)	BLS	240 (1,004)	17.6	5.7	0.8	7.4	1.5	0.5	14.9	16	D
**15**	Poultry meatball	BLS	126 (527)	3.6	5.6	0.6	1.4	0.9	0.5	17.4	-1	A
**16**	Rindfleisch Burger Patties	Rewe	250 (1,046)	20.0	0.5	0.5	8.0	1.5	0.0	17.0	17	D
**17**	Wagyu Beef Burger Patties	Rewe	295 (1,234)	25.0	0.5	0.5	10.0	1.5	0.0	17.0	19	E
**18**	Rindfleisch Hamburger	Rewe	224 (937)	17.0	0.8	0.6	7.4	1.5	0.0	17.0	16	D
**19**	Hamburger	Rewe	254 (1,063)	20.0	0.5	0.0	9.4	1.2	0.0	18.0	17	D
**20**	Cevapcici aus Rindfleisch	Rewe	233 (975)	18.0	0.7	0.7	7.0	1.5	0.0	17.0	15	D
**21**	Mühlen Frikadellen	Rewe	271 (1,134)	21.0	4.4	1.6	8.2	1.9	0.0	16.0	20	E
**22**	Rindfleisch-Frikadellen	Rewe	218 (912)	13.0	8.0	1.3	5.5	1.6	0.5	17.0	14	D
**23**	Frikadellen Bällchen	Rewe	310 (1,297)	23.0	12.5	0.4	8.4	1.6	0.0	13.0	18	D
**24**	Original Köttbullar	Rewe	274 (1,146)	22.0	7.5	1.1	9.8	1.4	0.0	13.0	18	D
**25**	Angus Burger	Rewe	246 (1,029)	20.0	0.0	0.0	8.5	0.9	0.0	17.0	15	D

No.	Product	Source	Energy (kcal/100 g) (kJ/100 g)	Fat (g/100 g)	Carbohydrates (g/100 g)	CHO (g/100 g)	Sat. Fat (g/ 00 g)	Salt (g/100 g)	Fibre (g/100 g)	Protein (g/100 g)	FSAm-NPS-Score	Nutri-Score
**26**	Fleischklösschen	Rewe	225 (941)	17.0	7.0	0.0	6.8	1.7	0.0	11.0	16	D
**27**	Block Burger	Rewe	246 (1,029)	20.0	0.0	0.0	8.5	0.9	0.0	17.0	15	D
**28**	Cevapcici	Rewe	257 (1,075)	20.0	1.0	0.5	9.5	1.3	0.0	18.0	18	D
**29**	Mühlen Frikadellen Geflügel	Rewe	196 (820)	13.0	5.7	2.1	4.0	2.0	0.0	14.0	14	D
**30**	Geflügel Frikadellen Mini	Rewe	232 (971)	15.0	9.8	1.2	4.9	1.6	0.7	14.0	13	D
**Chicken products**												
**31**	Roasting chicken meat (medium-fat), raw	BLS	166 (695)	9.6	0.0	0.0	2.8	0.6	0.0	19.9	-1	A
**32**	Roasting chicken meat without skin, raw	BLS	132 (552)	5.7	0.0	0.0	1.7	0.6	0.0	20.0	-3	A
**33**	Roasting chicken meat without skin, fried (prepared without added fat)	BLS	141 (590)	5.5	0.0	0.0	1.7	0.6	0.0	22.9	-3	A
**34**	Roasting chicken meat with skin, raw	BLS	166 (695)	9.6	0.0	0.0	2.7	0.6	0.0	19.9	-1	A
**35**	Roasting chicken meat with skin, frozen	BLS	166 (695)	9.6	0.0	0.0	2.8	0.6	0.0	19.9	-1	A
**36**	Roasting chicken leg, raw	BLS	173 (724)	11.2	0.0	0.0	3.7	0.6	0.0	18.2	0	A
**37**	Roasting chicken leg, frozen	BLS	173 (724)	11.2	0.0	0.0	4.0	0.6	0.0	18.2	0	A
**38**	Roasting chicken breast with skin, raw	BLS	166 (695)	9.6	0.0	0.0	2.8	0.6	0.0	19.9	-1	A
**39**	Roasting chicken breast fillet, raw	BLS	102 (427)	0.7	0.0	0.0	0.2	0.6	0.0	23.6	-4	A
**40**	Marinated roasting chicken leg, grilled	BLS	180 (753)	12.0	0.7	0.5	4.0	0.6	0.2	17.5	1	B
**41**	Marinated roasting chicken breast fillet, grilled	BLS	112 (469)	1.9	0.7	0.5	0.4	0.6	0.2	22.7	-3	A
**42**	Döner meat (poultry), grilled	BLS	267 (1,117)	17.5	0.0	0.0	5.8	0.7	0.0	27.7	11	D
**43**	Hähnchen Filetstreifen	Rewe	115 (481)	1.9	1.4	0.6	0.6	1.3	0.0	23.0	0	A
**44**	Original MrChicken Döner	Rewe	198 (828)	10.1	4.5	2.4	3.2	1.3	0.0	22.2	11	D
**45**	Kebab aus Hähnchenfleisch	Rewe	204 (854)	15.0	5.7	0.7	5.6	1.6	0.0	14.0	14	D
**46**	Hähnchenbrust Filetstücke Paprika	Rewe	128 (536)	3.5	0.5	0.5	0.8	2.8	0.0	23.0	14	D
**47**	Hähnchen Filetstreifen Klassik	Rewe	136 (569)	4.0	3.0	1.8	0.4	3.0	0.0	22.0	15	D
**48**	Hähnchensteaks Paprika	Rewe	111 (464)	3.0	2.0	1.3	0.8	1.8	0.0	19.0	2	B
**49**	Hähnchen Innenfiletspieße Paprika	Rewe	109 (456)	2.9	0.9	0.7	0.6	1.0	0.3	19.6	-1	A
**50**	Hähnchenbraten Roast Chicken	Rewe	98 (410)	1.5	1.0	1.0	0.5	2.2	0.0	20.0	11	D
**51**	Hauchzartes Hähnchenbrustfilet gepökelt, gebacken	Rewe	105 (439)	2.0	0.6	0.5	0.5	2.3	0.5	21.0	12	D
**52**	Hähnchenbrust Filetstücke Klassik	Rewe	122 (510)	2.7	2.1	0.9	0.4	1.3	0.0	22.0	0	A

No.	Product	Source	Energy (kcal/100 g) (kJ/100 g)	Fat (g/100 g)	Carbohydrates (g/100 g)	CHO (g/100 g)	Sat. Fat (g/ 00 g)	Salt (g/100 g)	Fibre (g/100 g)	Protein (g/100 g)	FSAm-NPS-Score	Nutri-Score
**53**	Hähnchen Filetsteaks	Rewe	100 (418)	1.5	0.7	0.5	0.5	1.0	0.0	21.0	-2	A
**54**	Hähnchen-Geschnetzeltes	Rewe	164 (686)	10.0	0.2	0.1	2.3	1.4	0.1	18.0	3	C
**55**	Hähnchen-Oberschenkel	Rewe	226 (946)	18.0	0.0	0.0	5.4	0.7	0.0	16.0	4	C
**56**	2 Hähnchensteaks Barbecue	Rewe	111 (464)	3.0	2.0	1.3	0.8	1.8	0.0	19.0	2	B
**57**	Bedford Hähncheninnenfilets	Rewe	143 (598)	4.3	1.0	0.5	0.8	2.5	0.0	25.0	13	D
**Breaded meat products**												
**58**	Chicken Nuggets	Rewe	186 (778)	7.5	13.0	1.1	0.6	0.7	1.0	16.0	-1	A
**59**	Chicken Dippers	Rewe	281 (1,176)	13.0	26.0	0.8	1.4	1.0	1.8	14.0	3	C
**60**	Schweine Schnitzel	Rewe	190 (795)	8.0	11.0	1.0	2.0	1.7	1.0	18.0	11	D
**61**	Feine Landschnitzel Wiener Art	Rewe	232 (971)	10.0	19.0	1.0	2.3	1.4	0.9	17.0	8	C
**62**	Wiener Kalbsschnitzel	Rewe	142 (594)	1.5	15.0	1.2	0.8	1.2	0.0	16.5	4	C
**63**	Schweine Mini Schnitzel	Rewe	228 (954)	10.0	17.0	1.0	1.4	1.0	1.0	17.0	5	C
**64**	Mini Cordon Bleu	Rewe	234 (979)	12.0	14.0	1.1	3.9	1.4	1.0	17.0	11	D
**65**	Schweine Schnitzel Wiener Art	Rewe	216 (904)	10.0	15.0	0.8	1.5	1.2	1.0	16.0	6	C
**66**	Chicken Nuggets Classic	Rewe	263 (1,100)	11.0	26.0	0.5	1.3	1.0	1.8	14.0	3	C
**67**	Chicken Nuggets im Backteig	Rewe	207 (866)	11.0	11.9	1.3	1.6	1.0	0.4	14.9	1	B
**68**	Chicken Burger	Rewe	194 (812)	7.5	16.7	0.9	0.7	1.0	1.2	14.4	0	A
**69**	Dino & Friends	Rewe	225 (941)	10.5	18.4	1.1	1.1	1.0	0.0	14.1	1	B
**70**	Hähnchen Schnitzel	Rewe	193 (808)	7.5	12.0	0.5	1.0	1.6	0.0	19.0	2	B
**71**	Höhenrainer Puten-Schnitzel	Rewe	186 (778)	7.0	14.0	1.0	1.5	2.7	0.0	16.5	16	D
**72**	Hähnchen Mini Schnitzel	Rewe	222 (929)	10.1	17.1	0.8	0.9	1.0	1.6	14.9	0	A
**73**	Hähnchen Schnitten Cordon Bleu Art	Rewe	215 (900)	11.3	12.6	0.9	2.4	1.1	0.0	14.9	3	C
**74**	Chicken-Nuggets	Rewe	298 (1,247)	20.0	20.0	0.4	7.9	2.1	0.0	9.7	20	E
**75**	Chicken Cheese Nuggets	Rewe	270 (1,130)	14.1	17.7	2.1	3.2	1.0	0.0	17.3	3	C
**76**	Safari Mix Chicken Nuggets	Rewe	259 (1,084)	13.0	22.0	0.5	1.6	1.1	1.0	14.0	4	C
**77**	Roasting chicken breast fillet in batter, deep-fried (prepared without added fat)	BLS	182 (761)	7.7	12.1	0.2	2.4	0.2	0.5	15.8	-2	A
**78**	Roasting chicken breast fillet, breaded, fried	BLS	230 (962)	9.8	15.1	0.9	3.9	0.7	1.0	19.7	1	B
**79**	Roasting chicken wings, marinated, in batter, deep-fried (prepared without added fat)	BLS	252 (1,054)	16.8	12.3	0.9	5.3	0.4	0.6	12.9	4	C

No.	Product	Source	Energy (kcal/100 g) (kJ/100 g)	Fat (g/100 g)	Carbohydrates (g/100 g)	CHO (g/100 g)	Sat. Fat (g/ 00 g)	Salt (g/100 g)	Fibre (g/100 g)	Protein (g/100 g)	FSAm-NPS-Score	Nutri-Score
80	Breaded chicken schnitzel	BLS	263 (1,100)	11.7	22.8	0.9	4.1	0.7	1.3	15.9	4	C
81	Breaded chicken breast, fried (prepared without added fat)	BLS	209 (874)	7.5	18.7	0.8	2.1	0.8	1.1	16.0	2	B
82	Pork cordon bleu (1)	BLS	221 (925)	10.2	9.2	1.1	5.8	0.8	0.7	22.3	11	D
83	Breaded pork topside schnitzel (standard recipe)	BLS	260 (1,088)	12.9	14.2	0.9	4.9	0.9	0.9	21.4	11	D
84	Breaded pork neck schnitzel (standard recipe)	BLS	300 (1,255)	19.2	12.5	0.8	7.7	0.9	0.8	19.2	14	D
85	Breaded pork loin schnitzel (standard recipe)	BLS	256 (1,071)	12.5	12.9	0.8	4.8	0.9	0.8	22.5	11	D
86	Breaded pork topside rump schnitzel (standard recipe)	BLS	246 (1,029)	10.0	16.0	0.9	3.9	0.7	1.0	22.3	7	C
**Red meat products**												
87	Schweine-Rückensteaks in Kräutermarinade	Rewe	147 (615)	6.0	2.0	1.0	3.0	1.3	0.5	21.0	7	C
88	Gyros-Pfanne vom Schwein	Rewe	158 (661)	9.5	1.9	1.3	2.5	0.8	0.0	16.8	4	C
89	Schweine-Nackensteaks in Paprikamarinade	Rewe	176 (736)	10.0	0.9	0.5	5.0	1.0	0.3	19.0	8	C
90	Pulled Pork	Rewe	133 (556)	6.0	2.5	0.5	2.4	1.5	0.0	17.0	8	C
91	Schweinegeschnetzeltes nach Gyros-Art	Rewe	148 (619)	8.0	1.0	1.0	3.7	1.3	0.0	18.0	8	C
92	Beef fillet, raw	BLS	121 (506)	4.0	0.0	0.0	1.8	0.6	0.0	21.2	2	B
93	Beef steak, raw	BLS	146 (611)	6.4	0.0	0.0	2.8	0.6	0.0	22.0	3	C
94	Beef steak (lean), marinated, grilled	BLS	140 (586)	5.5	0.7	0.5	2.0	0.6	0.2	21.6	4	C
95	Beef fillet steak, raw	BLS	121 (506)	4.0	0.0	0.0	1.8	0.6	0.0	21.2	2	B
96	Beef loin (roast beef) (lean), cooked	BLS	167 (699)	4.9	0.0	0.0	2.1	0.6	0.0	30.4	4	C
97	Gyros	BLS	196 (820)	11.1	1.4	1.0	2.5	1.4	0.3	22.7	8	C
98	Pork fillet, marinated, fried (prepared without added fat)	BLS	117 (490)	3.2	0.7	0.5	0.9	0.6	0.2	21.2	1	B
99	Pork steak (medium-fat), marinated, grilled	BLS	177 (741)	10.7	0.7	0.5	3.8	0.6	0.2	19.8	5	C
100	Pork schnitzel (medium-fat), marinated, grilled	BLS	179 (749)	6.7	0.0	0.0	2.4	0.8	0.0	29.6	5	C
101	Pork neck (collar) (lean), marinated, fried (prepared without added fat)	BLS	203 (849)	14.5	0.7	0.5	5.5	0.6	0.2	17.7	7	C
**Bratwurst**												
102	Polish bratwurst	BLS	277 (1,159)	21.4	0.3	0.2	8.4	3.1	0.1	21.3	26	E
103	Farmhouse bratwurst	BLS	306 (1,280)	25.4	0.2	0.2	10.4	2.2	0.1	20.0	23	E
104	Smoked bratwurst	BLS	287 (1,201)	22.9	0.2	0.2	9.4	2.2	0.0	20.7	22	E
105	Bratwurst (not cured)	BLS	273 (1,142)	25.1	0.3	0.3	10.3	1.7	0.1	12.2	21	E

No.	Product	Source	Energy (kcal/100 g) (kJ/100 g)	Fat (g/100 g)	Carbohydrates (g/100 g)	CHO (g/100 g)	Sat. Fat (g/ 00 g)	Salt (g/100 g)	Fibre (g/100 g)	Protein (g/100 g)	FSAm-NPS-Score	Nutri-Score
**106**	Bratwurst / Rhenish bratwurst	BLS	273 (1,142)	25.1	0.3	0.3	10.3	1.7	0.1	12.2	21	E
**107**	Curry bratwurst	BLS	273 (1,142)	25.1	0.3	0.3	10.3	1.7	0.1	12.2	21	E
**108**	Coarse bratwurst / coarse pork bratwurst	BLS	289 (1,209)	25.6	0.3	0.3	10.5	1.3	0.1	15.2	19	E
**109**	Rostbratwurst	BLS	329 (1,377)	29.5	0.3	0.3	12.1	1.5	0.1	16.5	21	E
**110**	Rostbratwurst, heated	BLS	339 (1,418)	30.2	0.3	0.3	12.4	1.6	0.1	17.4	22	E
**111**	Rostbratwurst, grilled	BLS	339 (1,418)	30.2	0.3	0.3	12.4	1.6	0.1	17.4	22	E
**112**	Rostbratwurst, fried (prepared without added fat)	BLS	339 (1,418)	30.2	0.3	0.3	12.4	1.6	0.1	17.4	22	E
**113**	Bratwurst (1)	BLS	289 (1,209)	26.6	0.3	0.3	11.0	1.8	0.1	12.8	21	E
**114**	Poultry bratwurst	BLS	116 (485)	2.8	0.3	0.3	1.1	2.8	0.1	21.9	15	D
**115**	Bruzzzler original	Rewe	231 (967)	19.0	1.0	0.5	6.2	2.1	0.0	14.0	18	D
**116**	Thüringer Rostbratwurst	Rewe	268 (1,121)	23.0	1.4	1.4	9.6	2.1	0.0	14.0	22	E
**117**	Schinken-Würstchen	Rewe	281 (1,176)	25.0	1.0	1.0	10.0	2.2	0.0	13.0	22	E
**118**	Rost-Bratwurst	Rewe	285 (1,192)	25.0	1.0	0.5	11.2	2.0	0.0	14.0	22	E
**119**	Schinken-Würstchen	Rewe	299 (1,251)	27.0	1.0	0.1	10.5	2.1	0.0	13.0	23	E
**120**	Krakauer mit Emmentaler	Rewe	312 (1,305)	27.3	0.5	0.5	12.7	1.9	0.0	15.7	22	E
**121**	Schinken-Griller	Rewe	303 (1,268)	27.0	1.0	0.5	11.2	2.2	0.0	14.0	23	E
**122**	Bratmaxe	Rewe	256 (1,071)	22.0	0.5	0.5	8.8	1.9	0.0	14.0	20	E
**123**	8x Grillbratwurst	Rewe	285 (1,192)	25.0	1.0	0.5	10.5	2.1	0.0	14.0	23	E
**124**	Delikatess Rostbratwurst	Rewe	276 (1,155)	24.0	1.0	1.0	9.6	1.8	0.1	14.0	20	E
**125**	Berner Würstchen	Rewe	296 (1,238)	26.0	1.0	0.5	12.0	2.3	1.0	14.0	24	E
**126**	4x Schinken-Krakauer	Rewe	290 (1,213)	26.0	1.0	0.6	9.7	2.1	0.0	13.0	22	E
**127**	Curry-Bockwurst in Eigenhaut	Rewe	279 (1,167)	25.0	0.5	0.5	10.5	2.0	0.0	13.0	22	E
Cooked sausage products												
**128**	Sausages / bockwurst / Vienna sausages	BLS	271 (1,134)	24.5	0.3	0.3	9.2	1.8	0.1	13.1	20	E
**129**	Munich white sausage	BLS	297 (1,243)	27.2	2.1	2.0	8.2	1.6	0.1	11.7	18	D
**130**	Lyoner sausage	BLS	306 (1,280)	29.2	0.3	0.2	10.9	1.7	0.1	11.4	21	E
**131**	Ham sausage	BLS	260 (1,088)	23.0	0.3	0.2	8.2	1.6	0.1	13.5	19	E
**132**	Northern German mortadella	BLS	309 (1,293)	29.2	0.4	0.3	11.9	2.1	0.2	12.0	23	E
**133**	Paris-style meat sausage	BLS	306 (1,280)	29.2	0.3	0.2	11.0	1.7	0.1	11.4	21	E

No.	Product	Source	Energy (kcal/100 g) (kJ/100 g)	Fat (g/100 g)	Carbohydrates (g/100 g)	CHO (g/100 g)	Sat. Fat (g/ 00 g)	Salt (g/100 g)	Fibre (g/100 g)	Protein (g/100 g)	FSAm-NPS-Score	Nutri-Score
**134**	Calf meat loaf	BLS	321 (1,343)	30.1	0.2	0.2	11.6	1.9	0.0	13.4	23	E
**135**	Meat loaf	BLS	150 (628)	8.3	0.3	0.3	3.4	2.1	0.0	18.5	14	D
**136**	Meat sausage	BLS	300 (1,255)	28.3	0.2	0.0	11.0	2.0	0.0	12.1	22	E
**137**	Cooked sausage, finely chopped (not cured)	BLS	293 (1,226)	27.7	0.4	0.0	11.0	1.8	0.0	11.6	22	E
**138**	Cooked sausage, coarsely chopped (cured)	BLS	313 (1,310)	28.7	0.2	0.1	11.5	1.6	0.0	14.5	20	E
**139**	Coarse ham sausage / coarse Lyoner	BLS	293 (1,226)	25.2	0.2	0.2	10.4	1.8	0.1	17.0	22	E
**140**	Poultry mortadella	BLS	236 (987)	19.1	4.0	3.8	5.5	2.4	0.1	12.6	19	E
**141**	Geflügel Wiener	Rewe	227 (950)	19.0	1.0	0.5	6.0	2.3	0.0	13.0	18	D
**142**	Geflügel Jagdwurst	Rewe	213 (891)	17.0	1.0	0.5	5.3	2.3	0.0	14.0	18	D
**143**	Hähnchen-Mortadella "frische Paprika"	Rewe	232 (971)	19.5	1.0	0.5	5.9	2.2	0.0	13.0	17	D
**144**	Geflügel Mortadella	Rewe	236 (987)	20.0	1.0	0.5	6.0	2.4	0.0	13.0	18	D
**145**	Geflügel Lyoner	Rewe	230 (962)	19.0	1.0	1.0	5.5	2.8	0.0	13.3	20	E
**146**	Geflügel-Fleischwurst	Rewe	234 (979)	20.0	0.5	0.5	6.0	2.2	0.0	13.0	17	D
**147**	Hähnchen-Lyoner	Rewe	238 (996)	20.0	0.5	0.5	6.0	2.2	0.0	14.0	17	D
**148**	Geflügel Mortadella Paprika	Rewe	240 (1,004)	20.0	2.0	0.5	6.3	2.5	0.0	13.0	20	E
**149**	Geflügel-Bierschinken	Rewe	149 (623)	9.0	1.0	0.5	2.7	2.2	0.0	16.0	13	D
**150**	Bärchen Geflügel-Wurst	Rewe	200 (837)	15.0	1.0	0.5	6.0	2.3	0.0	15.0	18	D
**151**	Geflügel-Schinkenwurst	Rewe	238 (996)	20.0	0.5	0.5	6.0	2.0	0.0	14.0	16	D
**152**	Geflügel-Fleischwurst	Rewe	189 (791)	15.0	0.5	0.5	4.8	2.3	0.1	13.0	17	D
**153**	Geflügel-Mortadella	Rewe	238 (996)	20.0	0.5	0.5	6.0	2.2	0.0	14.0	17	D
**154**	Geflügel Mortadella	Rewe	204 (854)	17.0	0.6	0.5	6.5	2.2	0.5	12.0	18	D
**155**	Geflügel-Lyoner mit Paprika	Rewe	206 (862)	17.0	1.0	0.6	5.5	2.0	0.5	12.0	16	D
**156**	Geflügel-Leberkäse	Rewe	202 (845)	15.0	2.7	2.0	4.5	2.2	0.0	14.0	16	D
**157**	Münchner Weißwürste	Rewe	273 (1,142)	25.0	1.0	0.5	10.0	1.6	0.0	11.0	19	E
**158**	10 Stück Wiener Würstchen	Rewe	281 (1,176)	25.0	1.0	1.0	10.0	2.0	0.0	13.0	21	E
**159**	Hot Dog Würstchen	Rewe	226 (946)	19.0	0.7	0.7	7.6	2.0	0.0	13.0	18	D
**160**	Feine Extra	Rewe	206 (862)	17.0	1.3	0.5	6.5	2.3	0.0	12.0	19	E
**161**	Bärchen Wurst	Rewe	192 (803)	15.0	1.0	0.5	6.0	2.3	0.0	13.0	18	D
**162**	Knacker Geschnitten	Rewe	229 (958)	19.0	2.5	0.5	7.4	2.5	0.0	12.0	21	E

No.	Product	Source	Energy (kcal/100 g) (kJ/100 g)	Fat (g/100 g)	Carbohydrates (g/100 g)	CHO (g/100 g)	Sat. Fat (g/ 00 g)	Salt (g/100 g)	Fibre (g/100 g)	Protein (g/100 g)	FSAm-NPS-Score	Nutri-Score
**163**	Mortadella Con "Pistacchio Verde Di Bronte DOP"	Rewe	296 (1,238)	26.0	0.5	0.5	8.6	2.3	0.0	15.0	22	E
**164**	Mortadella mit Champignons	Rewe	258 (1,079)	23.0	0.6	0.5	10.0	2.1	0.5	12.0	22	E
**165**	Saftige Fleischwurst	Rewe	297 (1,243)	27.5	1.0	1.0	11.1	2.1	0.5	11.2	23	E
**166**	Lyoner	Rewe	286 (1,197)	26.0	0.7	0.6	11.0	2.3	0.0	12.0	24	E
**167**	Mortadella mit Paprika	Rewe	259 (1,084)	23.0	0.9	0.8	11.0	2.1	0.0	12.0	23	E
**168**	Mortadella mit Pistazien	Rewe	286 (1,197)	26.0	0.7	0.6	11.0	2.3	0.5	12.0	24	E
**169**	Jagdwurst	Rewe	199 (833)	15.0	0.8	0.7	7.0	2.3	0.5	15.0	19	E
**170**	Bierschinken	Rewe	143 (598)	8.0	0.5	0.5	3.0	2.3	0.5	17.0	14	D
**171**	Italienische Mortadella mit Pistazien	Rewe	276 (1,155)	24.0	0.0	0.0	9.1	2.1	0.0	15.0	22	E
**172**	Delikatess Schinken-Fleischwurst	Rewe	289 (1,209)	26.0	0.5	0.5	11.0	1.9	0.5	13.0	22	E
**173**	Lyoner mit Paprika	Rewe	264 (1,105)	24.0	0.8	0.7	9.0	2.1	0.5	11.0	21	E
**174**	Bayerischer Leberkäse	Rewe	285 (1,192)	26.0	0.5	0.5	11.0	2.0	0.5	12.0	22	E
**175**	Fleischkäsebrät	Rewe	304 (1,272)	27.9	1.0	0.8	10.0	1.8	0.2	13.0	20	E
**Raw sausage products**												
**176**	Mettwurst coarse	BLS	294 (1,230)	24.3	0.2	0.2	9.6	2.4	0.1	19.3	23	E
**177**	Ham salami	BLS	363 (1,519)	31.7	0.2	0.2	12.4	2.8	0.0	20.3	27	E
**178**	German salami	BLS	398 (1,665)	35.6	0.2	0.2	14.0	3.9	0.1	20.3	33	E
**179**	Salami IA, fine	BLS	370 (1,548)	32.7	0.2	0.2	12.9	2.8	0.1	19.7	27	E
**180**	Hungarian-style salami	BLS	381 (1,594)	34.0	0.3	0.3	13.4	2.8	0.0	19.5	27	E
**181**	Italian-style salami	BLS	344 (1,439)	29.3	0.3	0.3	11.6	2.8	0.1	20.7	27	E
**182**	Beef salami	BLS	375 (1,569)	33.3	0.4	0.3	13.3	2.6	0.1	19.5	26	E
**183**	Katenrauch sausage	BLS	366 (1,531)	32.1	0.2	0.2	13.2	2.8	0.1	20.0	27	E
**184**	Venison salami	BLS	305 (1,276)	24.3	0.2	0.2	9.7	2.8	0.0	21.9	26	E
**185**	Salami (pork)	BLS	371 (1,552)	30.9	0.5	0.0	12.3	4.0	0.0	23.5	33	E
**186**	Air-dried mettwurst	BLS	335 (1,402)	28.6	0.2	0.2	11.8	2.8	0.1	19.8	27	E
**187**	Croatian-style salami	BLS	334 (1,397)	26.0	0.3	0.2	10.2	3.3	0.1	25.3	30	E
**188**	Country-style mettwurst	BLS	310 (1,297)	24.9	0.2	0.2	10.2	2.9	0.0	21.8	27	E
**189**	Cervelat sausage	BLS	391 (1,636)	34.8	0.3	0.3	14.6	2.8	0.1	20.3	28	E
**190**	Ham plockwurst	BLS	397 (1,661)	31.9	0.3	0.2	13.1	3.7	0.1	28.1	32	E

No.	Product	Source	Energy (kcal/100 g) (kJ/100 g)	Fat (g/100 g)	Carbohydrates (g/100 g)	CHO (g/100 g)	Sat. Fat (g/ 00 g)	Salt (g/100 g)	Fibre (g/100 g)	Protein (g/100 g)	FSAm-NPS-Score	Nutri-Score
191	Raw sausage, coarse-grained sausages	BLS	277 (1,159)	21.4	0.3	0.2	8.4	3.1	0.1	21.3	26	E
192	Black Forest farmer's sausage	BLS	352 (1,473)	31.3	0.1	0.1	12.6	2.5	0.0	18.2	26	E
193	Landjäger sausages	BLS	476 (1,992)	42.2	0.3	0.2	17.6	3.8	0.0	25.1	34	E
194	Cabanossi	BLS	451 (1,887)	43.9	0.3	0.3	18.1	2.4	0.1	15.2	26	E
195	Feinschmecker-Salami	Rewe	355 (1,485)	28.1	1.0	1.0	11.0	4.1	0.0	24.1	34	E
196	il Salame di Milano L'Originale	Rewe	383 (1,602)	31.0	0.5	0.5	12.0	4.1	0.0	26.0	34	E
197	Delikatess Gourmet Salami	Rewe	315 (1,318)	25.0	0.5	0.5	11.0	3.5	0.0	22.0	30	E
198	Rein Rind Salami	Rewe	270 (1,130)	20.0	1.0	1.0	9.5	3.7	0.0	21.0	30	E
199	Pfeffer-Salami	Rewe	374 (1,565)	30.0	1.0	1.0	12.0	4.0	1.0	24.5	33	E
200	Edel Salami	Rewe	318 (1,331)	24.0	1.0	1.0	10.0	3.8	0.0	24.0	30	E
201	Salami Spitzenqualität	Rewe	314 (1,314)	25.0	1.0	1.0	10.0	3.5	0.5	21.0	29	E
202	Peperoni Salami	Rewe	412 (1,724)	35.4	1.0	1.0	14.0	4.9	0.0	21.8	35	E
203	Delikatess Pfeffer Salami	Rewe	319 (1,335)	25.0	1.5	1.5	11.0	3.4	0.0	22.0	29	E
204	Rindfleisch-Salami	Rewe	249 (1,042)	18.5	1.0	0.3	7.2	4.0	0.0	20.0	29	E
205	Genießer-Salami	Rewe	333 (1,393)	27.0	0.5	0.5	11.0	3.7	0.0	22.0	32	E
206	Genießer-Salami im Pfeffermantel	Rewe	337 (1,410)	27.0	0.5	0.5	11.0	3.8	0.0	22.0	32	E
207	Stickado Hot Chili	Rewe	524 (2,192)	44.0	4.0	2.0	17.0	4.4	0.0	28.0	36	E
208	Sommerwurst	Rewe	341 (1,427)	28.0	1.0	0.5	11.2	4.0	0.5	21.0	33	E
209	Original spanische Chorizo-Salami	Rewe	316 (1,322)	25.0	2.7	2.7	9.0	3.5	0.0	20.0	28	E
210	Premium Gourmet-Salami	Rewe	308 (1,289)	24.0	0.5	0.5	10.1	3.9	0.0	22.4	32	E
211	Ring-Salami	Rewe	445 (1,862)	37.0	2.0	1.5	15.0	5.0	0.0	26.0	35	E
212	Salame Milano	Rewe	371 (1,552)	29.0	0.5	0.5	9.9	4.0	0.0	27.0	32	E
213	Kabanos Klassik	Rewe	297 (1,243)	24.0	1.5	1.0	10.0	2.9	0.5	18.5	26	E
214	Chorizo	Rewe	316 (1,322)	25.0	2.7	2.7	9.0	3.5	0.0	20.0	28	E
215	Baguette Salami	Rewe	387 (1,619)	30.0	1.0	1.0	11.0	4.1	0.0	28.0	34	E
216	Delikatess Feinschmecker Salami	Rewe	314 (1,314)	24.0	0.5	0.5	10.1	3.8	0.0	24.1	31	E
217	Delikatess Paprika Salami	Rewe	354 (1,481)	30.0	1.0	0.5	12.6	3.5	0.0	20.0	31	E
218	Landjäger	Rewe	372 (1,556)	27.2	0.9	0.9	11.2	4.4	0.0	31.0	34	E
219	Delikatess Salami	Rewe	306 (1,280)	25.0	1.0	1.0	10.0	3.5	0.5	19.0	29	E

No.	Product	Source	Energy (kcal/100 g) (kJ/100 g)	Fat (g/100 g)	Carbohydrates (g/100 g)	CHO (g/100 g)	Sat. Fat (g/ 00 g)	Salt (g/100 g)	Fibre (g/100 g)	Protein (g/100 g)	FSAm-NPS-Score	Nutri-Score
**220**	Edel-Salami	Rewe	403 (1,686)	35.0	1.0	1.0	15.0	4.0	0.0	21.0	33	E
**221**	Baguette Salami	Rewe	385 (1,611)	31.0	0.5	0.5	13.0	4.0	0.0	26.0	33	E
**222**	Peitschen Classic	Rewe	482 (2,017)	38.7	2.5	2.3	15.6	4.5	0.0	31.0	35	E
**223**	Bifi Chorizo with Paprika	Rewe	496 (2,075)	44.0	0.5	0.5	18.0	4.2	0.0	25.0	36	E
**224**	Bifi The Original	Rewe	514 (2,151)	46.0	1.0	0.9	20.0	4.2	0.0	24.0	36	E
**225**	Knabber Sticks	Rewe	507 (2,121)	45.0	0.5	0.5	18.0	4.5	0.0	25.0	36	E
**226**	Würzige Tyrolini herzhaft	Rewe	500 (2,092)	42.0	0.5	0.5	17.0	4.5	0.0	30.0	36	E
**227**	Milde Tyrolini	Rewe	500 (2,092)	42.0	0.5	0.5	17.0	4.5	0.0	30.0	36	E
**228**	Rinder Tyrolini Classic	Rewe	381 (1,594)	27.0	0.5	0.5	11.0	4.5	0.0	34.0	34	E
**229**	Mini Salami	Rewe	431 (1,803)	33.0	1.0	0.2	14.0	4.1	0.0	32.0	35	E
**230**	Stickado Salami-Sticks Classique	Rewe	530 (2,218)	44.0	4.1	3.2	18.0	5.0	0.0	29.0	36	E
**231**	Tyrolini würzig	Rewe	500 (2,092)	42.0	0.5	0.5	17.0	4.5	0.0	30.0	36	E
**232**	Salami-Sticks Classic	Rewe	507 (2,121)	41.0	1.0	0.4	18.0	5.0	1.0	33.0	36	E
**233**	Tiroler Kaminwurzerl Das Original	Rewe	433 (1,812)	35.0	0.5	0.5	14.0	4.5	0.0	29.0	35	E
**234**	Peitschen mit Knoblauch	Rewe	482 (2,017)	38.7	2.5	2.3	15.6	4.5	0.0	31.0	35	E
**235**	Tiroler Chiliwurzerl mit Bird's Eye Chili	Rewe	433 (1,812)	35.0	0.5	0.5	14.0	4.5	0.0	29.0	26	E
**236**	Salami Sticks pikant	Rewe	505 (2,113)	41.0	0.5	1.0	15.8	4.5	0.4	33.0	36	E
**237**	Delikatess Mini Salami	Rewe	436 (1,824)	37.0	0.5	0.5	15.0	4.2	0.5	25.0	35	E
**238**	Hähnchen-Salami	Rewe	287 (1,201)	21.0	1.0	0.5	10.5	3.7	0.0	23.0	31	E
**239**	Geflügel-Salami	Rewe	309 (1,293)	23.0	1.0	1.0	9.0	4.0	0.0	24.0	30	E
**240**	Puten-Salami	Rewe	296 (1,238)	22.0	1.0	0.5	11.0	3.7	0.0	23.0	31	E
**241**	Hähnchen Salami	Rewe	243 (1,017)	17.0	0.5	0.5	8.5	3.5	0.0	22.0	28	E
**242**	Hähnchensalami	Rewe	276 (1,155)	20.0	1.0	0.3	6.7	4.0	0.0	23.0	28	E
**Bacon / Ham products**												
**243**	Pork ham bacon, raw, smoked	BLS	152 (636)	7.7	0.0	0.0	2.9	0.1	0.0	20.7	1	B
**244**	Pork streaky bacon, raw, smoked	BLS	320 (1,339)	28.9	0.0	0.0	11.9	0.1	0.0	16.0	13	D
**245**	Pork front/rear ham	BLS	121 (506)	4.3	1.1	1.1	1.5	2.7	0.0	19.5	15	D
**246**	Pork ham, cooked, smoked	BLS	121 (506)	4.3	1.1	1.1	1.5	2.7	0.0	19.5	15	D
**247**	Pork ham, cooked, unsmoked, fried (prepared without added fat)	BLS	156 (653)	7.3	2.3	2.3	2.7	1.8	0.0	20.2	12	D

No.	Product	Source	Energy (kcal/100 g) (kJ/100 g)	Fat (g/100 g)	Carbohydrates (g/100 g)	CHO (g/100 g)	Sat. Fat (g/ 00 g)	Salt (g/100 g)	Fibre (g/100 g)	Protein (g/100 g)	FSAm-NPS-Score	Nutri-Score
**248**	Black Forest ham	BLS	301 (1,259)	21.9	0.0	0.0	7.6	5.4	0.0	26.5	30	E
**249**	Loin ham	BLS	116 (485)	4.4	0.9	0.9	1.6	5.3	0.0	18.3	22	E
**250**	Delikatess Bacon	Rewe	328 (1,372)	30.0	0.5	0.5	13.0	2.4	0.0	14.0	25	E
**251**	Tiroler Schinkenspeck Würfel mild würzig	Rewe	289 (1,209)	19.0	0.5	0.5	7.6	4.0	0.0	29.0	29	E
**252**	Delikatess Lachs Schinken	Rewe	121 (506)	2.0	0.5	0.5	1.0	4.0	0.0	25.0	20	E
**253**	Original Bacon Scheiben	Rewe	308 (1,289)	28.0	0.0	0.0	11.0	2.6	0.0	14.0	25	E
**254**	Schinken Nuggetz	Rewe	140 (586)	3.0	1.0	0.5	1.2	4.0	0.0	27.0	21	E
**255**	Katenschinken-Würfel Mager	Rewe	114 (477)	2.0	1.0	1.0	0.7	4.5	0.0	23.0	21	E
**256**	Delikatess Schinken in Würfeln	Rewe	246 (1,029)	18.0	1.0	0.9	8.0	4.5	0.0	20.0	30	E
**257**	Delikatess Bacon in Scheiben	Rewe	324 (1,356)	29.0	0.5	0.5	12.0	2.4	0.5	15.0	25	E
**258**	Delikatess Lachsschinken	Rewe	124 (519)	2.0	0.5	0.5	1.0	3.8	0.0	26.0	19	E
**259**	Delikatess Bacon in Streifen	Rewe	288 (1,205)	25.0	0.5	0.5	9.0	2.4	0.0	15.0	22	E
**260**	Delikatess Kochschinken	Rewe	101 (423)	2.0	0.5	0.5	0.8	2.1	0.5	20.0	11	D
**261**	Black Angus Bresaola	Rewe	180 (753)	6.0	0.5	0.5	2.9	3.6	0.0	31.0	21	E
Spreadable sausage products												
**262**	Calf liver sausage	BLS	345 (1,443)	32.0	1.7	0.0	12.6	1.9	0.0	13.6	23	E
**263**	Delicatessen liver sausage	BLS	333 (1,393)	29.9	1.0	0.5	12.2	1.4	0.1	16.0	21	E
**264**	Liver sausage, coarse	BLS	354 (1,481)	33.2	0.9	0.6	13.1	1.4	0.1	13.9	21	E
**265**	Farmhouse liver sausage	BLS	364 (1,523)	32.8	0.6	0.3	13.6	1.5	0.1	17.5	21	E
**266**	Estate liver sausage	BLS	327 (1,368)	28.2	0.9	0.6	11.5	1.5	0.1	18.1	21	E
**267**	Calf liver sausage, coarse	BLS	348 (1,456)	31.9	0.8	0.6	13.1	1.5	0.1	15.3	21	E
**268**	Onion liver sausage, simple	BLS	331 (1,385)	31.4	0.5	0.4	12.8	1.5	0.1	12.5	21	E
**269**	Herb liver sausage	BLS	345 (1,443)	32.1	0.9	0.5	13.1	1.6	0.2	14.4	21	E
**270**	Teewurst and other spreadable raw sausages	BLS	456 (1,908)	45.3	1.6	1.5	17.5	1.0	0.1	12.0	20	E
**271**	Teewurst	BLS	456 (1,908)	45.3	1.6	1.5	14.2	2.9	0.1	12.0	29	E
**272**	Teewurst, coarse	BLS	456 (1,908)	45.3	1.6	1.5	14.2	2.9	0.1	12.0	29	E
**273**	Teewurst, Rügenwald style	BLS	456 (1,908)	45.3	1.7	1.6	14.2	2.9	0.0	12.0	29	E
**274**	Spreadable mettwurst	BLS	388 (1,623)	35.9	0.2	0.2	14.1	2.1	0.1	17.1	24	E
**275**	Spreadable mettwurst (fatty mettwurst)	BLS	382 (1,598)	36.9	0.2	0.2	15.4	2.0	0.1	13.5	23	E

No.	Product	Source	Energy (kcal/100 g) (kJ/100 g)	Fat (g/100 g)	Carbohydrates (g/100 g)	CHO (g/100 g)	Sat. Fat (g/ 00 g)	Salt (g/100 g)	Fibre (g/100 g)	Protein (g/100 g)	FSAm-NPS-Score	Nutri-Score
276	Spreadable mettwurst, simple	BLS	414 (1,732)	40.5	0.3	0.3	17.2	2.4	0.1	13.5	27	E
277	Onion sausage	BLS	407 (1,703)	40.3	1.3	1.1	16.6	1.8	0.4	11.1	23	E
278	Poultry liver sausage	BLS	244 (1,021)	16.8	2.9	0.4	6.6	1.9	0.1	20.4	18	D
279	Rügenwälder Feine Teewurst	Rewe	419 (1,753)	41.0	0.5	0.5	15.8	2.5	0.0	12.0	27	E
280	Bärchen Streich	Rewe	337 (1,410)	30.0	1.0	0.5	12.8	1.8	0.0	11.0	22	E
281	Pommersche Feine Gutsleberwurst Apfel und Zwiebel	Rewe	284 (1,188)	24.0	3.9	3.9	9.5	1.8	0.0	13.0	21	E
282	Pommersche Feine Gutsleberwurst	Rewe	311 (1,301)	28.0	0.7	0.7	11.2	1.9	0.0	14.0	22	E
283	Rügenwalder Teewurst fein	Rewe	429 (1,795)	42.0	0.5	0.5	16.0	2.8	0.5	12.0	28	E
284	Feine Pommersche Gutsleberwurst	Rewe	368 (1,540)	35.0	1.0	1.0	15.0	1.7	0.7	12.0	22	E
285	Mühlen Mett Zwiebelmettwurst	Rewe	198 (828)	14.0	1.0	1.0	5.7	3.2	0.0	17.0	22	E
286	Pommersche Feine Gutsleberwurst Schnittlauch	Rewe	311 (1,301)	28.0	0.7	0.7	12.2	2.0	0.0	14.0	22	E
287	Delikatess Leberwurst fein	Rewe	373 (1,561)	35.0	2.5	1.4	15.0	1.8	0.0	12.0	22	E
288	Delikatess Teewurst fein	Rewe	414 (1,732)	40.0	0.5	0.5	16.0	2.8	0.0	13.0	28	E
289	Kräuter-Leberwurst	Rewe	353 (1,477)	33.0	1.0	1.0	13.2	2.5	0.0	13.0	26	E
290	Rügenwalder Teewurst grob	Rewe	356 (1,490)	33.0	0.5	0.5	13.0	2.8	0.5	14.0	27	E
291	Pikante Zwiebelmettwurst fein	Rewe	280 (1,172)	24.0	1.0	1.0	9.6	2.5	0.0	15.0	24	E
292	Bärchen Geflügel-Streich	Rewe	275 (1,151)	25.0	2.1	0.1	20.5	1.8	0.0	10.4	21	E
293	Pommersche Feine Leberwurst Geflügel	Rewe	262 (1,096)	21.0	1.4	1.0	6.3	1.5	5.6	14.0	13	D
294	Holsteiner Puten Frische Zwiebelmettwurst	Rewe	134 (561)	6.3	0.7	0.6	2.2	2.8	0.0	18.6	16	D
